# Supplementary material for: Charge-transfer complexation of coordination cages for enhanced photochromism and photocatalysis
Source: Nat Commun. 2025 Jan 9;16:546. doi: 10.1038/s41467-025-55893-z (PMC11718061; doi:10.1038/s41467-025-55893-z)
Supplement: Supplementary file 1 — Supplementary Information [file 41467_2025_55893_MOESM1_ESM.pdf]

Supplementary Information for

Charge-Transfer Complexation of Coordination Cages for Enhanced Photochromism and Photocatalysis

Gen Li,[1] Zelin Du,[1] Chao Wu,[2] Yawei Liu,[1] Yan Xu,[1] Roy Lavendomme,[3,4] Shihang Liang,[5] En-Qing Gao,[1,6]* and Dawei Zhang[1,6]*

[1] State Key Laboratory of Petroleum Molecular & Process Engineering, Shanghai Key Laboratory of Green Chemistry and Chemical Processes, School of Chemistry and Molecular Engineering, East China Normal University, Shanghai 200062, China.

[2] Department of Computer Science, Durham University, Durham, DH1 3LE, United Kingdom.

[3] Laboratoire de Chimie Organique, Université libre de Bruxelles (ULB), Avenue F. D. Roosevelt 50, CP160/06, B-1050 Brussels, Belgium.

[4] Laboratoire de Résonance Magnétique Nucléaire Haute Résolution, Université libre de Bruxelles (ULB), Avenue F. D. Roosevelt 50, CP160/08, B-1050 Brussels, Belgium.

[5] State Key Laboratory of Petroleum Molecular & Process Engineering, SINOPEC Research Institute of Petroleum Processing, Beijing 100083, P. R. China.

[6] Institute of Eco-Chongming, Shanghai 202162 (P. R. China).

*Corresponding authors: eqgao@chem.ecnu.edu.cn (E.-Q.G); dwzhang@chem.ecnu.edu.cn (D.Z.)

Table of Contents

1. Supplementary Note 1: Synthesis and characterization of ligands 3

1.1 Synthesis and characterization of **L**^{1}-BAr_F 3

1.2 Synthesis and characterization of **L**^{2}-BAr_F 5

1.3 Synthesis and characterization of **L**^{3}-BAr_F 7

1.4 Synthesis and characterization of **L**^{4}-BAr_F 10

2. Supplementary Note 2: Synthesis and characterization of Zr-MOCs 13

2.1 Synthesis and characterization of cage **1** 13

2.2 Synthesis and characterization of cage **2** 18

2.3 Synthesis and characterization of cage **3** 25

2.4 Synthesis and characterization of cage **4** 29

3. Supplementary Note 3: X-ray crystallography..33

4. Supplementary Note 4: Guest binding studies...41

4.1 ^{1}H NMR titrations of common anions..42

4.2 ^{1}H NMR titrations of tetraarylborate anions..52

4.3 UV-vis spectrophotometric titrations 57

5. Supplementary Note 5: Photochromism studies 58

5.1 Photochromism of **1** in the presence of tetraarylborates...58

5.2 Effect of bound anions on photochromism of **1**-BAr_F...63

6. Supplementary Note 6: Photocatalytic transformation of tetraarylborates.........................66

6.1 Effect of anions and solvent on photocatalysis 66

6.2 Investigation of catalytic mechanism 67

7. Supplementary Note 7: Performances of cage in comparison to ligand 69

8. Supplementary References 74

1. Supplementary Note 1: Synthesis and characterization of ligands

1.1 Synthesis and characterization of L^1-BAr$_F$

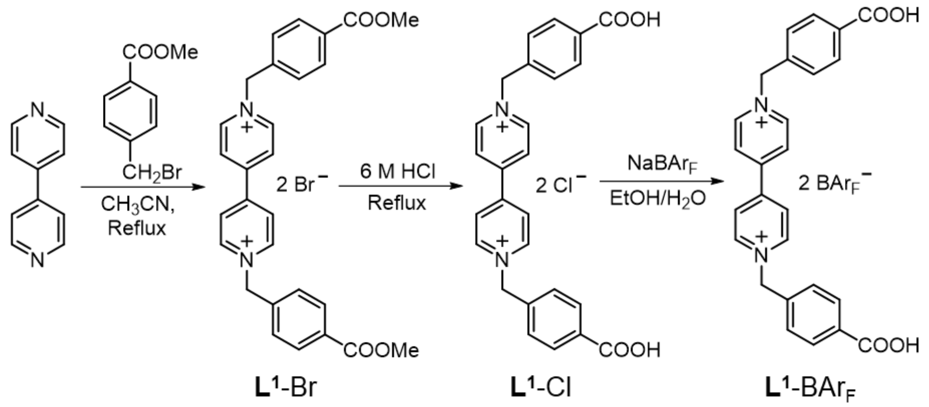

Supplementary Fig. 1 | Synthesis of L^1-BAr$_F$.

L^1-Br and **L^1**-Cl were synthesized according to the reported procedures.[1]

L^1-Br: 4-(Bromomethyl) benzoate methyl ester (2.93 g, 12.8 mmol) was added to a solution of 4,4′-bipyridine (1.00 g, 6.40 mmol) in 20 mL acetonitrile. The mixture was refluxed for 12 h. After completion of the reaction, the mixture was cooled to room temperature and was filtered. The filter cake was rinsed with CH_3CN (10 mL × 5) and then was dried in a vacuum oven to give the dimethyl ester (**L^1**-Br) as light-yellow powder (3.7 g, 95% yield). **^{1}H NMR** (CD_3OD, 400 MHz, 298 K): δ 9.22 (d, J = 8.4 Hz, 4H), 8.59 (d, J = 8.4 Hz, 4H), 8.10 (d, J = 10.2 Hz, 4H), 7.74 (d, J = 10.2 Hz, 4H), 6.07 (s, 4H), 3.94 (s, 6H) ppm.

L^1-Cl: A solution of **L^1**-Br (1.00 g, 1.60 mmol) in hydrochloric acid (20 mL, 6.0 mol/L) was refluxed for 12 h. The solution was cooled to room temperature and then was kept in the refrigerator for 1.0 h. The white powder precipitate was filtered, washed with water and acetone, and dried in a vacuum oven, giving **L^1**-Cl in 92% yield (0.74 g). **^{1}H NMR** (CD_3OD, 400 MHz, 298 K): δ 9.29 (d, J = 8.7, 4H), 8.64 (d, J = 8.7, 4H), 8.17 (d, J = 9.8, 4H), 7.62 (d, J = 9.8, 4H), 6.11 (s, 4H) ppm.

L^1-BAr$_F$: **L^1**-BAr$_F$ was obtained through an anion exchange of **L^1**-Cl with NaBAr$_F$. Briefly, 60 mg (0.12 mmol) of **L^1**-Cl and 0.21 g (0.24 mmol) of NaBAr$_F$ were respectively dissolved in 2.0 mL H_2O and 1.0 mL EtOH. Mixing of the two solutions led to a large amount of white precipitate. After filtration and washing with water (10 mL × 3), the precipitate was collected and was dried under vacuum to obtain **L^1**-BAr$_F$ (237 mg, 92% yield). **^{1}H NMR** (CD_3OD, 400 MHz, 298 K): δ 9.37 (d, J = 8.8 Hz, 4H), 8.68 (d, J = 8.8 Hz, 4H), 8.14 (d, J = 10.8 Hz, 4H), 7.64-7.58 (m, 16H), 6.03 (s, 4H) ppm. **^{13}C NMR** (CD_3OD, 100 MHz, 298 K): δ 169.2, 163.3, 152.5, 147.9, 139.3, 132.5, 131.2, 130.7, 129.6, 129.2, 127.5, 125.3, 119.4, 67.1 ppm. **^{19}F NMR** (CD_3OD, 376.4 MHz, 298 K): δ -62.2 ppm.

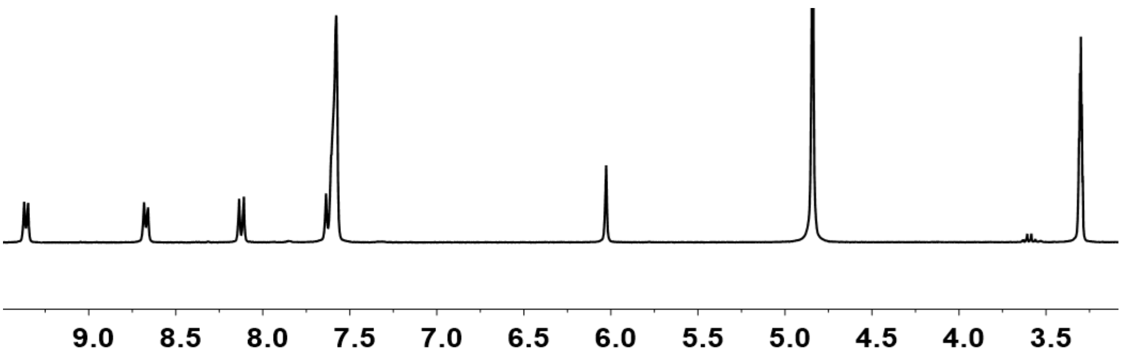

Supplementary Fig. 2 | ^{1}H NMR spectrum (CD_3OD, 400 MHz, 298 K) of **L^{1}**-BAr$_F$.

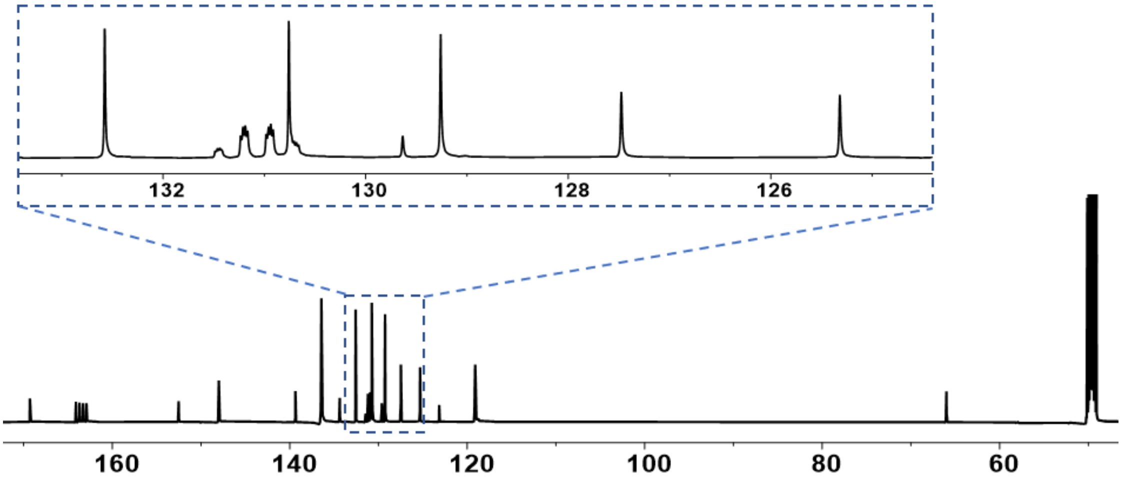

Supplementary Fig. 3 | ^{13}C NMR spectrum (CD_3OD, 100 MHz, 298 K) of **L^{1}**-BAr$_F$.

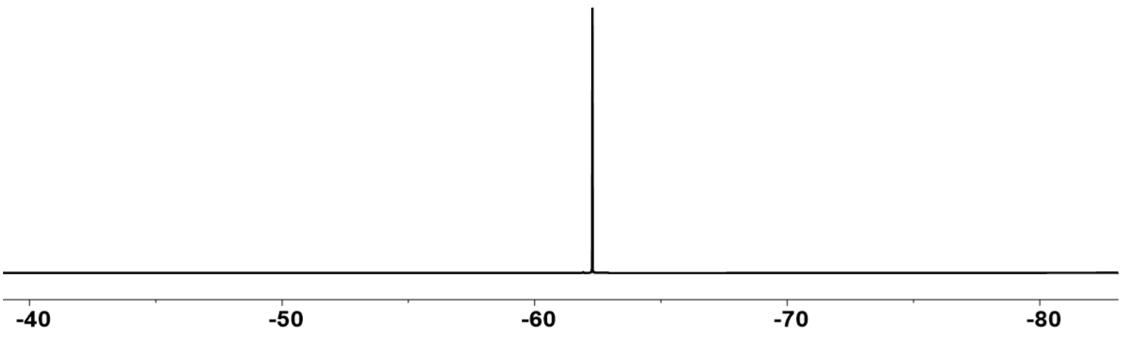

Supplementary Fig. 4 | ^{19}F NMR spectrum (CD_3OD, 376.4 MHz, 298 K) of **L^{1}**-BAr$_F$.

1.2 Synthesis and characterization of L^2-BAr$_F$

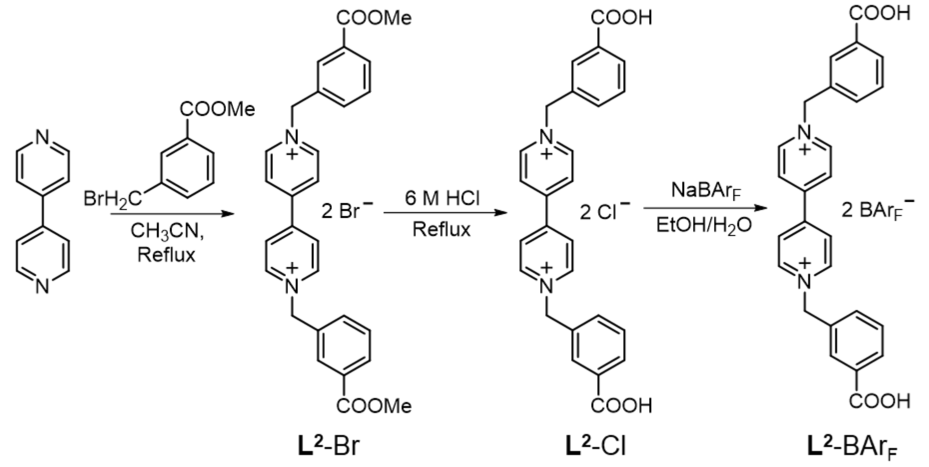

Supplementary Fig. 5 | Synthesis of **L^2**-BAr$_F$.

L^2-Br and **L^2**-Cl were synthesized according to the reported procedures.[1]

L^2-Br: 3-(Bromomethyl) benzoate methyl ester (2.93 g, 12.8 mmol) was added to a solution of 4,4′-bipyridine (1.00 g, 6.4 mmol) in 20 mL acetonitrile. The mixture was refluxed for 12 h. After completion of the reaction, the mixture was cooled to room temperature and was filtered. The filter cake was rinsed with CH_3CN (10 mL × 5) and then was dried in a vacuum oven to give the dimethyl ester (**L^2**-Br) as light-yellow powder (3.61 g, 92% yield). **^{1}H NMR** (CD_3OD, 400 MHz, 298 K): δ 9.28 (d, J = 8.9 Hz, 4H), 8.68 (d, J = 8.9 Hz, 4H), 8.12 (s, 2H), 8.07 (d, J = 8.4 Hz, 2H), 7.69 (m, 4H), 6.05 (s, 4H), 3.96 (s, 6H) ppm.

L^2-Cl: A solution of **L^2**-Br (1.00 g, 1.6 mmol) in hydrochloric acid (20 mL, 6.0 mol/L) was refluxed for 12 h. The solution was cooled to room temperature and then was kept in the refrigerator for 1.0 h. The white powder precipitate was filtrated, washed with water and acetone, and dried in a vacuum oven, giving **L^2**-Cl in 82% yield (0.66 g). **^{1}H NMR** (CD_3OD, 400 MHz, 298 K): δ 9.32 (d, J = 8.5, 4H), 8.73 (d, J = 8.5, 4H), 8.14 (s, 2H), 8.04 (d, J = 8.1 Hz, 2H), 7.73 (m, 4H), 6.07 (s, 4H) ppm.

L^2-BAr$_F$: **L^2**-BAr$_F$ was obtained through an anion exchange of **L^2**-Cl with NaBAr$_F$. Briefly, 0.10 g (0.20 mmol) of **L^2**-Cl and 0.36 g (0.40 mmol) of NaBAr$_F$ were respectively dissolved in 3.0 mL H_2O and 1.0 mL EtOH. Mixing of the two solutions led to a large amount of white precipitate. After filtration and washing with water (10 mL × 5), the precipitate was collected and was dried under vacuum to obtain **L^2**-BAr$_F$ (0.37 g, 85% yield). **^{1}H NMR** (CD_3OD, 298 K, 400 MHz): δ 9.38 (d, J = 8.6 Hz, 4H), 8.67 (d, J = 8.6 Hz, 4H), 8.17 (s, 2H), 8.11 (d, J = 8.2 Hz, 2H), 7.77 (d, J = 8.2 Hz, 2H), 7.58 (m, 14H), 6.02 (s, 4H) ppm. **^{13}C NMR** (CD_3OD, 100 MHz, 298 K): δ 169.3,165.4, 153.4, 149.3, 138.1, 137.3, 136.2, 134.9, 134.3, 134.6, 134.1, 133.5, 132.5, 131.3, 130.8, 128.9, 126.9, 120.6, 68.6 ppm. **^{19}F NMR** (CD_3OD, 376.4 MHz, 298 K): δ -62.4 ppm.

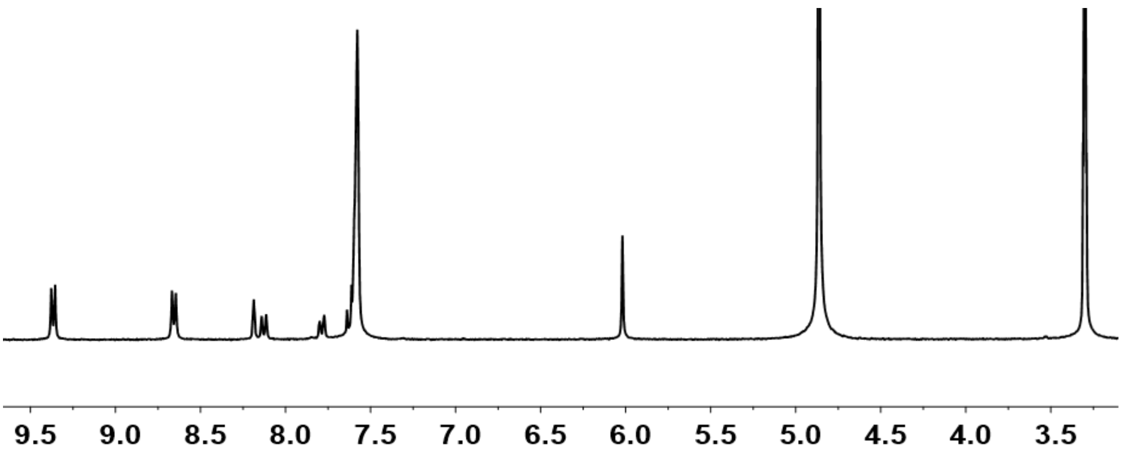

Supplementary Fig. 6 | ^{1}H NMR spectrum (CD_3OD, 400 MHz, 298 K) of **L²**-BAr$_F$.

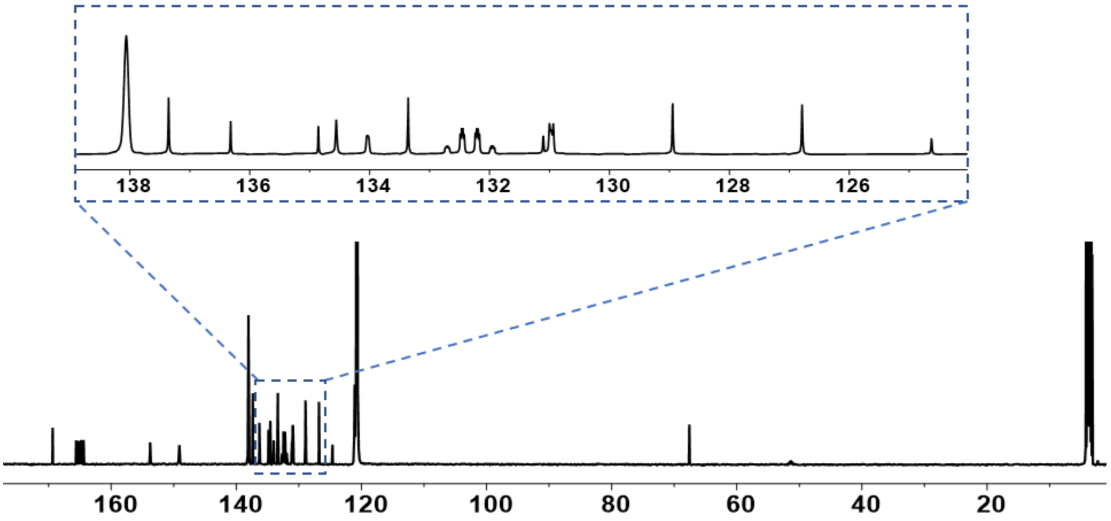

Supplementary Fig. 7 | ^{13}C NMR spectrum (CD_3OD, 100 MHz, 298 K) of **L²**-BAr$_F$.

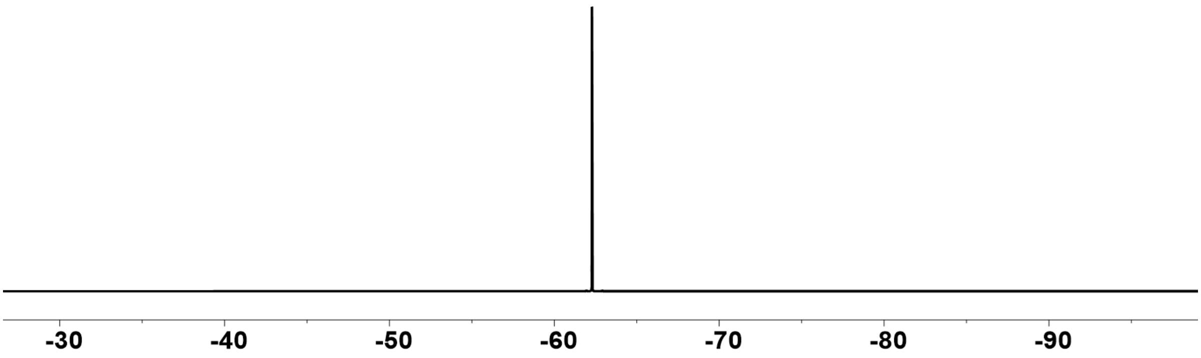

Supplementary Fig. 8 | ^{19}F NMR spectrum (CD_3OD, 376.4 MHz, 298 K) of **L²**-BAr$_F$.

1.3 Synthesis and characterization of L^3-BAr$_F$

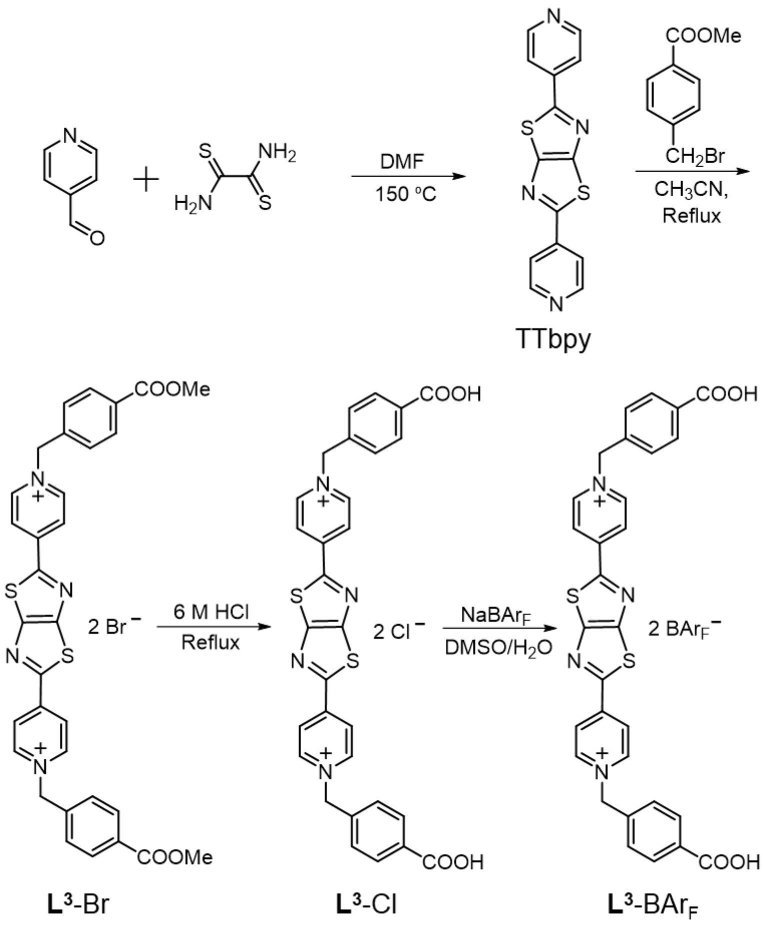

Supplementary Fig. 9 | Synthesis of **L**^{3}-BAr$_F$.

TTbpy: Compound TTbpy was synthesized according to the reported procedures.[2] 4-Pyridine-carboxaldehyde (0.44 g, 5.3 mmol) and dithiooxamide (0.20 g, 1.7 mmol) were stirred at 150 °C in 20 mL anhydrous DMF for 6 h under aerobic conditions. The reaction mixture was allowed to stand overnight, and the precipitated product was collected by filtration and rinsed with water (10 mL × 5). The yellow solid was collected (0.40 g, 50% yield). **^{1}H NMR** ($CDCl_3$, 400 MHz, 298 K): δ 8.73 (d, J = 7.8, 4H), 8.85 (d, J = 7.8, 4H) ppm.

L^3-Br: 4-(Bromomethyl) benzoate methyl ester (0.57 g, 2.5 mmol) was added to a suspension of TTbpy (0.30 g,1.0 mmol) in 80 mL acetonitrile. The mixture was refluxed for 48 h. As the reaction progressed, the color of the reaction mixture gradually changed from gray to yellow. After completion of the reaction, the mixture was cooled to room temperature and was filtered. The filter cake was rinsed with CH_3CN (10 mL × 5) and then was dried in a vacuum oven. Ester **L**^{3}-Br was obtained in a yield of 88% (0.66 g). **^{1}H NMR** (DMSO-d_6, 400 MHz, 298 K): δ 9.37 (d, J = 8.6, 4H), 9.03 (d, J = 8.6, 4H), 8.14 (d, J = 7.9, 4H), 7.82 (d, J = 7.9, 4H), 6.15 (s, 4H), 3.96 (s, 6H) ppm.

L^3-Cl: A solution of **L**^{3}-Br (0.75 g, 1.0 mmol) in hydrochloric acid (50 mL, 6.0 mol/L) was refluxed for 36 h. The solution was cooled to room temperature and then was kept in the refrigerator for 4.0 h. Yellow

solid was collected by filtration, washed with ice water, and dried in a vacuum oven, giving **L³**-Cl in 79% yield (0.53 g). **¹H NMR** (DMSO-*d*6, 400 MHz, 298 K): *δ* 9.28 (d, *J* = 8.3, 4H), 9.06 (d, *J* = 8.3, 4H), 8.12 (d, *J* = 7.8, 4H), 7.93 (d, *J* = 7.8, 4H), 6.09 (s, 4H) ppm.

L³-BArF: L³-BArF was obtained through an anion exchange of **L³**-Cl with NaBArF. Briefly, 60 mg (0.090 mmol) **L³**-Cl and 0.17 g (0.19 mmol) NaBArF were added and dissolved in 10 mL DMSO. 10 mL H_2O was added to the solution mixture and a large amount of yellow precipitate appeared. After filtration and washing with water (3.0 × 10 mL), the precipitate was collected and was dried under vacuum to obtain **L³**-BArF (0.17 g, 83% yield). **¹H NMR** (CD_3OD, 400 MHz, 298 K): *δ* 9.11 (d, *J* = 8.4 Hz, 4H), 8.68 (d, *J* = 8.4 Hz, 4H), 8.05 (d, *J* = 9.6 Hz, 4H), 7.49-7.54 (m, 16H), 5.88 (s, 4H) ppm. **¹³C NMR** (CD_3OD, 100 MHz, 298 K): *δ* 169.3, 167.1, 163.6, 158.1, 149.8, 147.8, 139.6, 136.4, 134.2, 132.5, 131.2, 130.7, 129.6, 126.5, 119.1, 65.5 ppm. **¹⁹F NMR** (CD_3OD, 376.4 MHz, 298 K): *δ* -62.3 ppm.

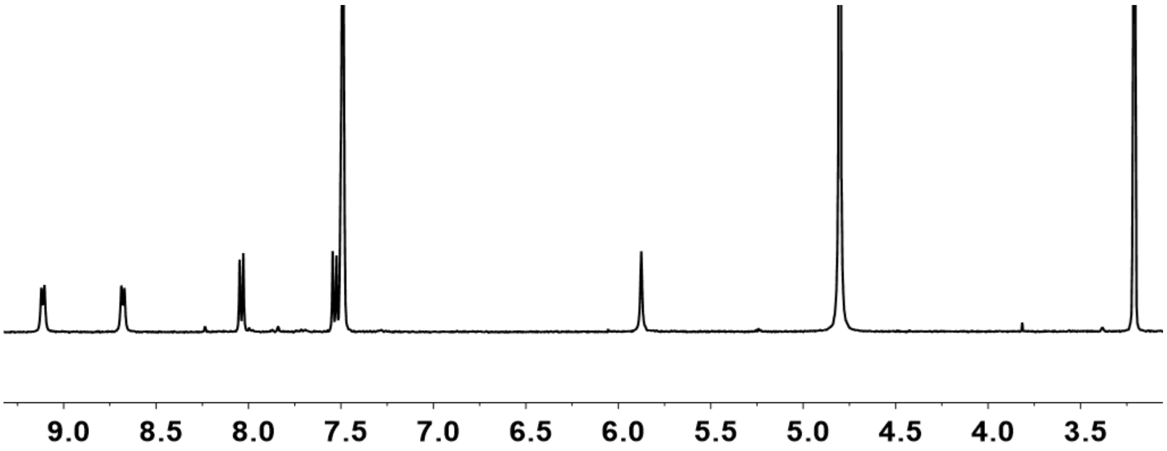

Supplementary Fig. 10 | ¹H NMR spectrum (CD_3OD, 400 MHz, 298 K) of **L³**-BArF.

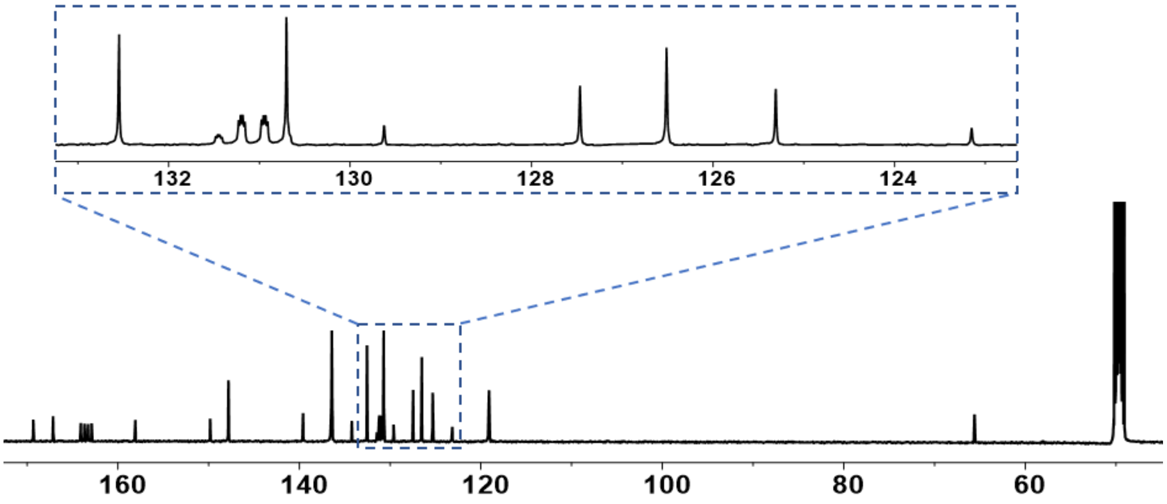

Supplementary Fig. 11 | ¹³C NMR spectrum (CD_3OD, 100 MHz, 298 K) of **L³**-BArF.

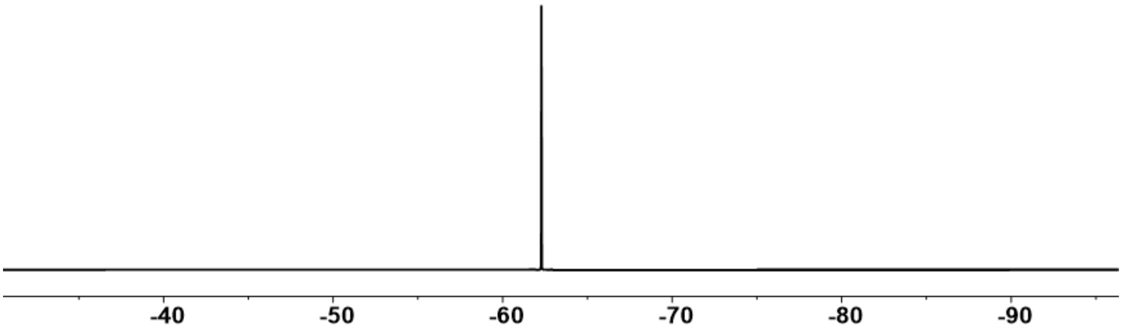

Supplementary Fig. 12 | ^{19}F NMR spectrum (CD_3OD, 376.4 MHz, 298 K) of $\mathbf{L^3}$-BAr_F.

1.4 Synthesis and characterization of L4-BArF

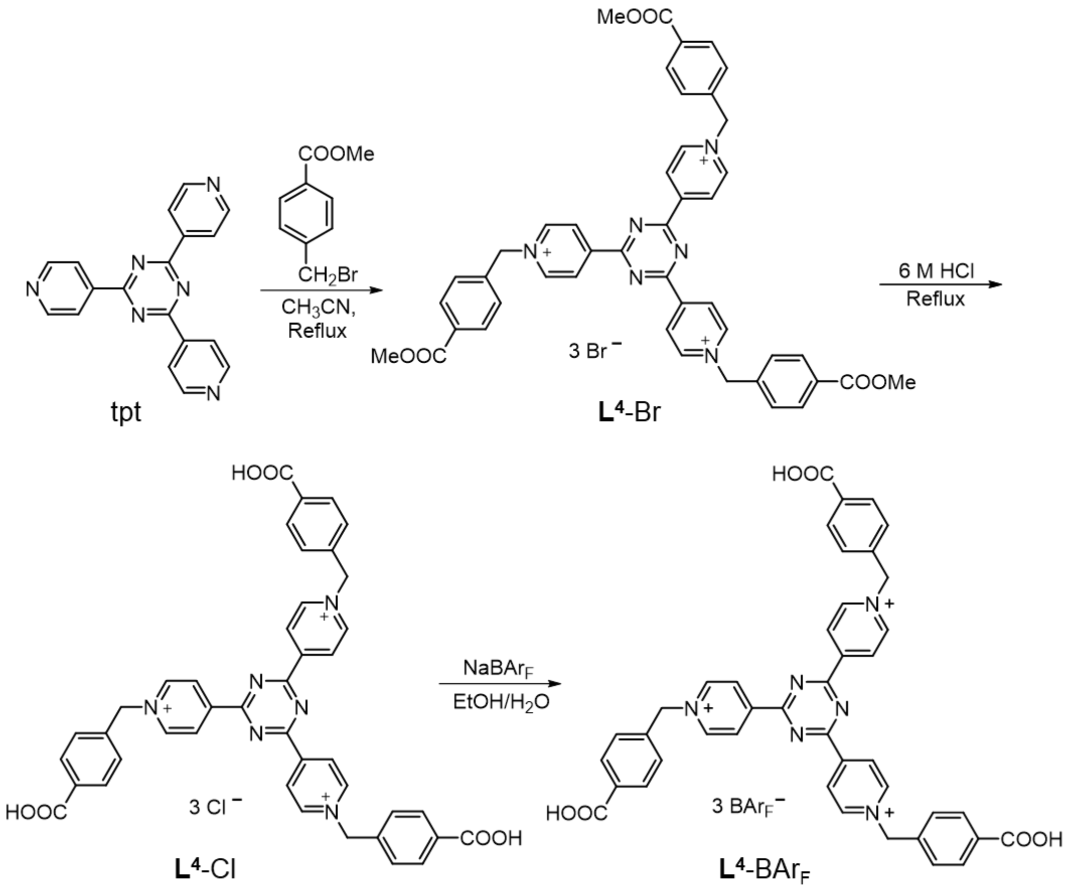

Supplementary Fig. 13 | Synthesis of **L4**-BArF.

L4-Br and **L4**-Cl were synthesized according to the reported procedures.[3]

L4-Br: 4-(Bromomethyl) benzoate methyl ester (0.80 g, 3.5 mmol) was added to a suspension of 2,4,6-tris(4-pyridyl)-1,3,5-triazine (tpt, 0.31 g,1.0 mmol) in 50 mL acetonitrile. The mixture was refluxed for 48 h. As the reaction progressed, the color of the reaction mixture gradually changed from colourless to yellow. After completion of the reaction, the mixture was cooled to room temperature and was filtered. The filter cake was rinsed with CH_3CN (10 mL × 5) and then was dried in a vacuum oven. Ester **L4**-Br was obtained in a yield of 80% (0.80 g). **^{1}H NMR** (DMSO-*d*6, 400 MHz, 298 K): δ 9.67 (d, J = 9.2, 6H), 9.54 (d, J = 9.2, 6H), 8.04 (d, J = 8.2, 6H), 7.87 (d, J = 8.2, 6H), 6.08 (s, 6H), 3.92 (s, 9H) ppm.

L4-Cl: A solution of **L4**-Br (0.50 g, 0.49 mmol) in hydrochloric acid (30 mL, 6.0 mol/L) was refluxed for 12 h. The solution was cooled to room temperature and then was kept in the refrigerator for 2.0 h. Yellow solid was collected by filtration, washed with ice water, and dried in a vacuum oven, giving **L4**-Cl in 83% yield (0.34 g). **^{1}H NMR** (DMSO-*d*6, 400 MHz, 298 K): δ 9.69 (d, J = 9.0, 6H), 9.56 (d, J = 9.0, 6H), 8.16 (d, J = 8.5, 6H), 7.95 (d, J = 8.5, 6H), 6.10 (s, 6H) ppm.

L4-BArF: L4-BArF was obtained through an anion exchange of **L4**-Cl with NaBArF. Briefly, 60 mg (0.073 mmol) **L4**-Cl and 0.19 g (0.22 mmol) NaBArF were respectively dissolved in 2.0 mL H_2O and 1.0 mL

EtOH. Mixing of the two solutions led to a large amount of white precipitate. After filtration and washing with water (10 mL × 3), the precipitate was collected and was dried under vacuum to obtain **L⁴**-BAr$_F$ (0.21 g, 85% yield). **^{1}H NMR** (CD_3OD, 400 MHz, 298 K): *δ* 9.45 (m, *J* = 8.4 Hz, 12H), 8.14 (d, *J* = 8.9 Hz, 6H), 7.67 (d, *J* = 8.9 Hz, 6H), 7.58-7.68 (m, 36H), 6.11 (s, 6H) ppm. **^{13}C NMR** (CD_3OD, 100 MHz, 298 K): *δ* 171.2, 169.4, 163.4, 151.9, 148.5, 139.3, 136.4, 132.7, 131.0, 130.7, 129.3, 125.4, 119.8, 67.7 ppm. **^{19}F NMR** (CD_3OD, 376.4 MHz, 298 K): *δ* -62.6 ppm.

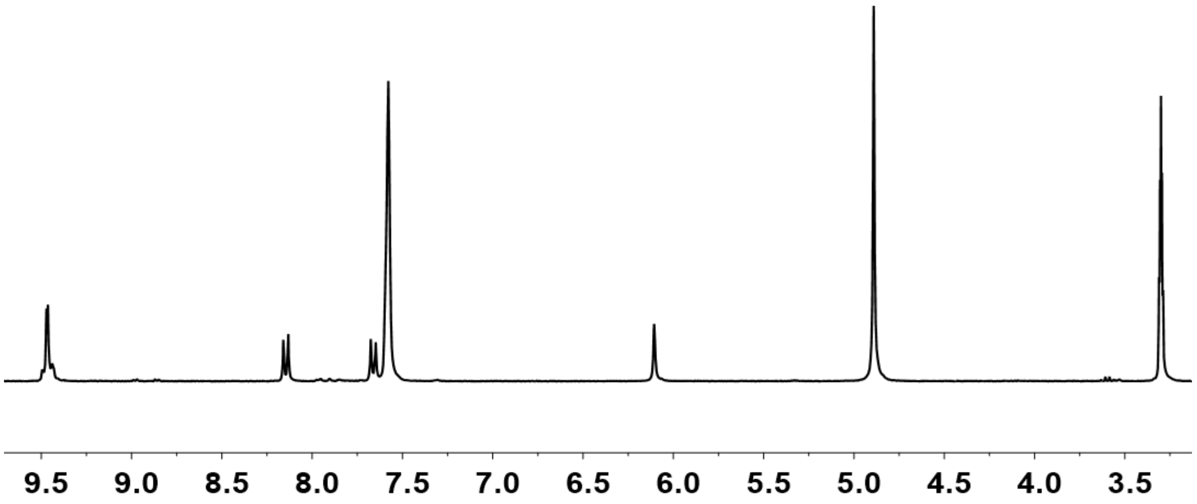

Supplementary Fig. 14 | ^{1}H NMR spectrum (CD_3OD, 400 MHz, 298 K) of **L⁴**-BAr$_F$.

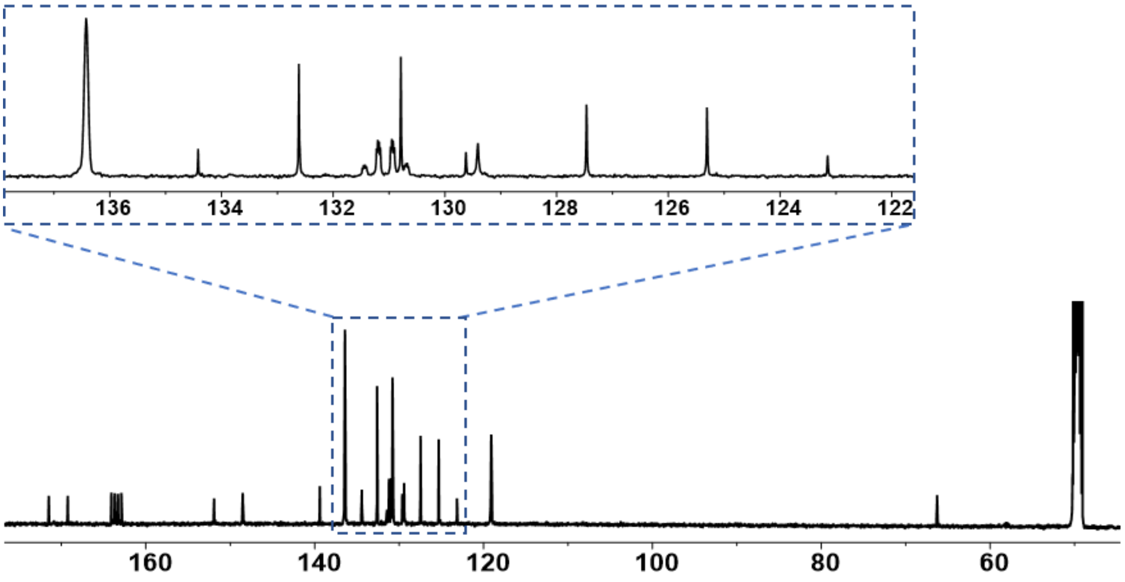

Supplementary Fig. 15 | ^{13}C NMR spectrum (CD_3OD, 100 MHz, 298 K) of **L⁴**-BAr$_F$.

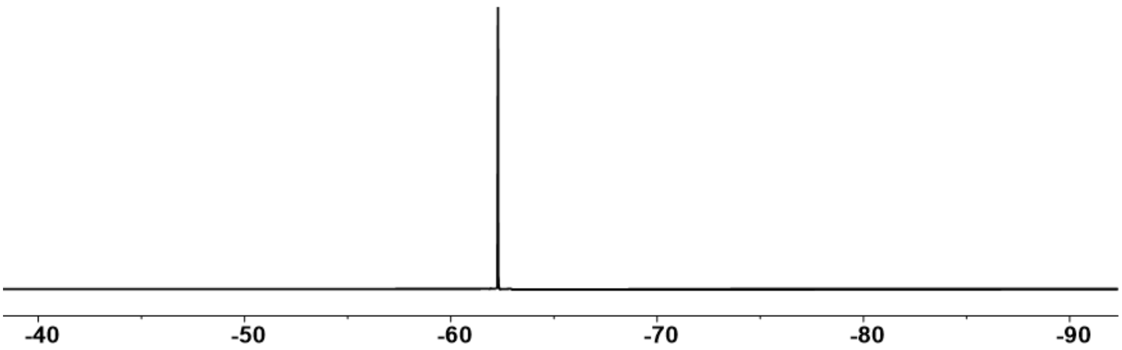

Supplementary Fig. 16 | ^{19}F NMR spectrum (CD_3OD, 376.4 MHz, 298 K) of **L[4]**-BAr_F.

2. Supplementary Note 2: Synthesis and characterization of Zr-MOCs

2.1 Synthesis and characterization of cage 1

1-BAr$_F$: L^1-BAr$_F$ (20 mg, 9.3 μmol, 1.0 equiv.) and Cp_2ZrCl_2 (5.8 mg, 19 μmol, 2.1 equiv.) were added into a solution mixture containing 3.0 mL CH_3OH and 0.12 mL H_2O. The reaction mixture was stirred and kept at 65 °C overnight. After cooling to room temperature, 3.0 mL H_2O was added and a large amount of white precipitate appeared. The precipitate was collected through centrifugation, thoroughly washed with water (5 mL × 3), and dried under vacuum to obtain **1**-BAr$_F$ (21 mg, 74% yield). **^{1}H NMR** (CD_3OD, 400 MHz, 298 K): δ 9.32 (d, J = 9.2 Hz, 12H), 9.05 (d, J = 9.2 Hz, 12H), 7.99 (d, J = 10.8 Hz, 12H), 7.57 (m, 96H), 7.39 (d, J = 10.8 Hz, 12H), 6.55 (s, 30H), 5.91 (*s*, 12H) ppm. **^{13}C NMR** (CD_3OD, 100 MHz, 298 K): δ 173.7, 163.5, 151.7, 148.3, 139.8, 136.4, 132.7, 131.2, 130.9, 129.3, 127.1, 125.3, 123.4, 119.1, 118.3, 66.8 ppm. **^{19}F NMR** (CD_3OD, 376.4 MHz, 298 K): δ -62.1 ppm. **ESI-MS**: *m/z* 1449.1 [M+1Cl$^-$+4BAr$_F^-$]$^{3+}$, 1242.5 [M+1Cl$^-$+3BAr$_F^-$]$^{4+}$. ^{1}H NMR confirmed that there are 8 BAr$_F^-$ anions per cage. Ion chromatography and elemental analysis confirmed that the Cl$^-$ content in **1**-BAr$_F$ is negligible (< 0.2 Cl$^-$ per cage).

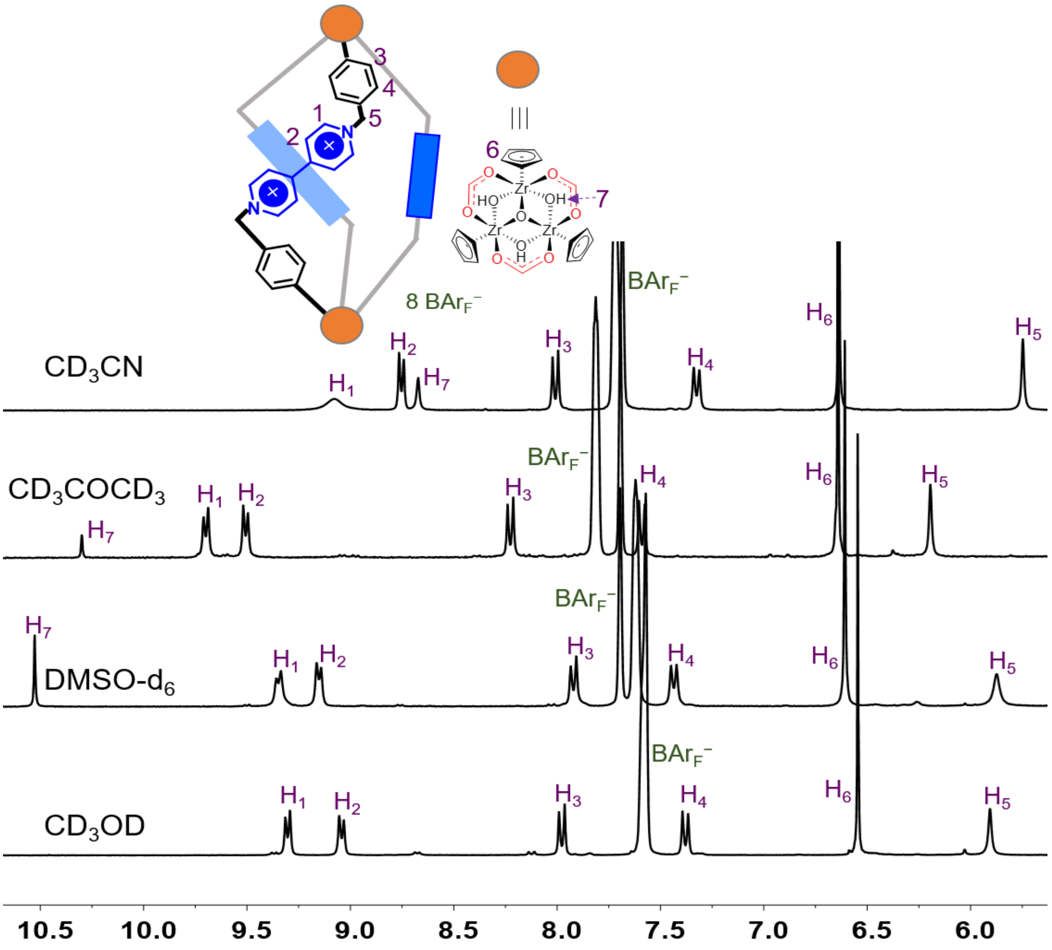

Supplementary Fig. 17 | ^{1}H NMR spectra (400 MHz, 298 K) of **1**-BAr$_F$ in various deuterated solvents.

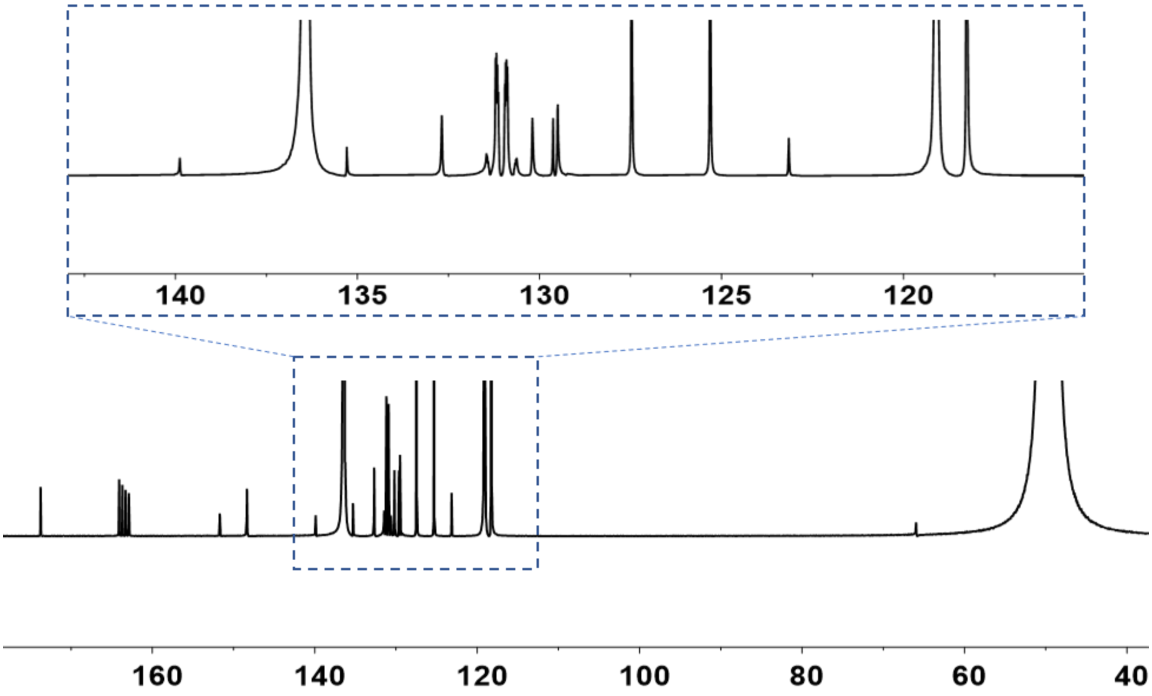

Supplementary Fig. 18 | **^{13}C NMR spectrum (CD_3OD, 100 MHz, 298 K) of **1-BAr_F.

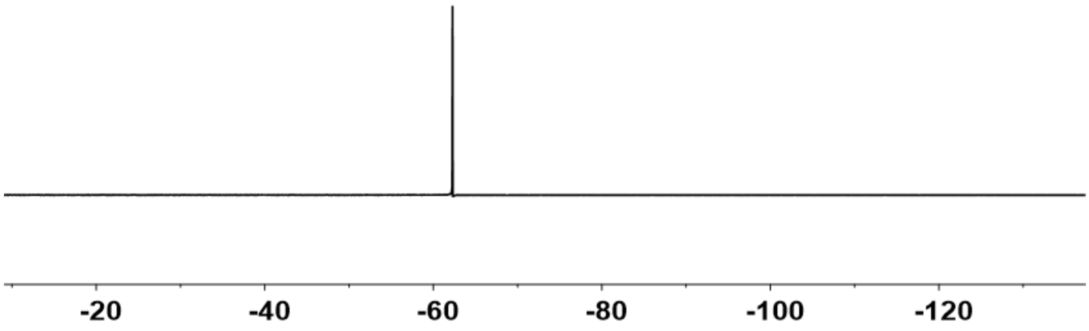

Supplementary Fig. 19 | **^{19}F NMR spectrum (CD_3OD, 376.4 MHz, 298 K) of **1-BAr_F.

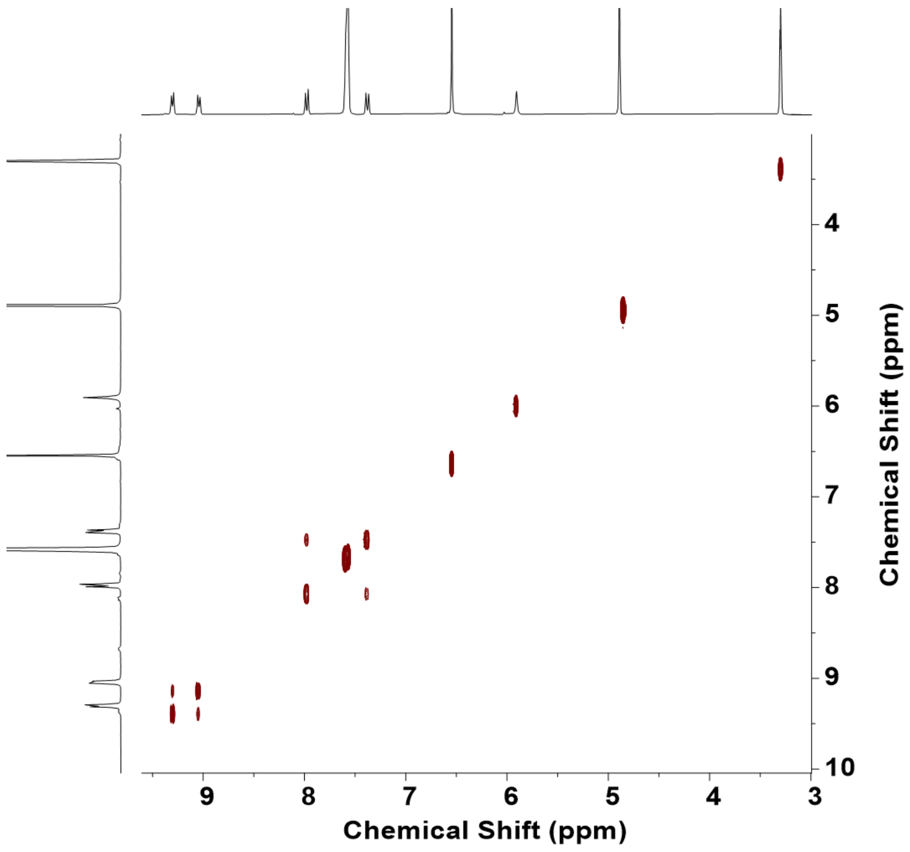

Supplementary Fig. 20 | ^{1}H-^{1}H COSY spectrum (CD_3OD, 400 MHz, 298 K) of **1**-BArF.

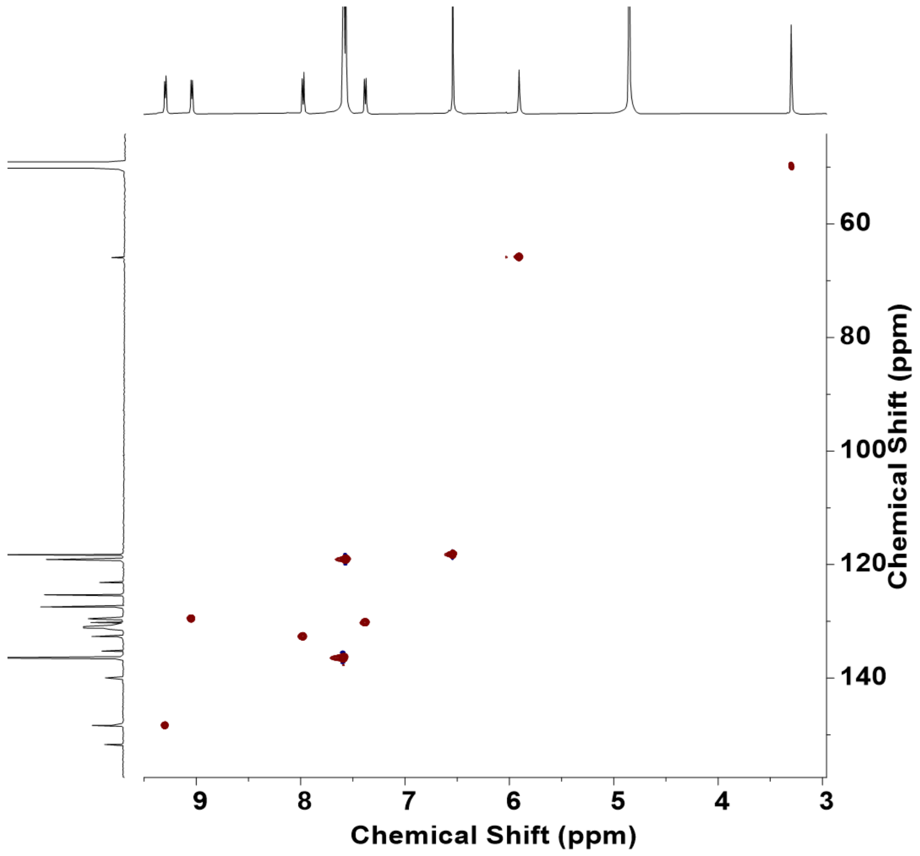

Supplementary Fig. 21 | ^{1}H-^{13}C HSQC spectrum (CD_3OD, 400 MHz, 298 K) of **1**-BArF.

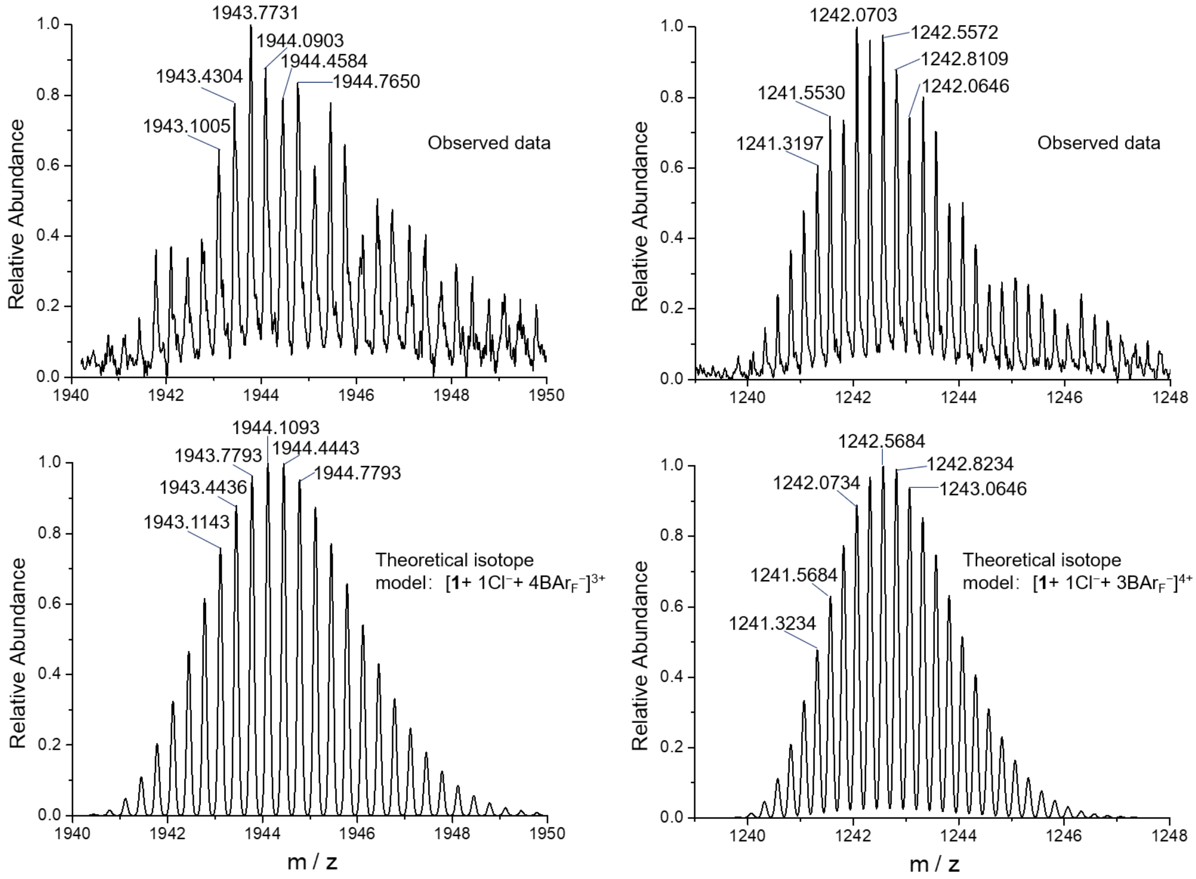

Supplementary Fig. 22 | High-resolution ESI-mass spectrometry analysis of **1**-BAr$_F$ in the presence of TBACl showing the 3+ and 4+ peaks. No reasonable signals were detected for **1**-BAr$_F$ in the absence of TBACl, presumably due to the lower stability of **1** under MS conditions without a suitable anion template.

1-NO$_3$: **1**-BAr$_F$ (50 mg, 5.4 µmol) was dissolved in 2.0 mL CH_3OH. Upon addition of TBANO$_3$ (24 mg, 81 µmol), a large amount of white precipitate appeared immediately. The precipitate was collected by filtration, washed with CH_3OH (3.0 × 10 mL), and dried under vacuum to obtain **1**-NO$_3$ (81% yield).

1-X (X = OTf, PF$_6$, Cl, and NTf$_2$): **1**-NO$_3$ (50 mg, 17 µmol) was dissolved in 5.0 mL H_2O. Upon addition of TBAX (X = OTf, PF$_6$, Cl, or NTf$_2$, 15 equiv.) in 0.5 mL CH_3OH, a large amount of precipitate appeared immediately. For **1**-X (X = OTf, PF$_6$, and NTf$_2$), the precipitate was collected after filtration and washed with H_2O (3 × 10 mL), and was dried under vacuum to obtain **1**-X (> 80% yield). **1**-Cl was collected in a similar way (~ 80% yield), but a smaller amount of water (5 mL) was used for washing because **1**-Cl is modestly soluble in water. The simplified washing for **1**-Cl is sufficient for removing the attached TBACl due to the high solubility of TBACl in water.

1-BPh$_4$: To **1**-BAr$_F$ (50 mg, 5.4 µmol, 1.0 equiv.) in 2.0 mL CH_3OH was added NaBPh$_4$ (15 mg, 43.2 µmol, 8.0 equiv.). 10 mL diethyl ether was added to the mixture, and a large amount of orange precipitate appeared. After filtration and washing with diethyl ether (3.0 × 10 mL), the precipitate was collected and dried under vacuum to obtain **1**-BPh$_4$ (71% yield).

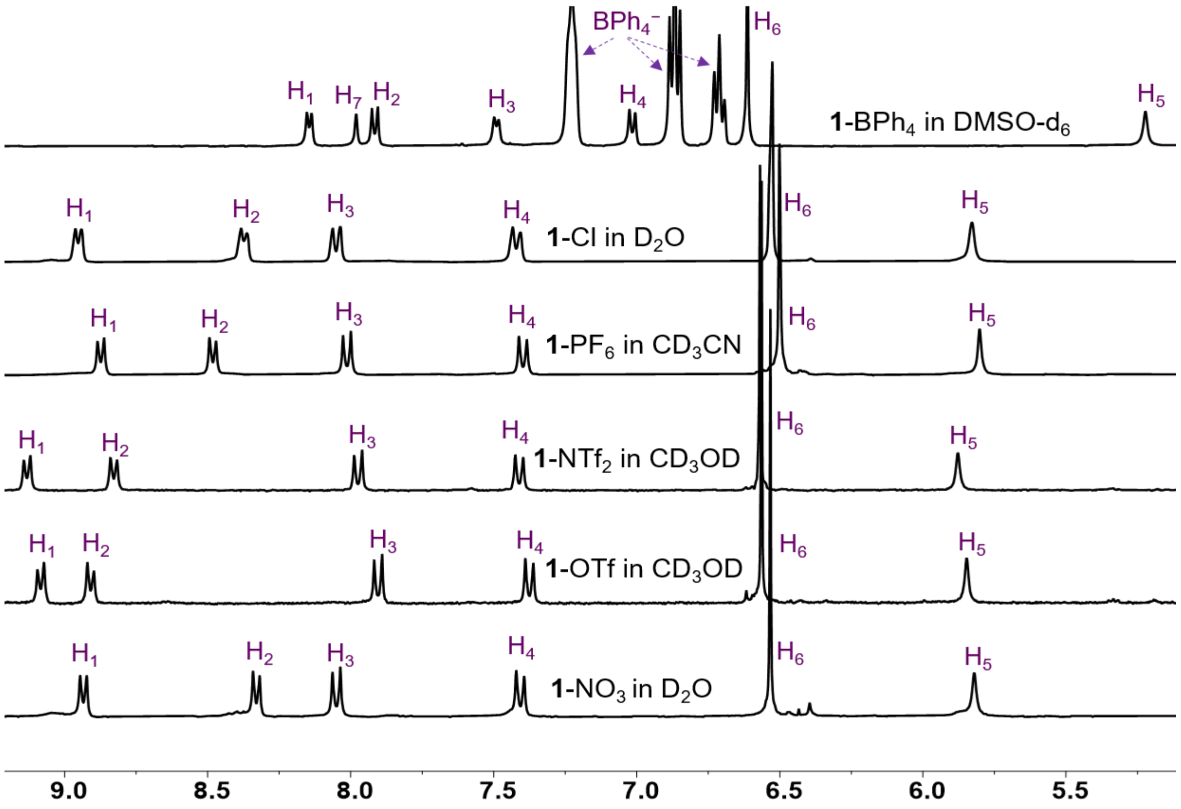

Supplementary Fig. 23 l ^{1}H NMR spectra (400 MHz, 298 K) of **1**-X (X = NO_3, OTf, NTf_2, PF_6, Cl and BPh_4).

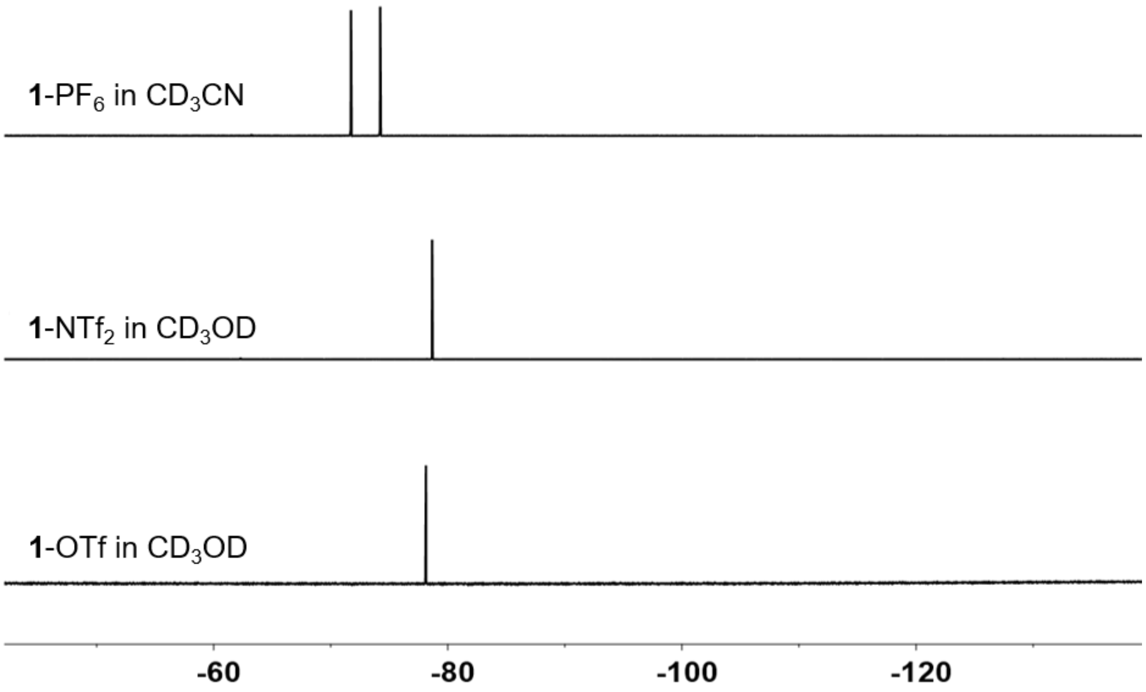

Supplementary Fig. 24 l ^{19}F NMR spectra (376.4 MHz, 298 K) of **1**-X (X = PF_6, NTf_2, and OTf).

2.2 Synthesis and characterization of cage 2

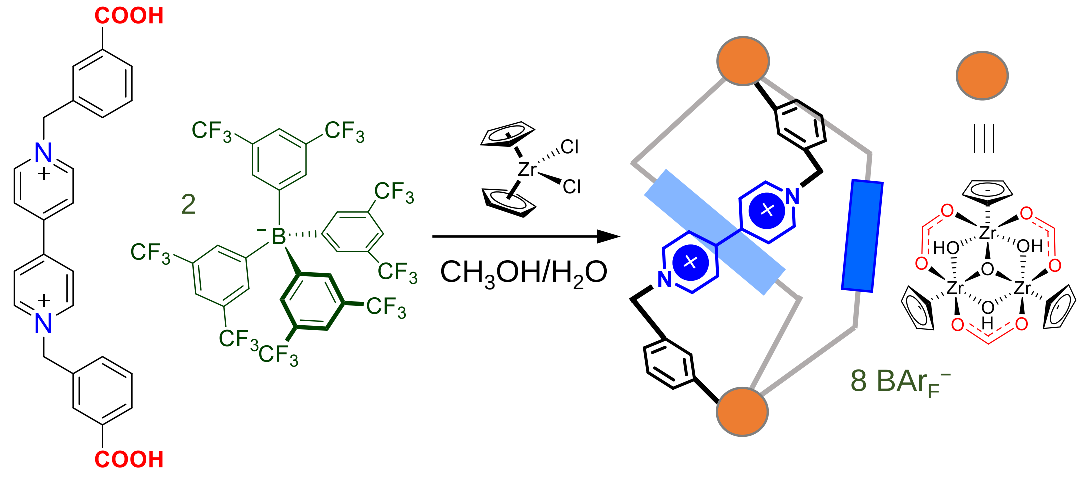

L²-BAr$_F$ **2**-BAr$_F$

Supplementary Fig. 25 | self-assembly of **2**-BAr$_F$.

L²-BAr$_F$ (30 mg, 14 μmol, 1.0 equiv.) and Cp_2ZrCl_2 (8.7 mg, 29 μmol, 2.1 equiv.) were added into a solution mixture containing 2.0 mL CH_3OH and 0.10 mL H_2O. The reaction mixture was stirred at 65 °C overnight. After cooling to room temperature, 3.0 mL H_2O was added and a large amount of white precipitate appeared. The precipitate was collected through centrifugation, thoroughly washed with water (5 mL × 3), and dried under vacuum to obtain **2**-BAr$_F$ (31 mg, 71% yield). **^{1}H NMR** (CD_3CN, 400 MHz, 298 K): δ 8.88 (d, J = 8.0 Hz, 12H), 8.77 (s, 6H), 8.35 (s, 6H), 8.30 (m, 12H), 8.19 (d, J = 10.4 Hz, 6H), 7.72 (m, 96H), 7.46 (t, J = 10.4 Hz, 6H), 7.23 (d, J = 11.2 Hz, 6H), 6.55 (s, 30H), 5.98 (s, 12H) ppm. **^{13}C NMR** (CD_3CN, 100 MHz, 298 K): δ 172.1, 161.8, 150.0, 146.1, 137.3, 134.4, 133.6, 132.2, 131.7, 131.3, 129.8, 128.9, 127.7, 125.6, 123.4, 121.2, 64.2 ppm. **^{19}F NMR** (CD_3CN, 376.4 MHz, 298 K): δ -62.2 ppm. **ESI-MS**: m/z 2220.1 [M+5BAr$_F^-$]$^{3+}$, 1449.6 [M+4BAr$_F^-$]$^{4+}$.

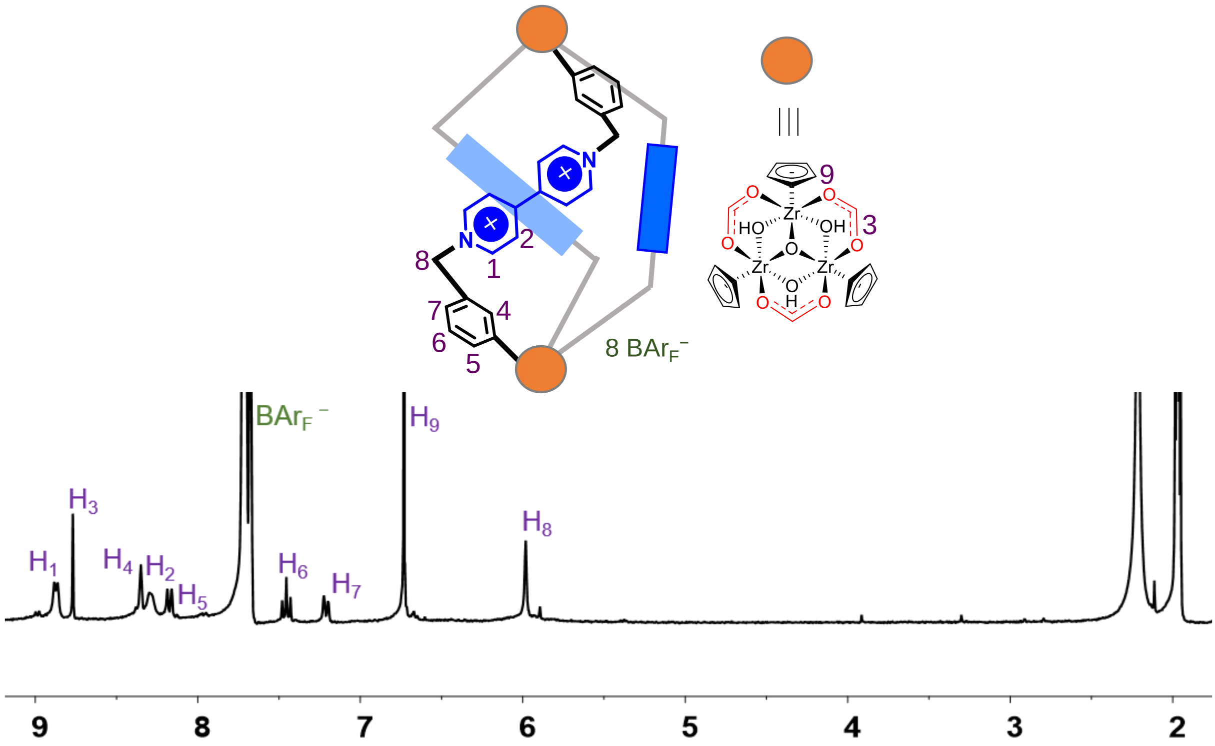

Supplementary Fig. 26 l ^{1}H NMR spectrum (CD_3CN, 400 MHz, 298 K) of **2**-BAr_F.

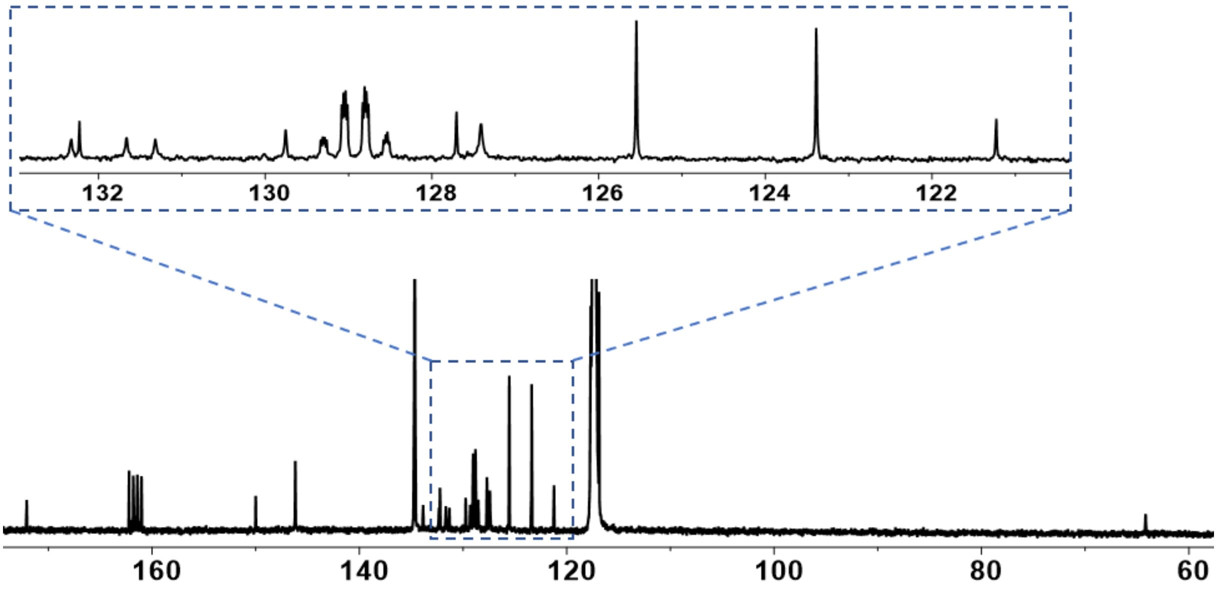

Supplementary Fig. 27 l ^{13}C NMR spectrum (CD_3CN, 100 MHz, 298 K) of **2**-BAr_F.

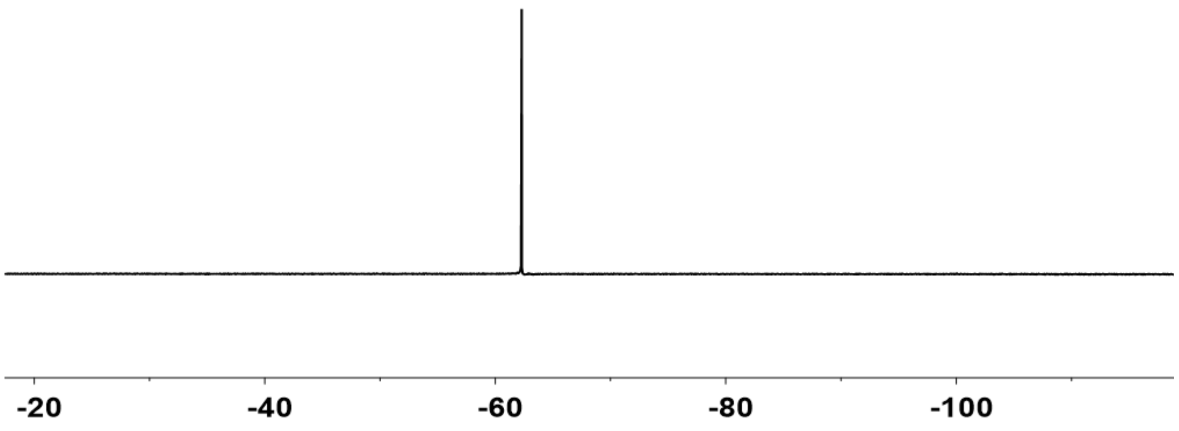

Supplementary Fig. 28 | **^{19}F NMR spectrum (CD_3CN, 376.4 MHz, 298 K) of **2-BAr$_F$.

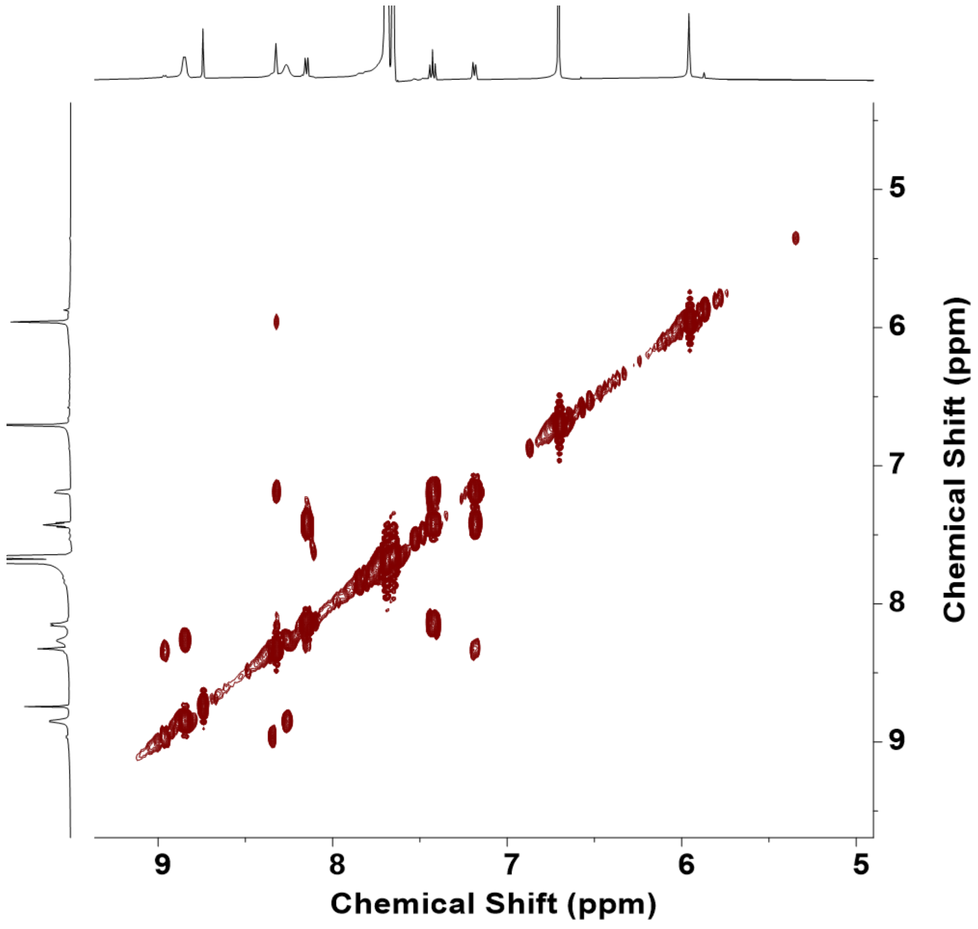

Supplementary Fig. 29 | **^{1}H-^{1}H COSY spectrum (CD_3CN, 400 MHz, 298 K) of **2-BAr$_F$.

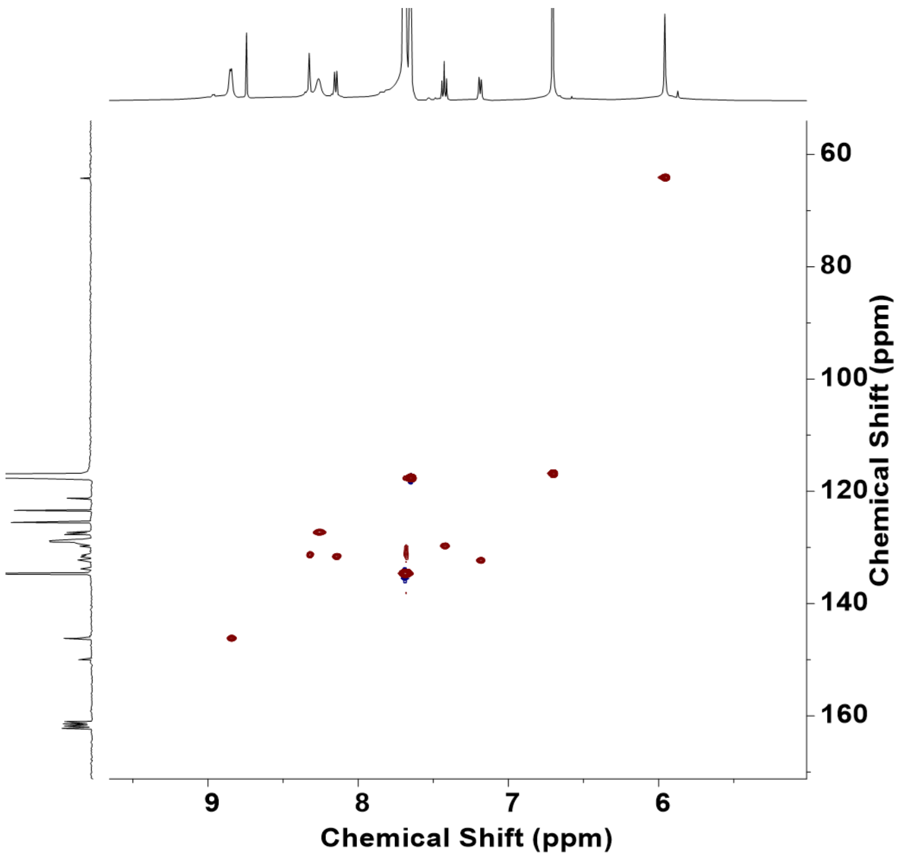

Supplementary Fig. 30 | ^1H-^{13}C HSQC spectrum (CD_3CN, 400 MHz, 298 K) of **2**-BAr_F.

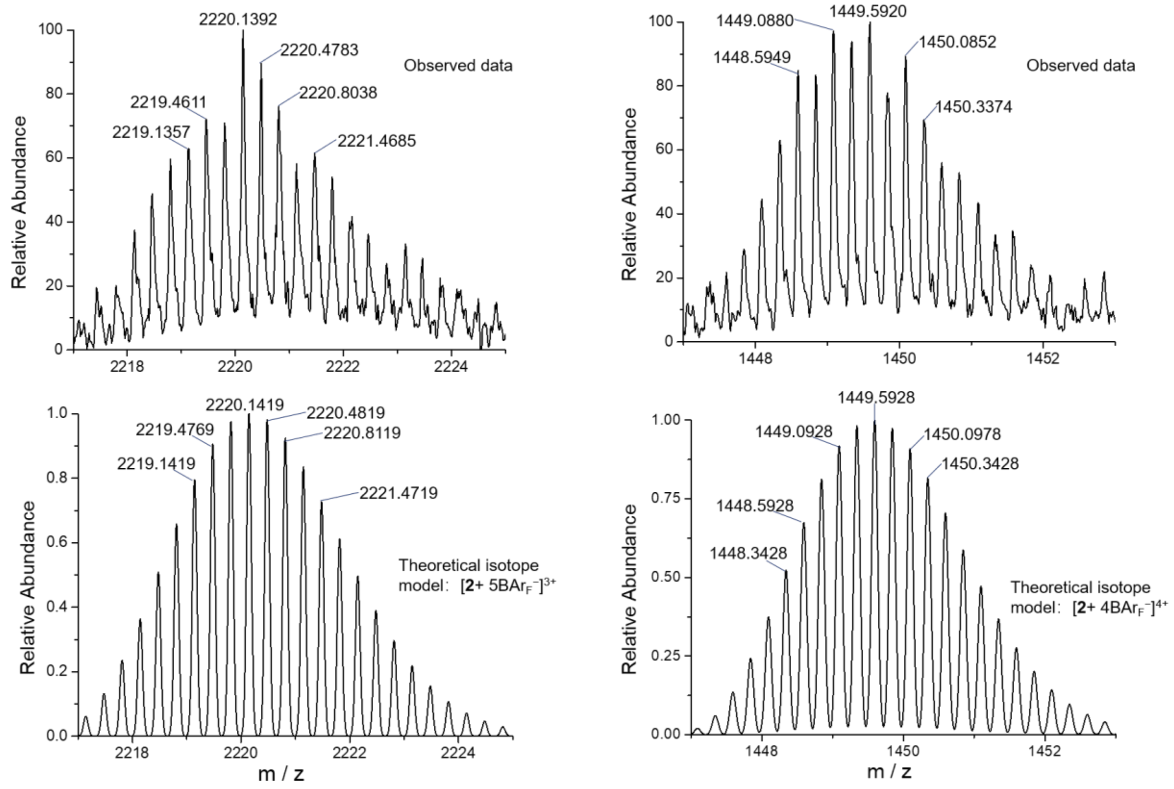

Supplementary Fig. 31 | High-resolution ESI-mass spectrometry analysis of **2**-BAr_F showing the 3+ and 4+ peaks.

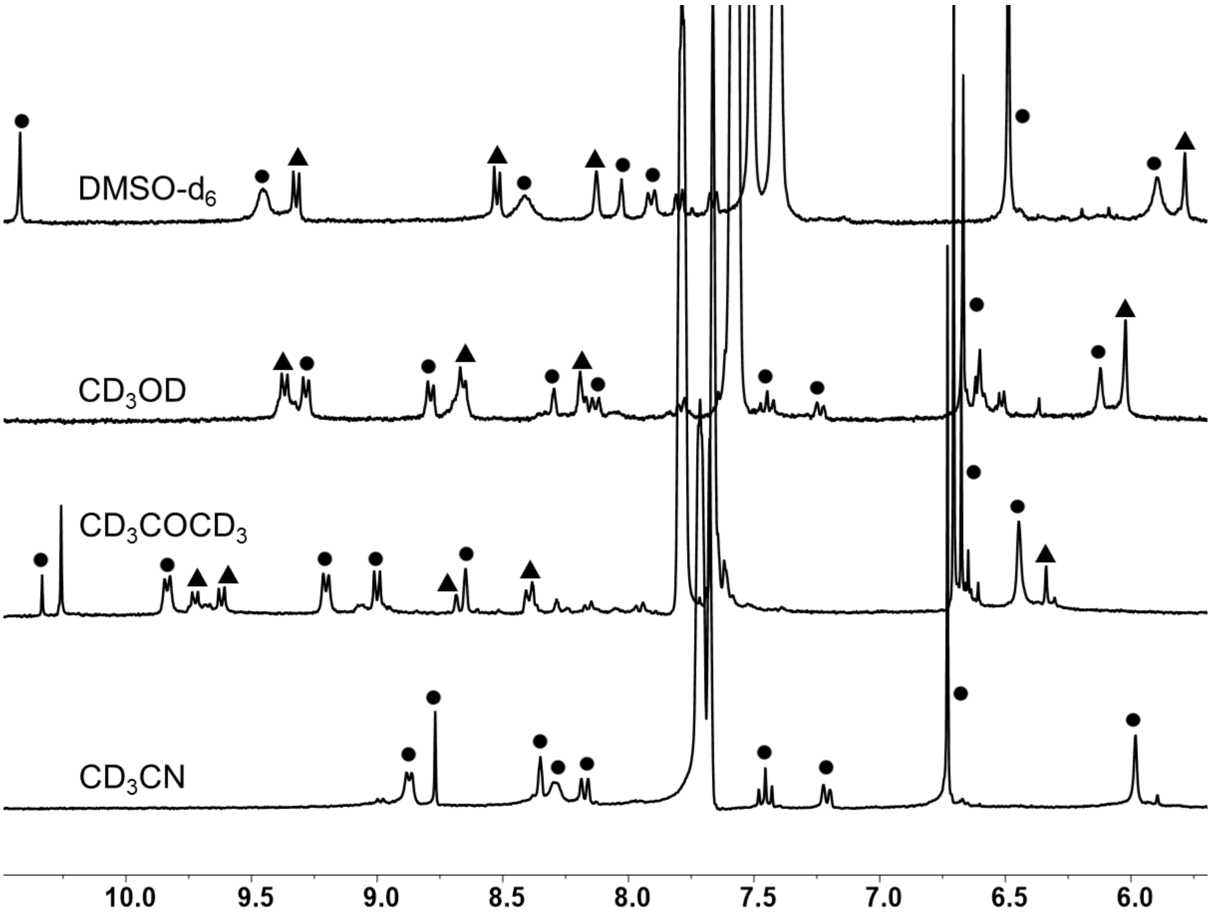

Supplementary Fig. 32 l ^{1}H NMR spectra (400 MHz, 298 K) of **2**-BAr$_F$ in various deuterated solvents. Peaks from **2**-BAr$_F$ are indicated by ● and peaks from **L^2**-BAr$_F$ are indicated by ▲. Results suggest integrity of **2**-BAr$_F$ in CD_3CN, while disassociation of the cage was observed in other solvents.

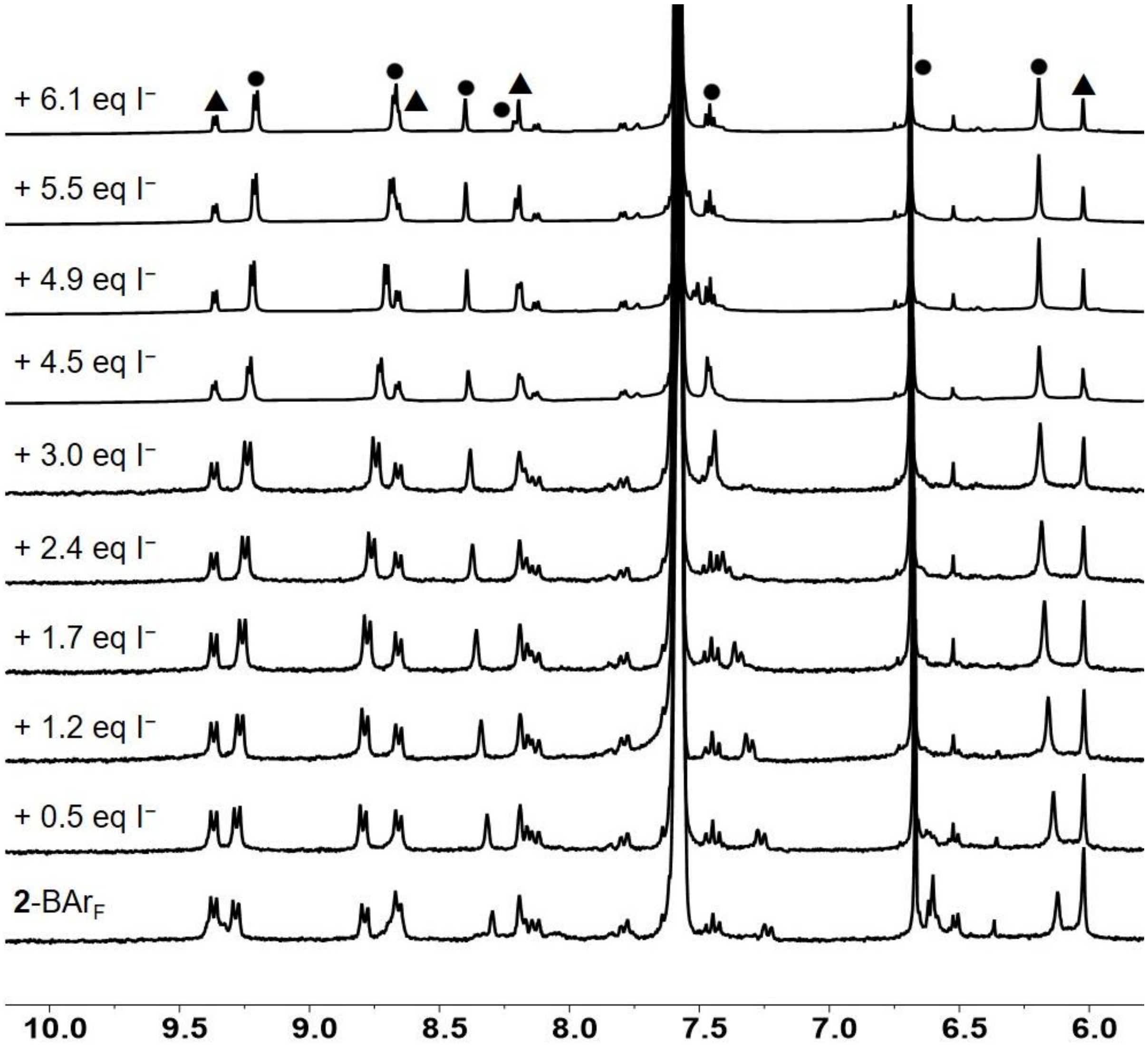

Supplementary Fig. 33 l ^{1}H NMR spectra (CD_3OD, 400 MHz, 298 K) of **2**-BAr_F in the presence of varying equivalents of I^-, showing the template effect of I^- that drives the formation of the cage.

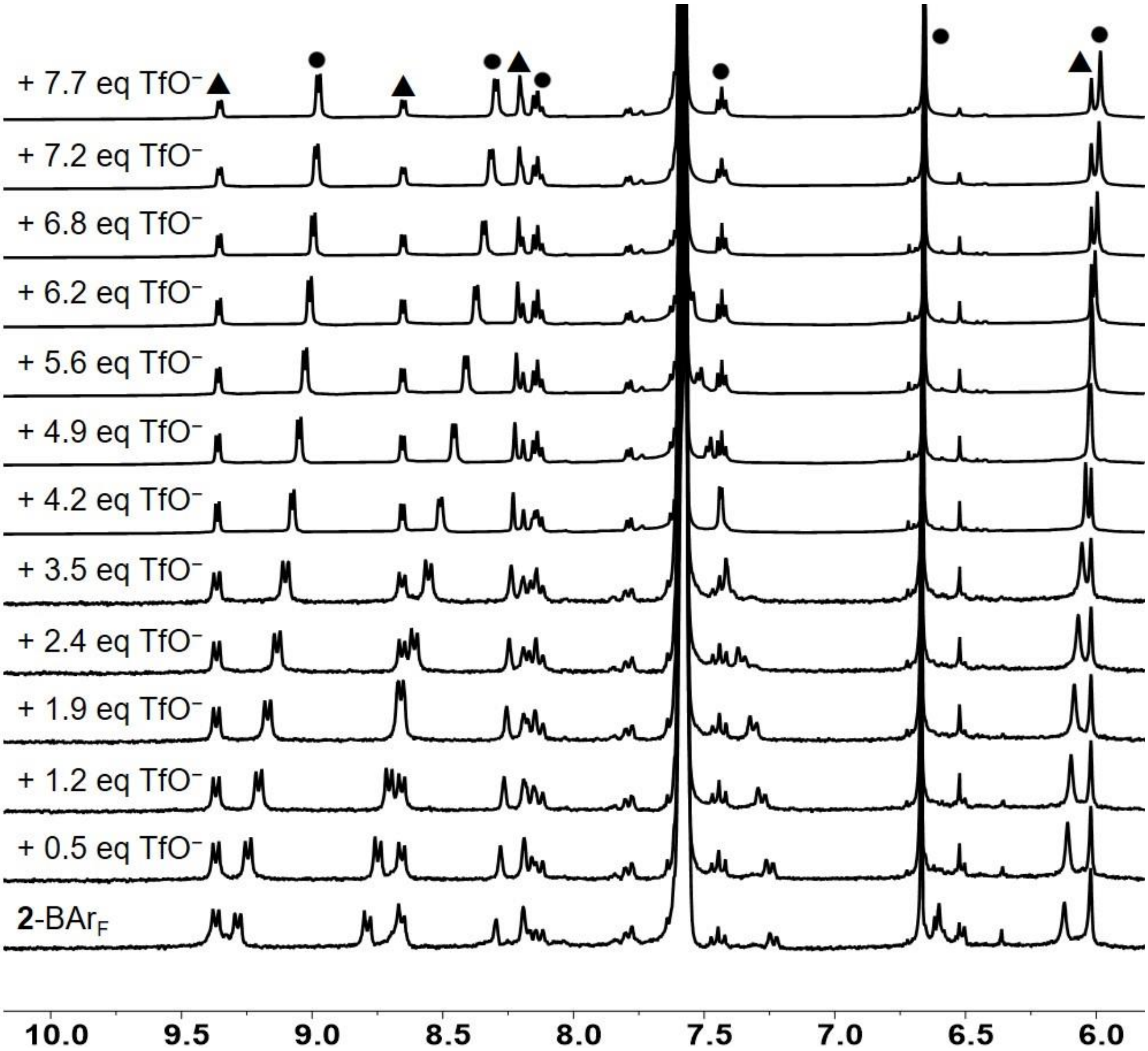

Supplementary Fig. 34 | ^{1}H NMR spectra (CD_3OD, 400 MHz, 298 K) of **2**-BAr_F in the presence of varying equivalents of TfO^-, showing the template effect of TfO^- that drives the formation of the cage.

2.3 Synthesis and characterization of cage 3

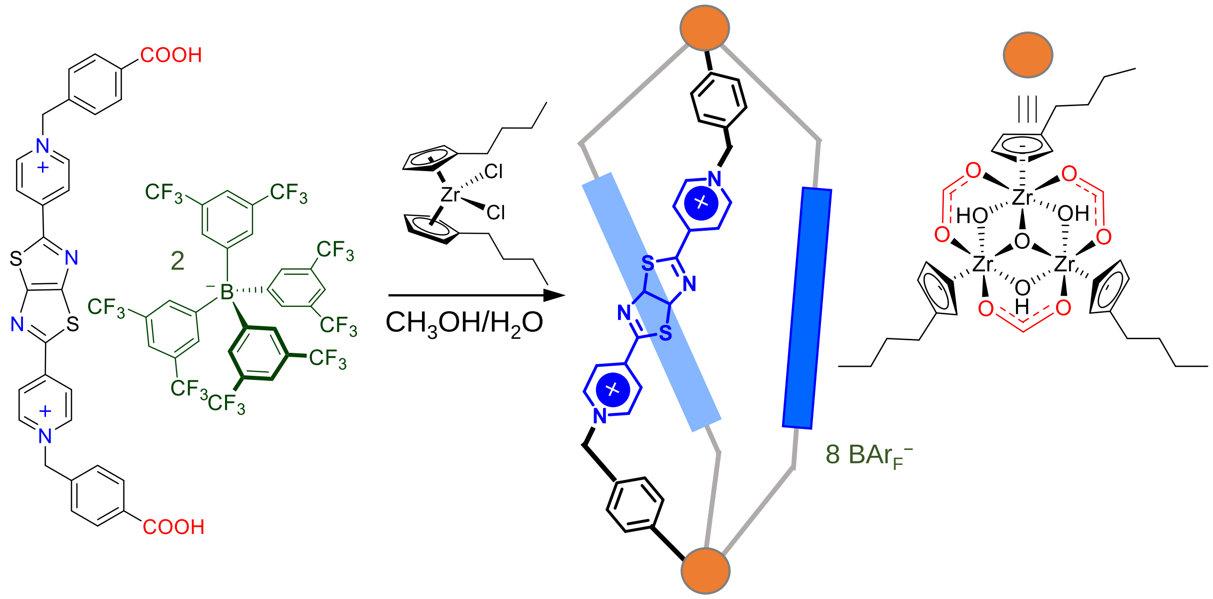

Supplementary Fig. 35 I self-assembly of **3**-BArF.

L3-BArF (20 mg, 8.7 μmol, 1.0 equiv.) and (*n*-BuCp)$_2$ZrCl$_2$ (bis(*n*-butylcyclopentadienyl)zirconium dichloride) (7.3 mg, 18 μmol, 2.1 equiv.) were added into a solution mixture containing 1.5 mL CH_3OH and 60 μL H_2O. The reaction mixture was stirred at 65 °C overnight. After cooling to room temperature, 3.0 mL H_2O was added and a large amount of white precipitate appeared. The precipitate was collected through centrifugation, thoroughly washed with water (5 mL × 3), and dried under vacuum to obtain **3**-BArF (21 mg, 69% yield). **^{1}H NMR** (CD_3OD, 400 MHz, 298 K): δ 8.93 (d, J = 8.1 Hz, 12H), 8.55 (d, J = 8.1 Hz, 12H), 8.04 (d, J = 9.3 Hz, 12H), 7.56-7.60 (m, 108H), 6.52 (m, 12H), 6.35 (m, 12H), 5.80 (s, 12H), 2.75 (m, 12H), 1.62 (m, 12H), 1.34 (m, 12H), 0.88 (m, 18H) ppm. **^{13}C NMR** (CD_3OD, 100 MHz, 298 K): δ 173.7, 167.5, 163.5, 157.8, 147.3, 136.4, 136.2, 135.5, 132.8, 131.7, 131.0, 129.6, 127.5, 126.7, 125.3, 123.2, 119.1, 117.8, 117.3, 64.9, 34.7, 31.3, 24.2, 14.8 ppm. **^{19}F NMR** (CD_3OD, 376.4 MHz, 298 K): δ -62.5 ppm. **ESI-MS**: *m/z* 1638.4 [M+4BArF$^-$]$^{4+}$, 1138.3 [M+3BArF$^-$]$^{5+}$.

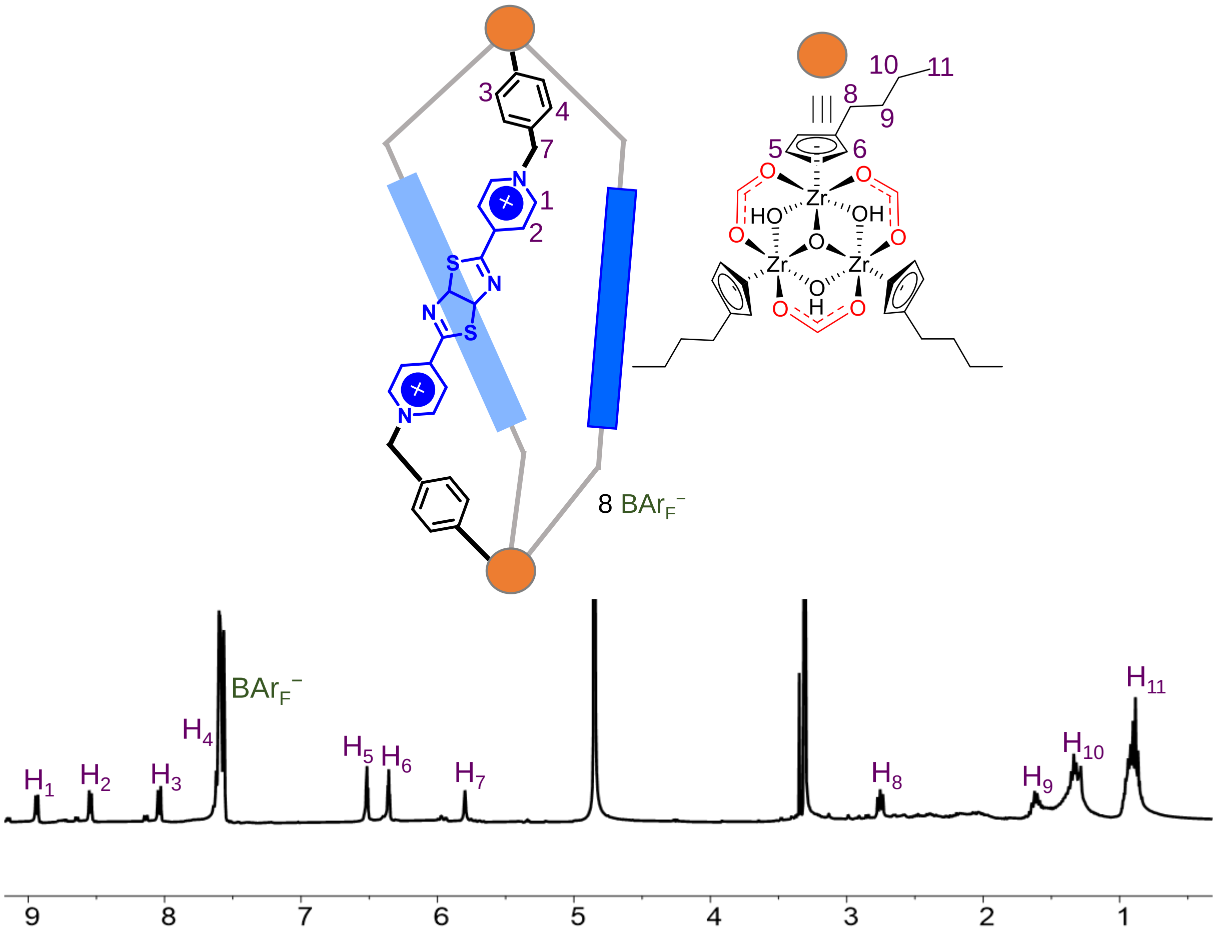

Supplementary Fig. 36 | ^{1}H NMR spectrum (CD_3OD, 400 MHz, 298 K) of **3**-BAr_F.

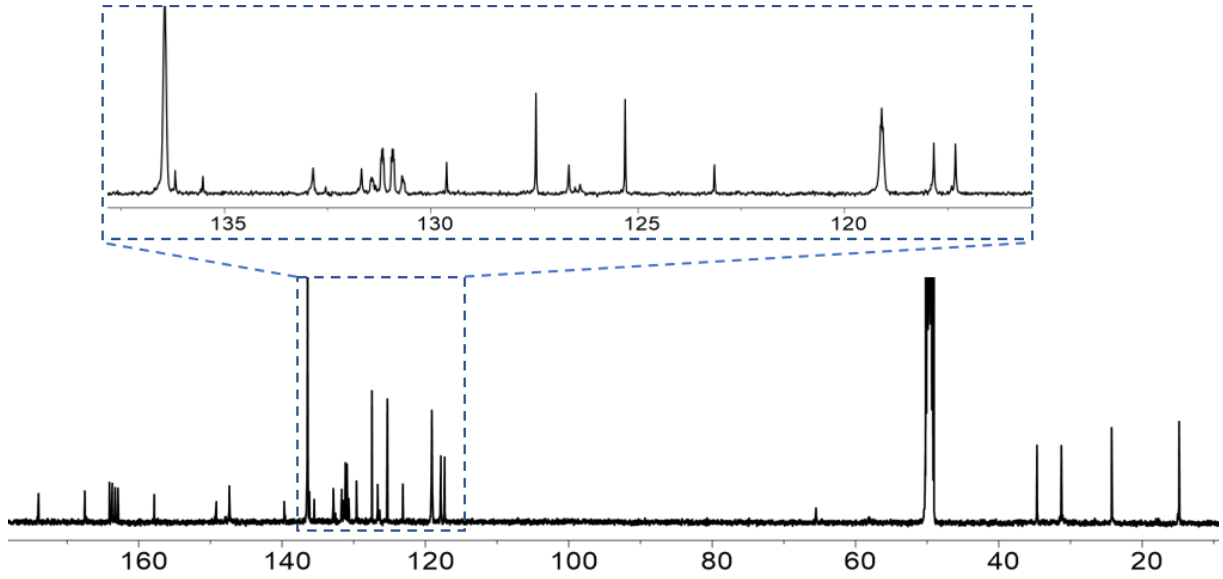

Supplementary Fig. 37 | ^{13}C NMR spectrum (CD_3OD, 100 MHz, 298 K) of **3**-BAr_F.

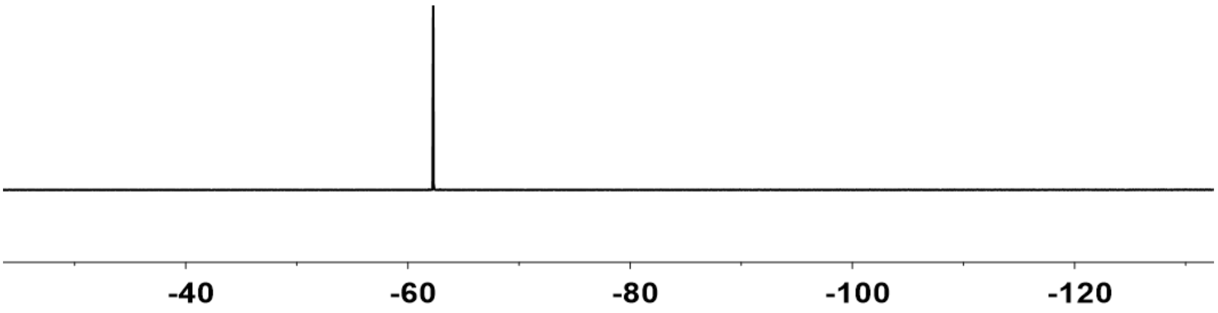

Supplementary Fig. 38 | ^{19}F NMR spectrum (CD_3OD, 376.4 MHz, 298 K) of **3**-BAr_F.

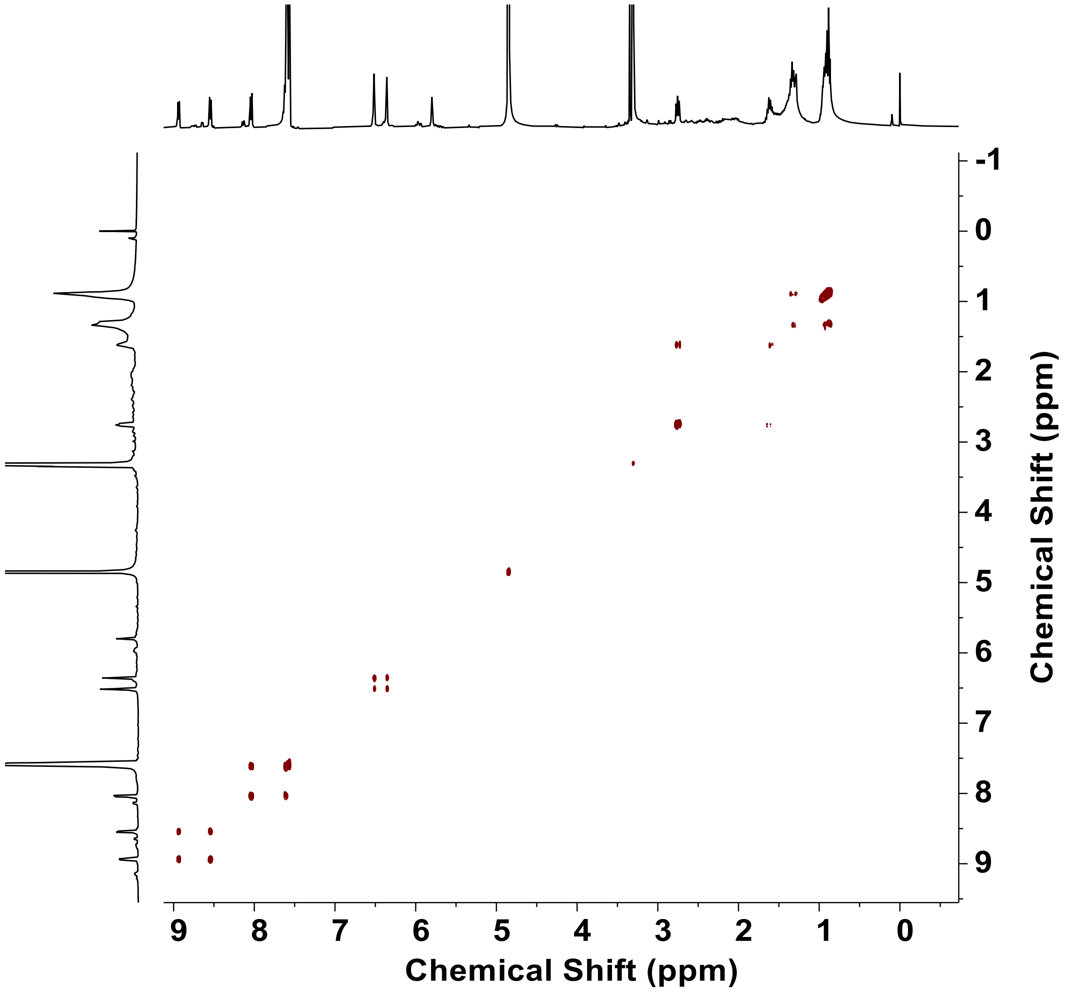

Supplementary Fig. 39 | ^1H-^1H COSY (CD_3OD, 400 MHz, 298 K) spectrum of **3**-BAr_F.

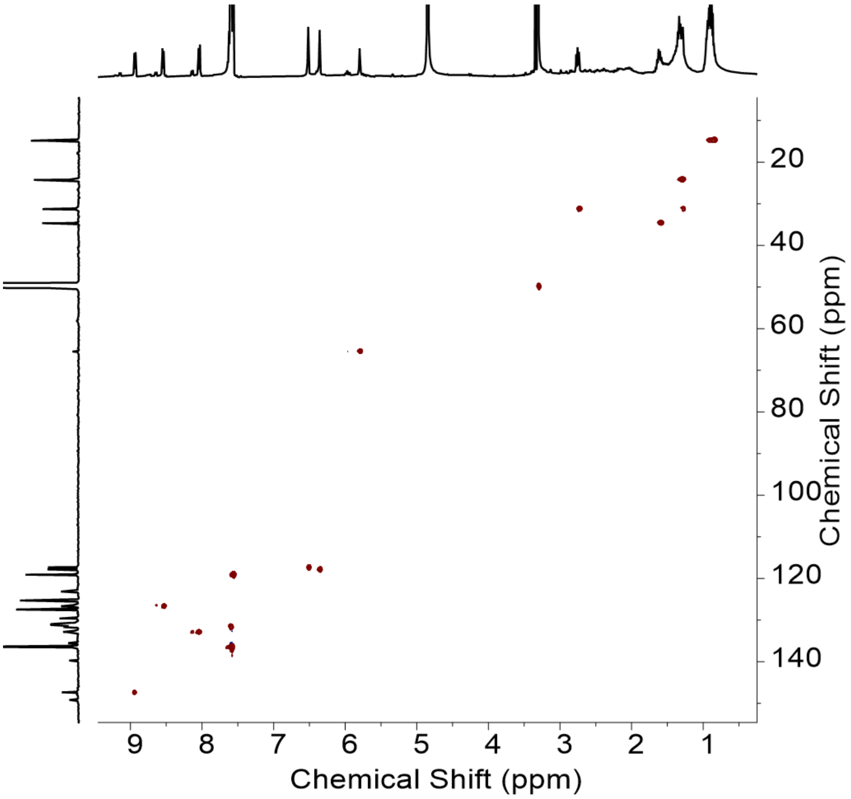

Supplementary Fig. 40 | ^{1}H-^{13}C HSQC (CD$_3$OD, 400 MHz, 298 K) spectrum of **3**-BAr$_F$.

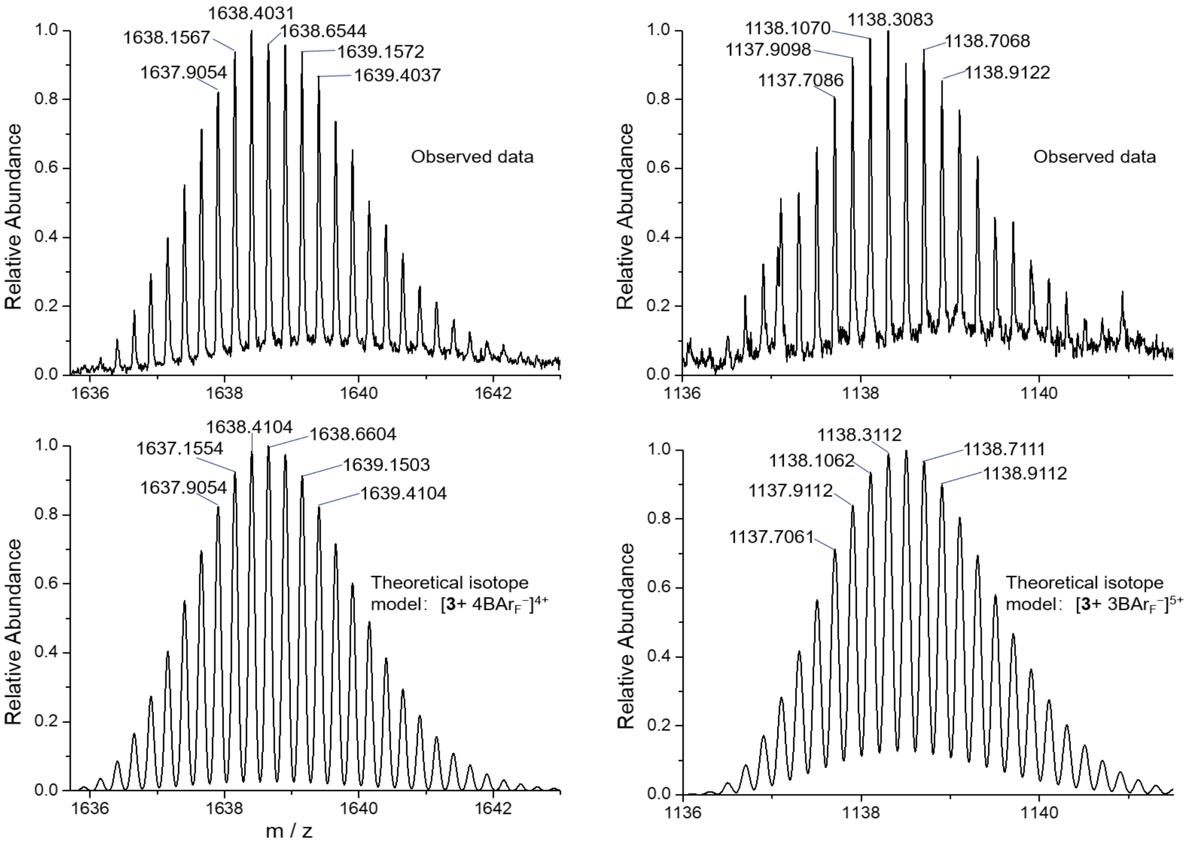

Supplementary Fig. 41 | High-resolution ESI-mass spectrometry analysis of **3**-BAr$_F$ showing the 4+ and 5+ peaks.

2.4 Synthesis and characterization of cage 4

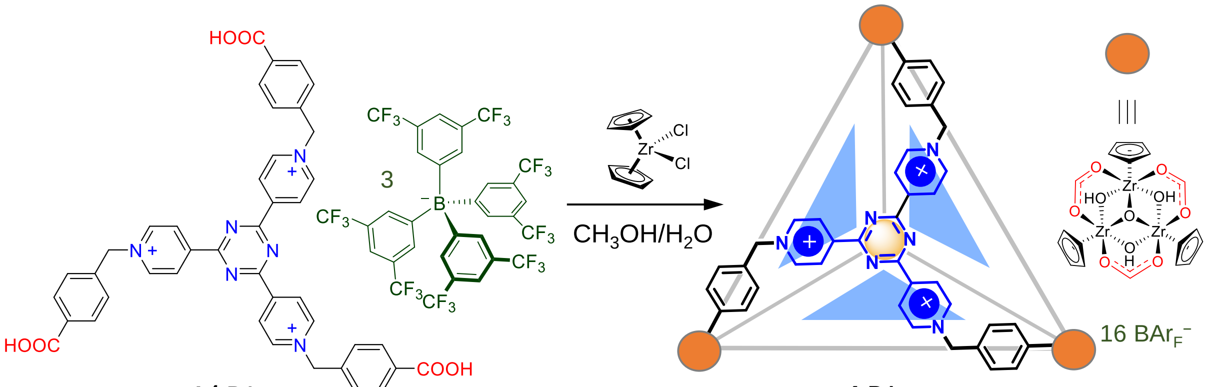

Supplementary Fig. 42 | Self-assembly of **4**-BArF.

L[4]-BArF (20 mg, 6.0 μmol, 1.0 equiv.) and Cp_2ZrCl_2 (5.3 mg, 18 μmol, 3.0 equiv.) were added into a solution mixture containing 3.0 mL CH_3OH and 0.12 mL H_2O. The reaction mixture was stirred at 65 °C overnight. After cooling to room temperature, 3.0 mL H_2O was added and a large amount of white precipitate appeared. The precipitate was collected through centrifugation, thoroughly washed with water (5 mL × 3), and dried under vacuum to obtain **4**-BArF (23 mg, 80% yield). **^{1}H NMR** (CD_3OD, 400 MHz, 298 K): *δ* 9.75 (d, *J* = 8.2 Hz, 24H), 9.47 (d, *J* = 8.2 Hz, 24H), 7.88 (d, *J* = 9.7 Hz, 24H), 7.57-7.63 (m, 246H), 6.52 (s, 58H), 5.90 (s, 24H) ppm. **^{13}C NMR** (CD_3OD, 100 MHz, 298 K): δ 171.5, 163.2, 136.4, 134.5, 132.7, 130.9, 129.9, 129.6, 127.4, 125.2, 123.7, 123.1, 121.2, 119.0, 118.1 ppm. **^{19}F NMR** (CD_3OD, 376.4 MHz, 298 K): δ -62.5 ppm. **ESI-MS**: *m/z* 1740.7 $[M+7Cl^-+4BAr_F^-]^{5+}$, 1582.6 $[M+5Cl^-+5BAr_F^-]^{6+}$, 1351.6 $[M+4Cl^-+5BAr_F^-]^{7+}$.

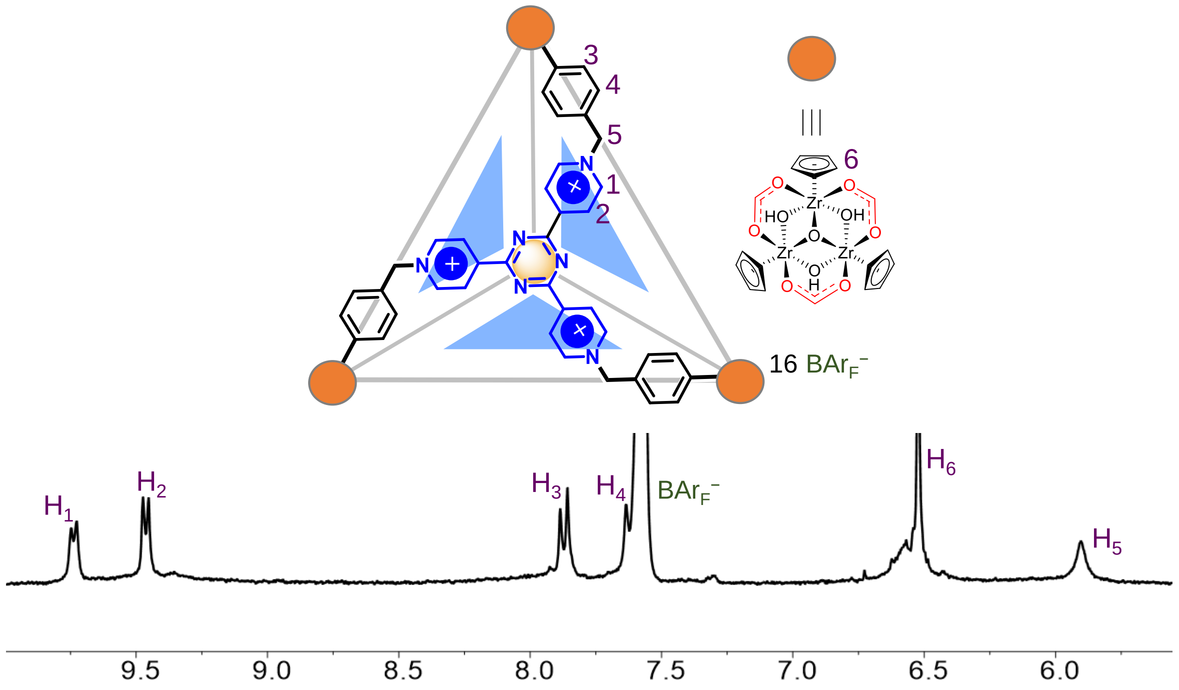

Supplementary Fig. 43 | ^{1}H NMR spectrum (CD_3OD, 400 MHz, 298 K) of **4**-BArF.

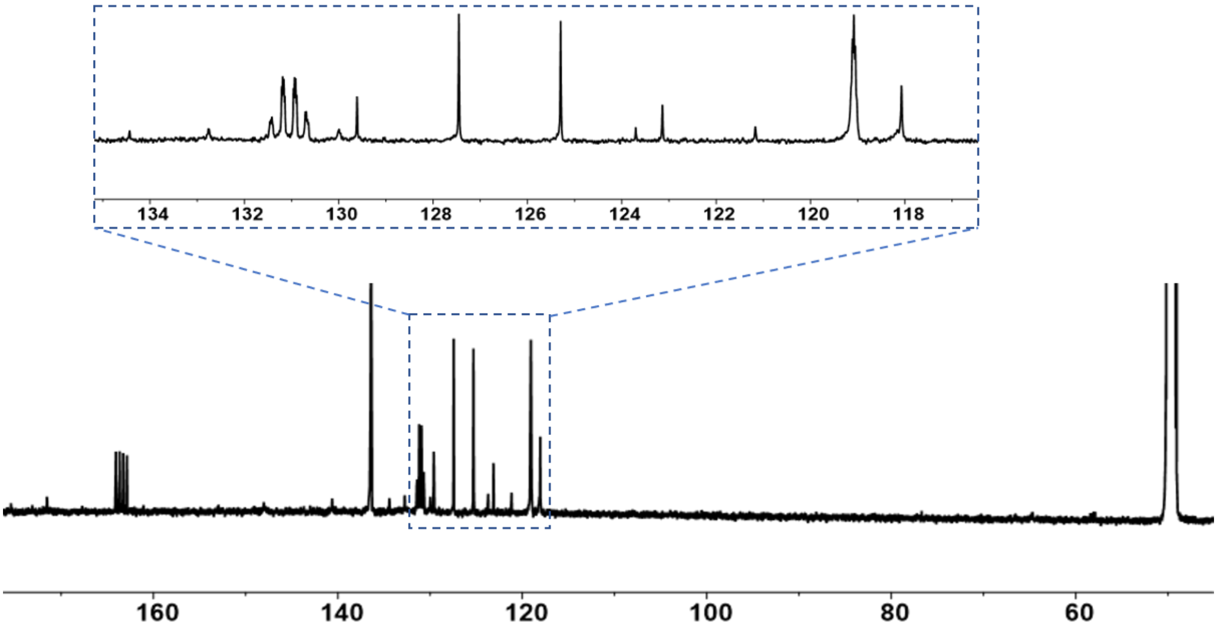

Supplementary Fig. 44 | ^{13}C NMR spectrum (CD_3OD, 100 MHz, 298 K) of **4**-BAr_F.

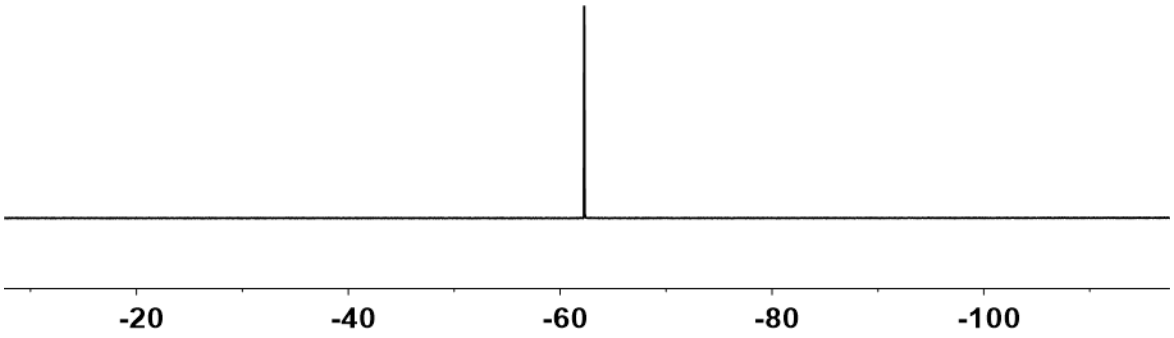

Supplementary Fig. 45 | ^{19}F NMR spectrum (CD_3OD, 376.4 MHz, 298 K) of **4**-BAr_F.

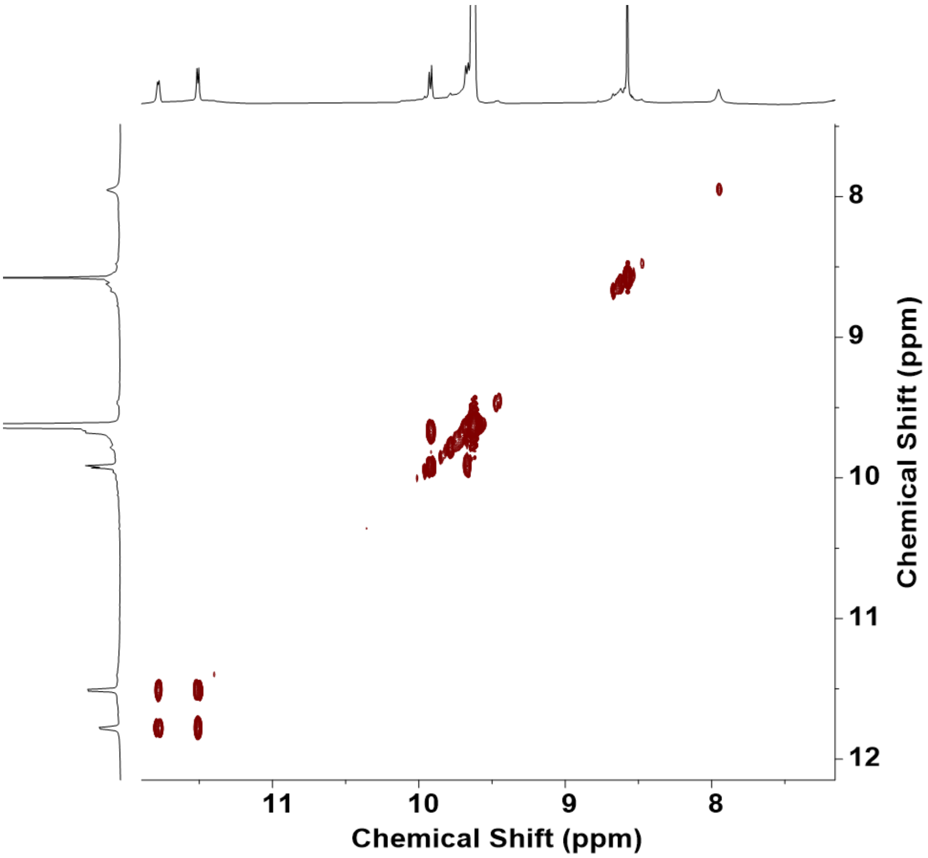

Supplementary Fig. 46 | ^{1}H-^{1}H COSY spectrum (CD_3OD, 400 MHz, 298 K) of **4**-BAr_F.

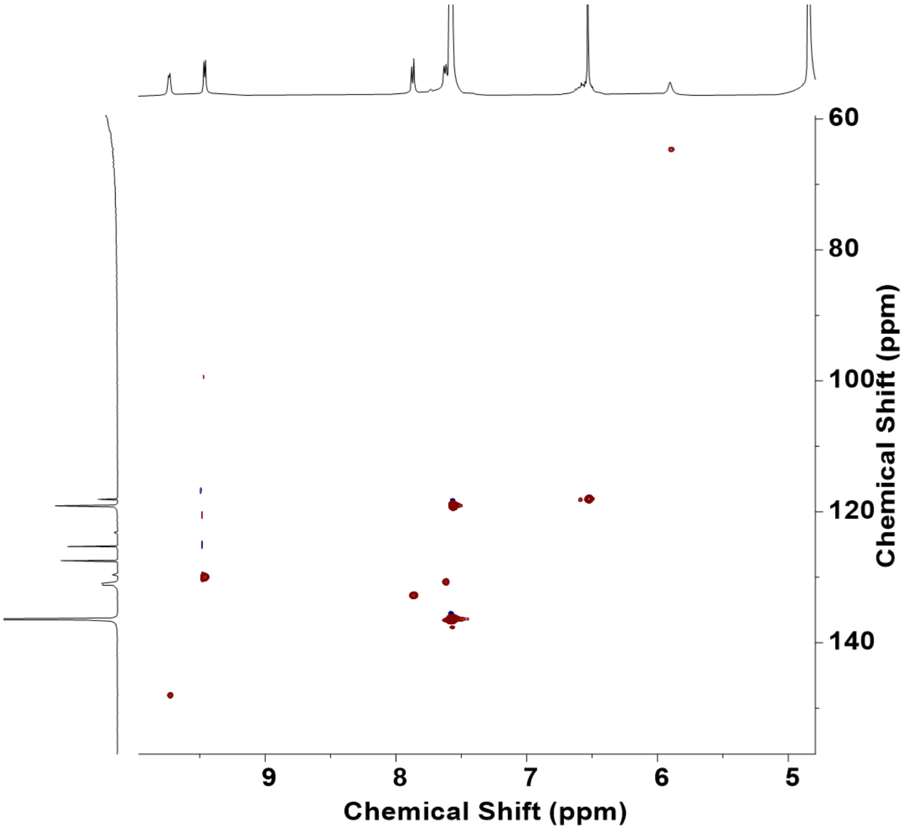

Supplementary Fig. 47 | ^{1}H-^{13}C HSQC spectrum (CD_3OD, 400 MHz, 298 K) of **4**-BAr_F.

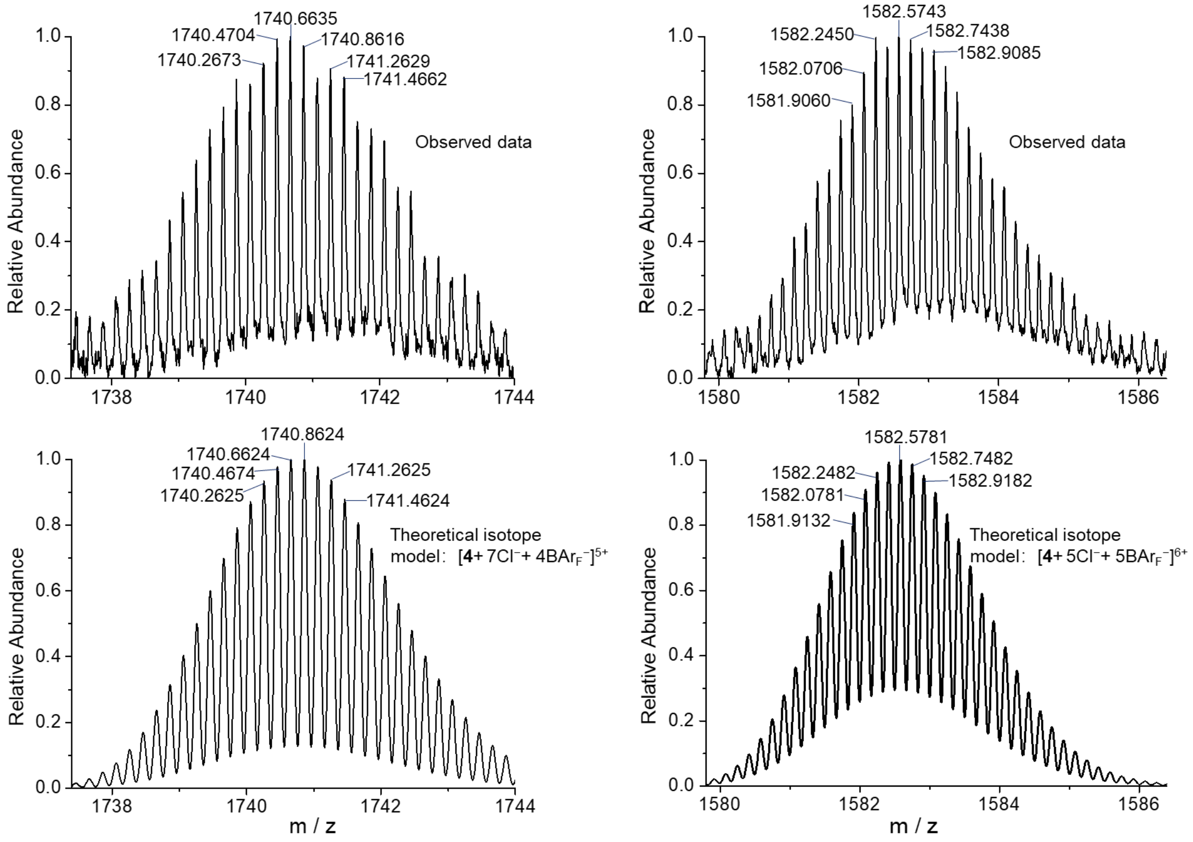

Supplementary Fig. 48 | High-resolution ESI-mass spectrometry analysis of **4**-BAr$_F$ in the presence of TBACl showing the 5+ and 6+ peaks.

3. Supplementary Note 3: X-ray crystallography

Crystal growth: Crystals of **1**-I were grown by vapor diffusion of chloroform into an ethanol solution of **1**-BAr_F containing 20 eq TBAI. Crystals of **1**-SCN and **1**-Br were obtained by dissolving 3.0 mg **1**-BAr_F into 1.5 mL CH_3OH in the presence of 14 eq TBASCN or TBABr, followed by vapor diffusion of diethyl ether at room temperature. Crystals of **3**-SCN were grown by vapor diffusion of chloroform into an ethanol solution of **3**-BAr_F containing an excess of TBASCN (> 30 eq) at room temperature.

Crystals of **1˙**-SCN and **1˙**-Br were obtained by dissolving 3.0 mg **1**-BAr_F into 1.5 mL CH_3OH in the presence of 14 eq TBASCN or TBABr, which were irradiated by Xe lamp in a nitrogen atmosphere for 5.0 min, followed by vapor diffusion of diethyl ether in a nitrogen atmosphere.

Crystallographic methods: Single-crystal X-ray diffraction measurement was measured at 173 K on a Bruker D8 VENTURE diffractometer (Cu-Kα radiation, λ = 1.54184 Å or Mo-Kα radiation, λ = 0.71037 Å) equipped with a graphite monochromator and a CCD area detector. The structure was solved using direct methods and refined to convergence by full matrix least-squares on F^2 employing the SHELXTL-2014 program.[4] The H atoms attached to carbons were added geometrically and refined isotropically with the riding model. The contribution from solvent molecules and unlocated anions (see below) were removed using the "SQUEEZE" protocol of the PLATON program.[5]

1-I: [I⊂**1**]·7I [+ solvent], CCDC 2356397

Formula $C_{108}H_{96}N_6O_{20}I_8Zr_6$, *M* 3360.56, Monoclinic, space group *P2₁/c*(#14), *a* 16.2704(6), *b* 31.7131(2), *c* 32.7811(3) Å, *β* 98.0280(20)°, *V* 16784.8(1) Å^3, D_c 1.333 g cm^{-3}, *Z* 4, crystal size 0.28 by 0.23 by 0.19 mm, colour purple, block, temperature 173(2) K, *λ*(Cu-Kα) 1.54178 Å, $2\theta_{max}$ 126.00, $R1(F)$ 0.1406, $wR2(F^2)$ 0.3824, GoF(all) 1.272, μ(mm^{-1}) 14.909, *F*(000) 6440.

1-Br: [Br⊂**1**]·7Br [+ solvent], CCDC 2356396

Formula $C_{108}H_{96}N_6O_{20}Br_8Zr_6$, *M* 2984.55, Monoclinic, space group $P2_1$(#4), *a* 22.6406(5), *b* 16.4026(3), *c* 24.1789(6) Å, *β* 115.7280(1)°, *V* 8089.0(3) Å^3, D_c 1.225 g cm^{-3}, *Z* 2, crystal size 0.36 by 0.31 by 0.22 mm, colour yellow, block, temperature 173(2) K, *λ*(Cu-Kα) 1.54178 Å, $2\theta_{max}$ 137.81, $R1(F)$ 0.0807, $wR2(F^2)$ 0.2391, GoF(all) 0.999, μ(mm^{-1}) 5.736, *F*(000) 2932.

1-SCN: 1·8SCN [+ solvent], CCDC 2356395

Formula $C_{116}H_{96}N_{14}O_{20}S_8Zr_6$, *M* 2809.95, Monoclinic, space group $P2_1/c$(#14), *a* 22.5191(5), *b* 16.2430(4), *c* 43.8728(1) Å, *β* 98.7080(1)°, *V* 15862.7(7) Å^3, D_c 1.079 g cm^{-3}, *Z* 4, crystal size 0.32 by 0.24 by 0.20 mm, colour pale yellow, block, temperature 173(2) K, *λ*(Cu-Kα) 1.54178 Å, $2\theta_{max}$ 136.88, $R1(F)$ 0.0499, $wR2(F^2)$ 0.1408, GoF(all) 1.061, μ(mm^{-1}) 4.025, *F*(000) 5208.

Note: Ion chromatography revealed that there are eight SCN^- per cage for **1**-SCN, which is as expected for the charge balance of $\mathbf{1}^{8+}$. Only four SCN^-anions (all outside the cage) per cage were located by X-ray crystallography, and the remaining four SCN^- anions could not be located perhaps due to the high disorder of the anions and the limited quality of the data.

3-SCN: 3·8SCN [+ solvent], CCDC 2356398

Formula $C_{152}H_{144}N_{20}O_{20}S_{14}Zr_6$, *M* 3567.13, Monoclinic, space group *P2₁/c(#14)*, *a* 26.4846(7), *b* 26.4962(7), *c* 31.5550(8) Å, *β* 109.7510(10)°, *V* 20840.7(9) Å^3, D_c 1.018 g cm^{-3}, *Z* 4, crystal size 0.32 by 0.25 by 0.16 mm, colour yellow, block, temperature 173(2) K, *λ*(Cu-Kα) 1.54178 Å, $2\theta_{max}$ 136.48, *R*1(*F*) 0.0660, *wR*2(F^2) 0.2242, GoF(all) 1.046, *μ*(mm^{-1}) 3.608, *F*(000) 6508.

Note: Only three SCN^- anions (all outside the cage) per cage were located by X-ray crystallography, and the remaining five SCN^- anions could not be located perhaps due to the high disorder of the anions and the limited quality of the data.

1˙-Br: [Br⊂**1˙**]·6Br [+ solvent], CCDC 2359410

Formula $C_{108}H_{96}N_6O_{20}Br_7Zr_6$, *M* 2904.53, Monoclinic, space group *P2₁/n(#14)*, *a* 20.1545(15), *b* 27.193(2), *c* 28.014(2) Å, *β* 94.328(4)°, *V* 15310(2) Å^3, D_c 1.260 g cm^{-3}, *Z* 4, crystal size 0.33 by 0.25 by 0.20 mm, colour blue, block, temperature 173(2) K, *λ*(Cu-Kα) 1.54178 Å, $2\theta_{max}$ 128.38, *R*1(*F*) 0.1021, *wR*2(F^2) 0.2946, GoF(all) 1.273, *μ*(mm^{-1}) 5.782, *F*(000) 5724.0.

1˙-SCN: 1˙·6SCN [+ solvent], CCDC 2357282

Formula $C_{114}H_{96}N_{12}O_{20}S_6Zr_6$, *M* 2693.79, Orthorhombic, space group *Aba2*(#41), *a* 31.481(6), *b* 24.112 (5), *c* 21.777(4) Å, *V* 16530(6) Å^3, D_c 0.989 g cm^{-3}, *Z* 4, crystal size 0.29 by 0.21 by 0.14 mm, colour blue, block, temperature 293(2) K, *λ*(Mo-Kα) 0.71037 Å, $2\theta_{max}$ 50.82, *R*1(*F*) 0.1089, *wR*2(F^2) 0.2988, GoF(all) 1.247, *μ*(mm^{-1}) 0.436, *F*(000) 4976.

Note: Ion chromatography revealed that there are six SCN^- per cage for **1˙**-SCN. Only two SCN^-anions (both outside the cage) per cage were located by X-ray crystallography, and the remaining four SCN^- anions could not be located perhaps due to the high disorder of the anions and the limited quality of the data.

Explanation of the B-level alerts of CheckCif:

Alert level B of 1-I:

PLAT084_ALERT_3_B High wR2 Value (i.e. > 0.25) .... 0.38 Report

PLAT241_ALERT_2_B High ’MainMol’ Ueq as Compared to Neighbors of C9 Check

PLAT241_ALERT_2_B High ’MainMol’ Ueq as Compared to Neighbors of C15 Check

PLAT241_ALERT_2_B High ’MainMol’ Ueq as Compared to Neighbors of C16 Check

PLAT241_ALERT_2_B High ’MainMol’ Ueq as Compared to Neighbors of C24 Check

PLAT241_ALERT_2_B High ’MainMol’ Ueq as Compared to Neighbors of C26 Check

PLAT241_ALERT_2_B High ’MainMol’ Ueq as Compared to Neighbors of C89 Check

PLAT241_ALERT_2_B High ’MainMol’ Ueq as Compared to Neighbors of C90 Check

PLAT242_ALERT_2_B Low ’MainMol’ Ueq as Compared to Neighbors of N1 Check

PLAT242_ALERT_2_B Low 'MainMol' Ueq as Compared to Neighbors of N2 Check

PLAT242_ALERT_2_B Low 'MainMol' Ueq as Compared to Neighbors of C11 Check

PLAT242_ALERT_2_B Low 'MainMol' Ueq as Compared to Neighbors of C12 Check

PLAT260_ALERT_2_B Large Average Ueq of Residue Including I4 0.312 Check

PLAT260_ALERT_2_B Large Average Ueq of Residue Including I7 0.415 Check

PLAT260_ALERT_2_B Large Average Ueq of Residue Including I8 0.312 Check

PLAT260_ALERT_2_B Large Average Ueq of Residue Including I4' 0.312 Check

PLAT342_ALERT_3_B Low Bond Precision on C-C Bonds .... 0.0311 Ang.

Explanation: The above alerts arise from some degree of disorder of the benzene, pyridyl, cyclopentadienyl rings and I^- containing the above atoms, but the disorder could not be satisfactorily modeled owing to the limited quality of the reflection dataset.

PLAT420_ALERT_2_B D-H Bond Without Acceptor O13--H13B. Please Check

PLAT420_ALERT_2_B D-H Bond Without Acceptor O14--H14B. Please Check

PLAT420_ALERT_2_B D-H Bond Without Acceptor O18--H18B. Please Check

PLAT420_ALERT_2_B D-H Bond Without Acceptor O19--H19B. Please Check

Explanation: These O-H bonds are both hydroxyl bridges and are expected to form hydrogen-bonds with a water molecule of crystallization. However, owing to the limited quality of the reflection dataset, the heavily disordered solvent molecules in the structure could not be satisfactorily modeled, and the final refinements were performed after removing the scattering of disordered solvent molecules using the PLATON/SQUEEZE tool.

Alert level B of 1-Br:

PLAT342_ALERT_3_B Low Bond Precision on C-C Bonds .... 0.02913 Ang.

Explanation: This is due to poor diffraction of the crystal.

PLAT420_ALERT_2_B D-H Bond Without Acceptor O13--H13B. Please Check

PLAT420_ALERT_2_B D-H Bond Without Acceptor O19--H19C. Please Check

Explanation: O13--H13B and O19--H19C are both hydroxyl bridges and are expected to form hydrogen-bonds with a water molecule of crystallization. However, owing to the limited quality of the reflection dataset, the heavily disordered solvent molecules in the structure could not be satisfactorily modeled, and the final refinements were performed after removing the scattering of disordered solvent molecules using the PLATON/SQUEEZE tool.

Alert level B of 1-SCN:

PLAT420_ALERT_2_B D-H Bond Without Acceptor O14--H14. Please Check

PLAT420_ALERT_2_B D-H Bond Without Acceptor O18--H18. Please Check

Explanation: O14--H14 and O18--H18 are both hydroxyl bridges and are expected to form hydrogen-bonds with a water molecule of crystallization. However, owing to the limited quality of the reflection dataset, the heavily disordered solvent molecules in the structure could not be satisfactorily modeled, and the final refinements were performed after removing the scattering of disordered solvent molecules using the PLATON/SQUEEZE tool.

Alert level B of 3-SCN:

PLAT220_ALERT_2_B NonSolvent Resd 1 C Ueq(max)/Ueq(min) Range 7.3 Ratio

PLAT241_ALERT_2_B High 'MainMol' Ueq as Compared to Neighbors of S5 Check

PLAT241_ALERT_2_B High 'MainMol' Ueq as Compared to Neighbors of C103 Check

PLAT241_ALERT_2_B High 'MainMol' Ueq as Compared to Neighbors of C113 Check

PLAT241_ALERT_2_B High 'MainMol' Ueq as Compared to Neighbors of C120 Check

PLAT241_ALERT_2_B High 'MainMol' Ueq as Compared to Neighbors of C130 Check

PLAT241_ALERT_2_B High 'MainMol' Ueq as Compared to Neighbors of C138 Check

PLAT242_ALERT_2_B Low 'MainMol' Ueq as Compared to Neighbors of C121 Check

PLAT242_ALERT_2_B Low 'MainMol' Ueq as Compared to Neighbors of C137 Check

Explanation: The above alerts arise from some degree of disorder of the thiazole, cyclopentadienyl rings and N-butyl chains containing the above atoms, but the disorder could not be satisfactorily modeled owing to the limited quality of the reflection dataset.

PLAT420_ALERT_2_B D-H Bond Without Acceptor O12--H12P. Please Check

PLAT420_ALERT_2_B D-H Bond Without Acceptor O16--H16A. Please Check

PLAT420_ALERT_2_B D-H Bond Without Acceptor O17--H17A. Please Check

Explanation: O12--H12P, O16--H16A and O17--H17A are both hydroxyl bridges and are expected to form hydrogen-bonds with a water molecule of crystallization. However, owing to the limited quality of the reflection dataset, the heavily disordered solvent molecules in the structure could not be satisfactorily modeled, and the final refinements were performed after removing the scattering of disordered solvent molecules using the PLATON/SQUEEZE tool.

Alert level B of $1^{\bullet}$-Br:

PLAT241_ALERT_2_B High 'MainMol' Ueq as Compared to Neighbors of C30 Check

PLAT241_ALERT_2_B High 'MainMol' Ueq as Compared to Neighbors of C43 Check

PLAT241_ALERT_2_B High 'MainMol' Ueq as Compared to Neighbors of C45 Check

PLAT241_ALERT_2_B High 'MainMol' Ueq as Compared to Neighbors of C73 Check

PLAT242_ALERT_2_B Low 'MainMol' Ueq as Compared to Neighbors of N4 Check

PLAT342_ALERT_3_B Low Bond Precision on C-C Bonds .... 0.02403 Ang.

Explanation: The above alerts arise from some degree of disorder of the benzene and pyridyl rings containing the above atoms, but the disorder could not be satisfactorily modeled owing to the limited quality of the reflection dataset.

PLAT420_ALERT_2_B D-H Bond Without Acceptor O17--H17B. Please Check

Explanation: O17--H17B is a hydroxyl bridge and are expected to form hydrogen-bonds with a water molecule of crystallization. However, owing to the limited quality of the reflection dataset, the heavily disordered solvent molecules in the structure could not be satisfactorily modeled, and the final refinements were performed after removing the scattering of disordered solvent molecules using the PLATON/SQUEEZE tool.

Alert level B of $1^{\bullet}$-SCN:

PLAT241_ALERT_2_B High 'MainMol' Ueq as Compared to Neighbors of C31 Check

PLAT241_ALERT_2_B High 'MainMol' Ueq as Compared to Neighbors of C32 Check

PLAT241_ALERT_2_B High 'MainMol' Ueq as Compared to Neighbors of C47 Check

PLAT241_ALERT_2_B High 'MainMol' Ueq as Compared to Neighbors of C53 Check

PLAT242_ALERT_2_B Low 'MainMol' Ueq as Compared to Neighbors of C30 Check

PLAT342_ALERT_3_B Low Bond Precision on C-C Bonds .... 0.02709 Ang.

Explanation: The above alerts arise from some degree of disorder of the benzene and pyridyl rings containing the above atoms, but the disorder could not be satisfactorily modeled owing to the limited quality of the reflection dataset.

PLAT420_ALERT_2_B D-H Bond Without Acceptor O6--H66A. Please Check

PLAT420_ALERT_2_B D-H Bond Without Acceptor O7--H77A. Please Check

Explanation: O6–H66A and O7–H77A are both hydroxyl bridges and are expected to form hydrogen-bonds with a water molecule of crystallization. However, owing to the limited quality of the reflection dataset, the heavily disordered solvent molecules in the structure could not be satisfactorily modeled, and the final refinements were performed after removing the scattering of disordered solvent molecules using the PLATON/SQUEEZE tool.

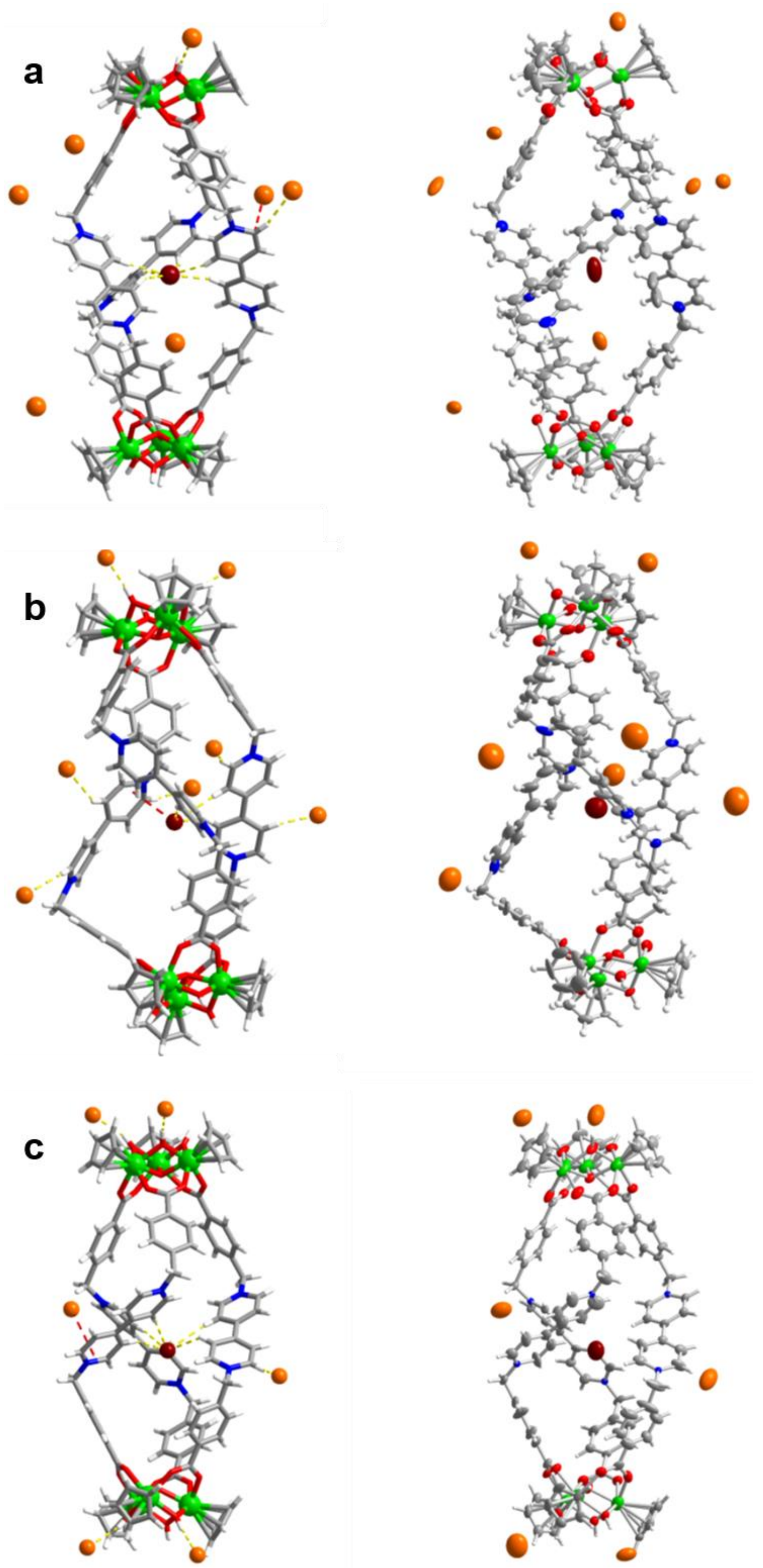

Supplementary Fig. 49 l Views (Ball-Stick and ORTEP (ellipsoids with 50% probability)) showing the cages and anions in **1**-I (a) (CCDC 2356397), **1**-Br (b) (CCDC 2356396) and **1**$^{\bullet}$-Br (c) (CCDC 2359410). Dark red and orange balls represent the anions located inside and outside the cage, respectively. The dashed lines represent the interactions of the anions with the cages.

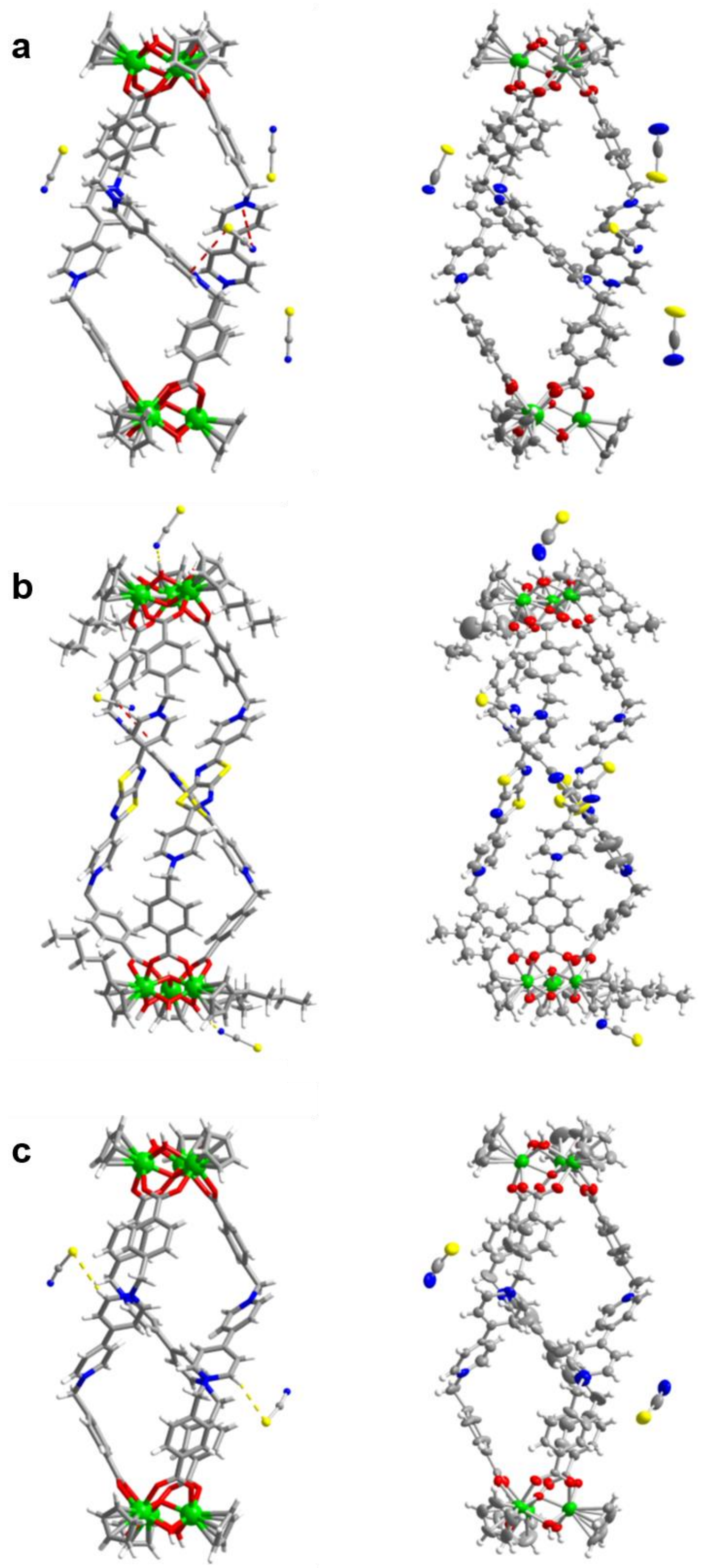

Supplementary Fig. 50 l Views (Ball-Stick and and ORTEP (ellipsoids with 50% probability)) showing the cages and anions in **1**-SCN (a) (CCDC 2356395), **3**-SCN (b) (CCDC 2356398) and **1**$^{\bullet}$-SCN (c) (CCDC 2357282). All anions are outside the cages. The dashed lines represent the interactions of the anions with the cages.

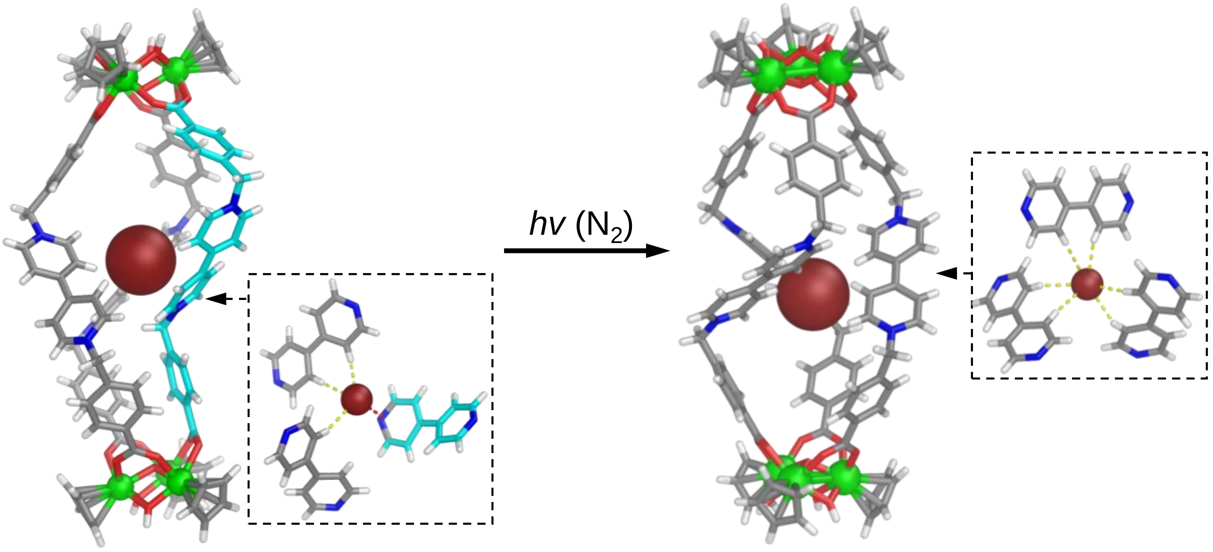

Supplementary Fig. 51 | X-ray crystallographic structures of **1**-Br and **1**•-Br. Yellow and red dotted lines represent CH···X⁻ and anion-π interactions, respectively.

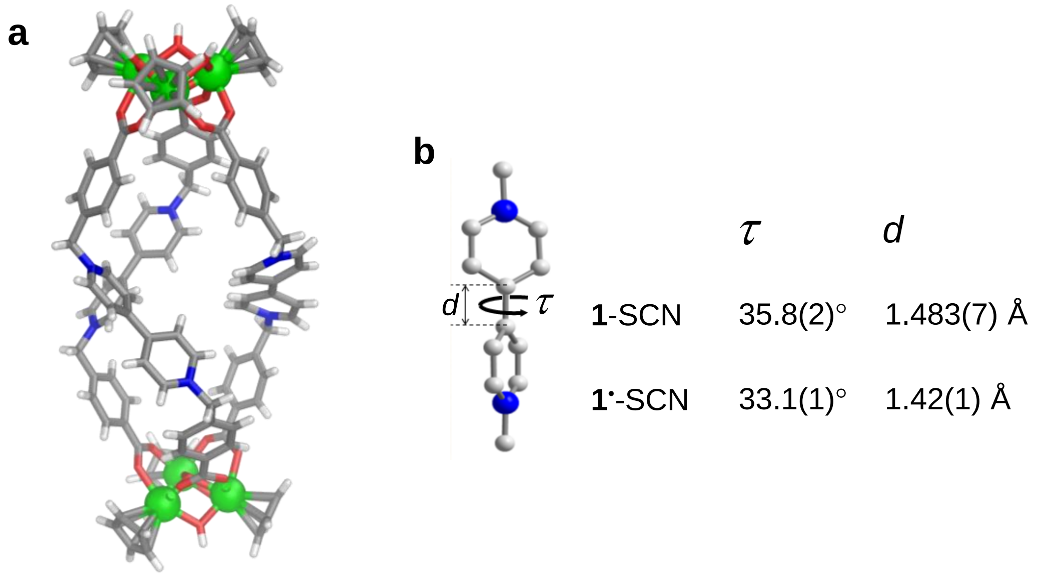

Supplementary Fig. 52 | (a) X-ray crystallographic structure of **1**• in **1**•-SCN. (b) Structure parameters of the viologen units in **1**•-SCN compared with those in **1**-SCN.

4. Supplementary Note 4: Guest binding studies

Two sets of anionic guests, including common anions (tetrabutylammonium salts of Cl^-, Br^-, I^-, SCN^- TfO^-, ReO_4^-, NO_3^-, ClO_4^-, PF_6^-, and Tf_2N^-) and organic tetraarylborate anions (sodium salts of $[B(Ph\text{-}OCH_3)_4]^-$, $[B(Ph\text{-}CH_3)_4]^-$, BPh_4^-, $[B(Ph\text{-}Cl)_4]^-$, and $[B(Ph\text{-}F)_4]^-$), were investigated for the binding properties of **1**-BAr_F. ^1H NMR titrations were carried out by adding guests into a methanol solution of **1**-BAr_F (1 mM). Fast-exchange binding on the NMR chemical shift time scale were observed for all the cases. Binding constants were calculated using BINDFIT[6] with the data obtained from ^1H NMR titrations.

Supplementary Table. 1 | Binding constants (K_a) of **1**-BAr_F for common anions in CH_3OH.

guest	K_a (M^{-1})
I^-	$(2.3±0.2)×10^3$
Br^-	$(2.1±0.2)×10^3$
Cl^-	$(1.3±0.1)×10^3$
SCN^-	$(7.3±0.7)×10^2$
TfO^-	$(7.7±0.8)×10^2$
ReO_4^-	$(7.2±0.7)×10^2$
NO_3^-	$(5.8±0.4)×10^2$
ClO_4^-	$(5.2±0.4)×10^2$
PF_6^-	$(4.5±0.5)×10^2$
Tf_2N^-	$(4.1±0.4)×10^2$

Supplementary Table. 2 | Binding constants (K_a) of **1**-BAr_F for tetraarylborate anions in CH_3OH.

guest	K_a (M^{-1})
$[B(Ph\text{-}OCH_3)_4]^-$	$(4.0±0.4)×10^3$
$[B(Ph\text{-}CH_3)_4]^-$	$(3.6±0.4)×10^3$
BPh_4^-	$(3.4±0.3)×10^3$
$[B(Ph\text{-}Cl)_4]^-$	$(3.1±0.3)×10^3$
$[B(Ph\text{-}F)_4]^-$	$(2.8±0.3)×10^3$

4.1 ^{1}H NMR titrations of common anions

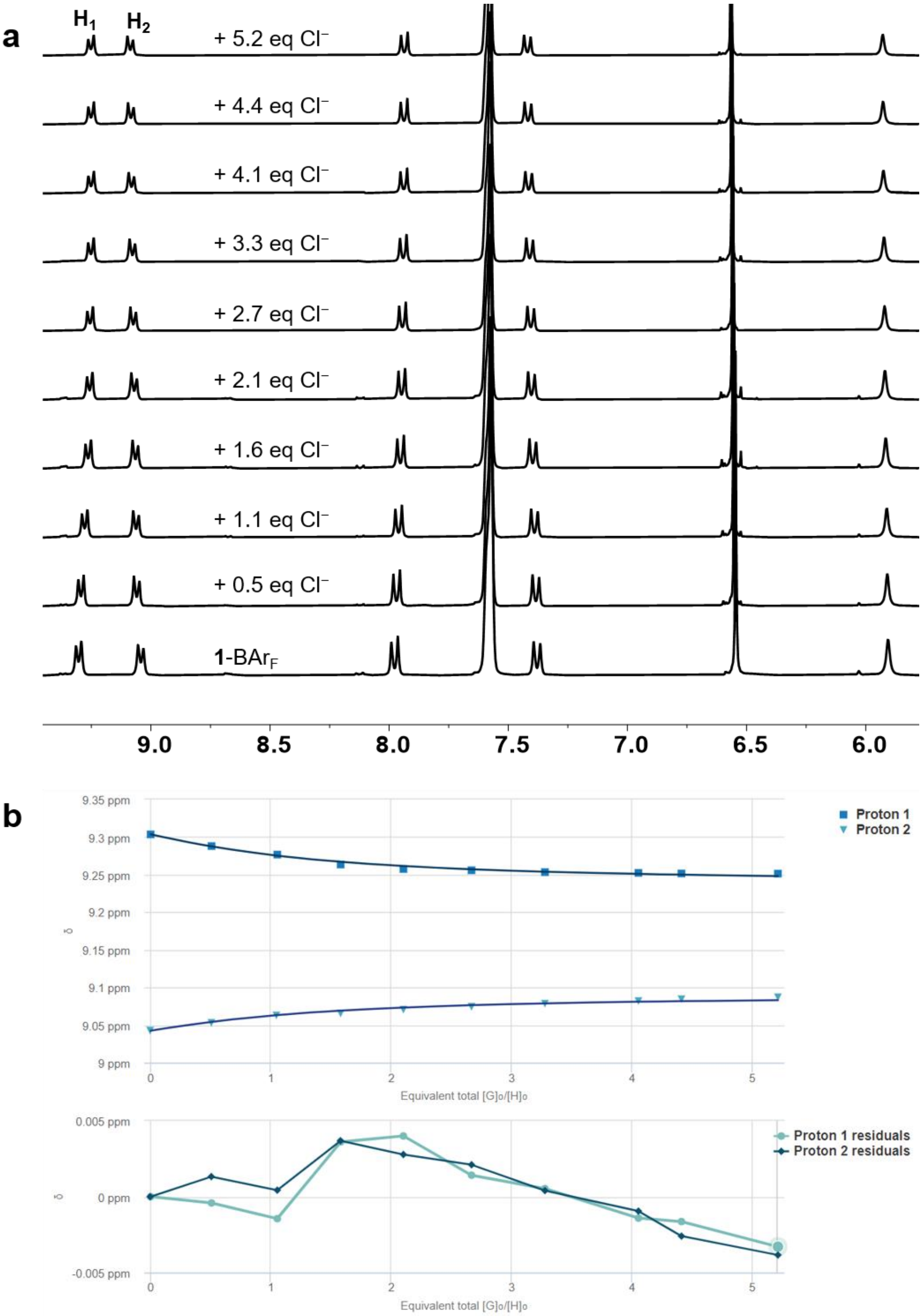

Supplementary Fig. 53 | ^{1}H NMR (CD_3OD, 400 MHz, 298 K) titrations of Cl^- into a methanol solution of **1**-BAr_F (1.0 mM) (a) and the corresponding binding isotherms (1:1 system) fitted by BINDFIT (b). A binding constant of $(1.3\pm0.1)\times10^3$ M^{-1} was obtained.

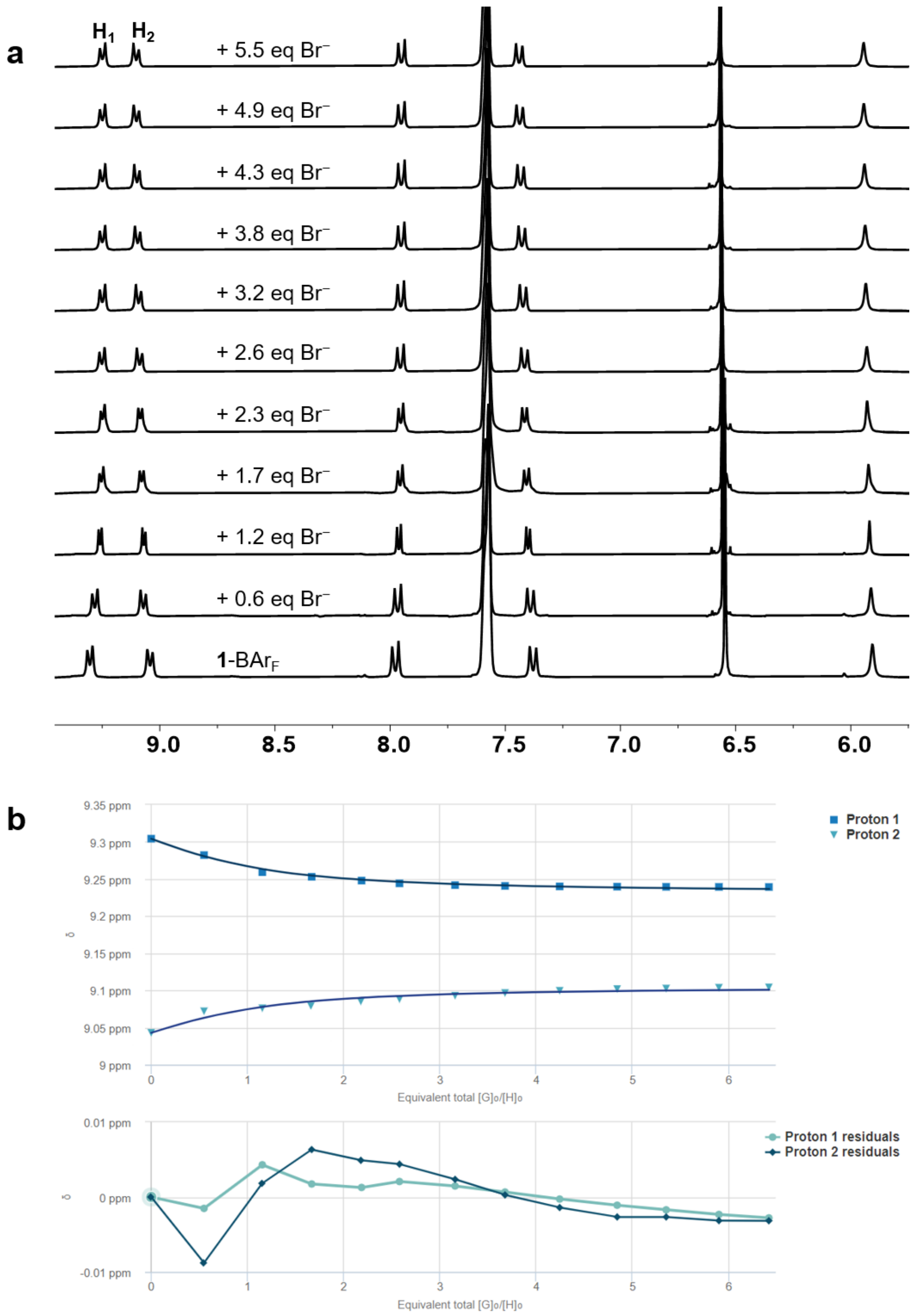

Supplementary Fig. 54 | ^{1}H NMR (CD_3OD, 400 MHz, 298 K) titrations of Br^- into a methanol solution of **1**-BAr$_F$ (1.0 mM) (a) and the corresponding binding isotherms (1:1 system) fitted by BINDFIT (b). A binding constant of $(2.1\pm0.2)\times10^3$ M^{-1} was obtained.

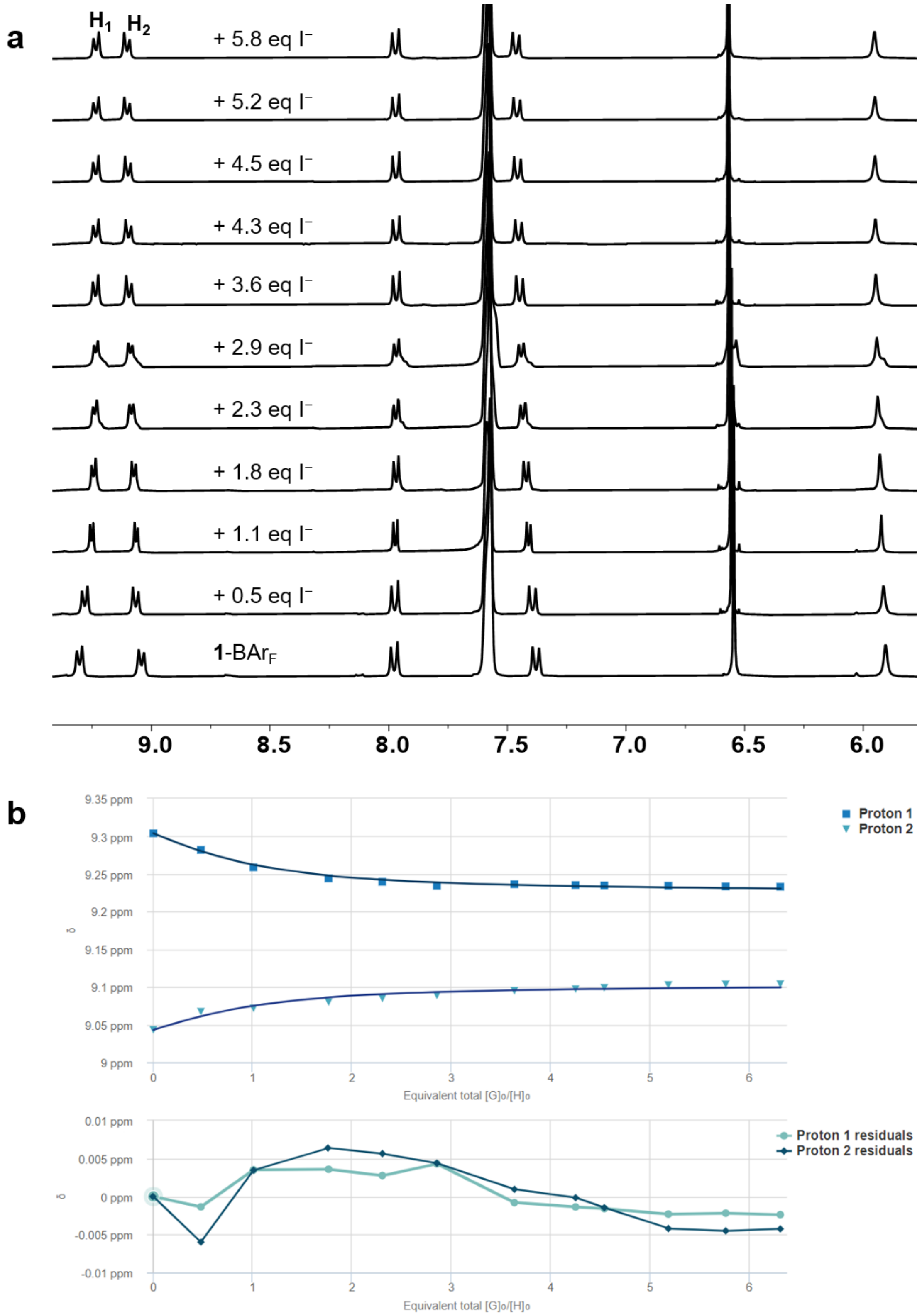

Supplementary Fig. 55 | ^{1}H NMR (CD_3OD, 400 MHz, 298 K) titrations of I^- into a methanol solution of **1**-BAr_F (1.0 mM) (a) and the corresponding binding isotherms (1:1 system) fitted by BINDFIT (b). A binding constant of $(2.3\pm0.2)\times10^3$ M^{-1} was obtained.

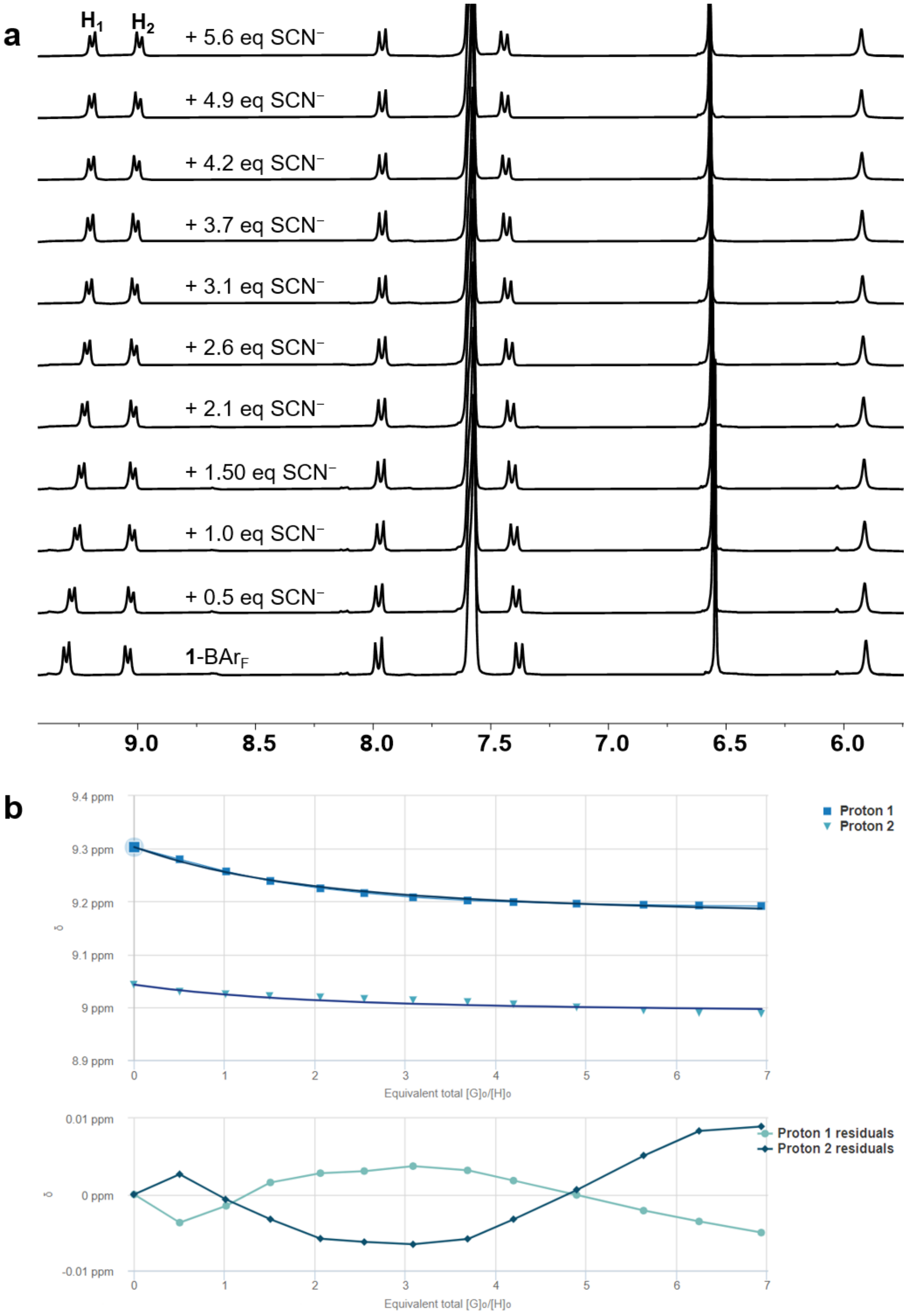

Supplementary Fig. 56 | ^{1}H NMR (CD_3OD, 400 MHz, 298 K) titrations of SCN^- into a methanol solution of **1**-BAr_F (1.0 mM) (a) and the corresponding binding isotherms (1:1 system) fitted by BINDFIT (b). A binding constant of $(7.3\pm0.7)\times10^2$ M^{-1} was obtained.

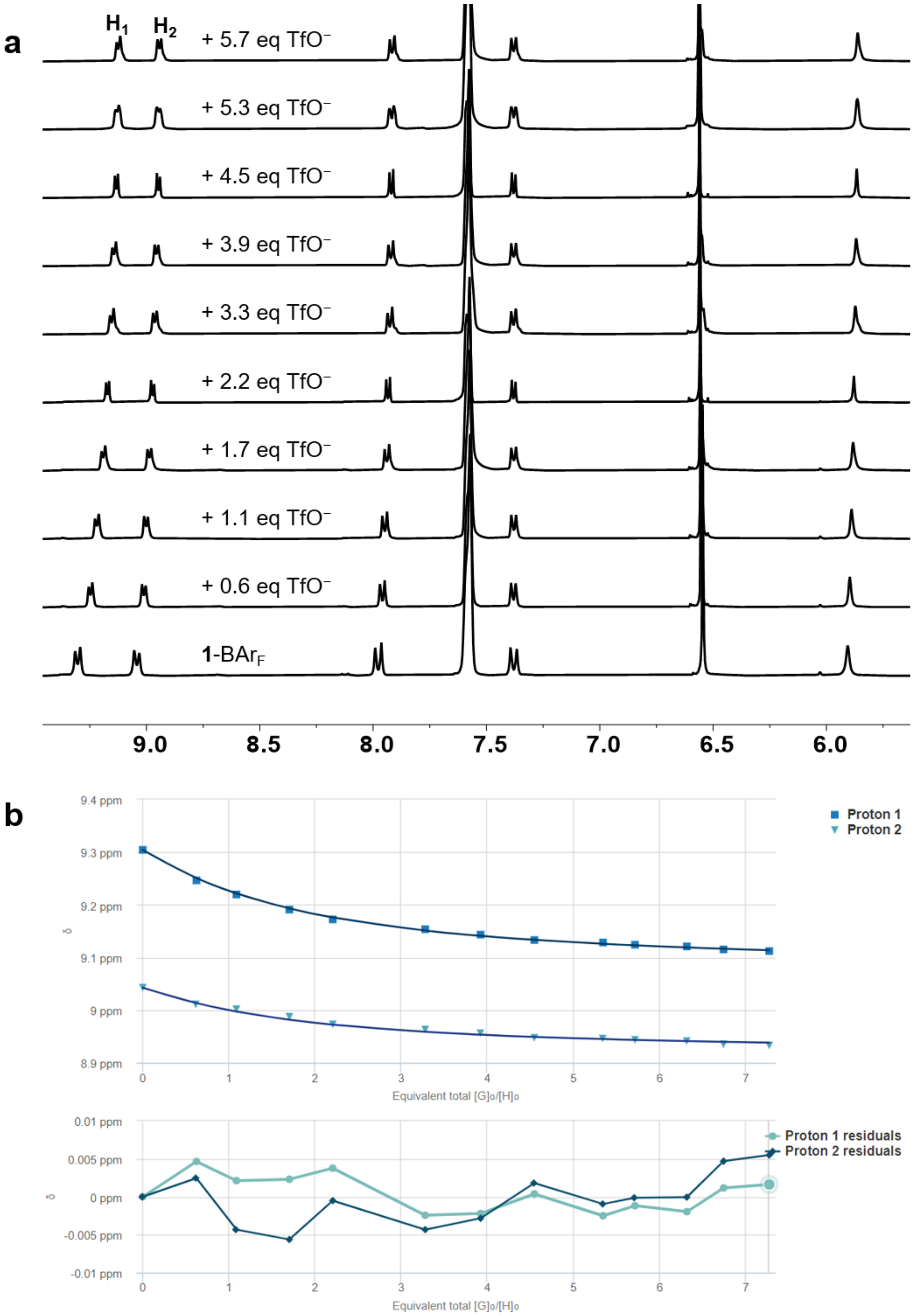

Supplementary Fig. 57 | ^{1}H NMR (CD_3OD, 400 MHz, 298 K) titrations of TfO^- into a methanol solution of **1**-BAr_F (1.0 mM) (a) and the corresponding binding isotherms (1:1 system) fitted by BINDFIT (b). A binding constant of (7.7±0.8)×10^2 M^{-1} was obtained.

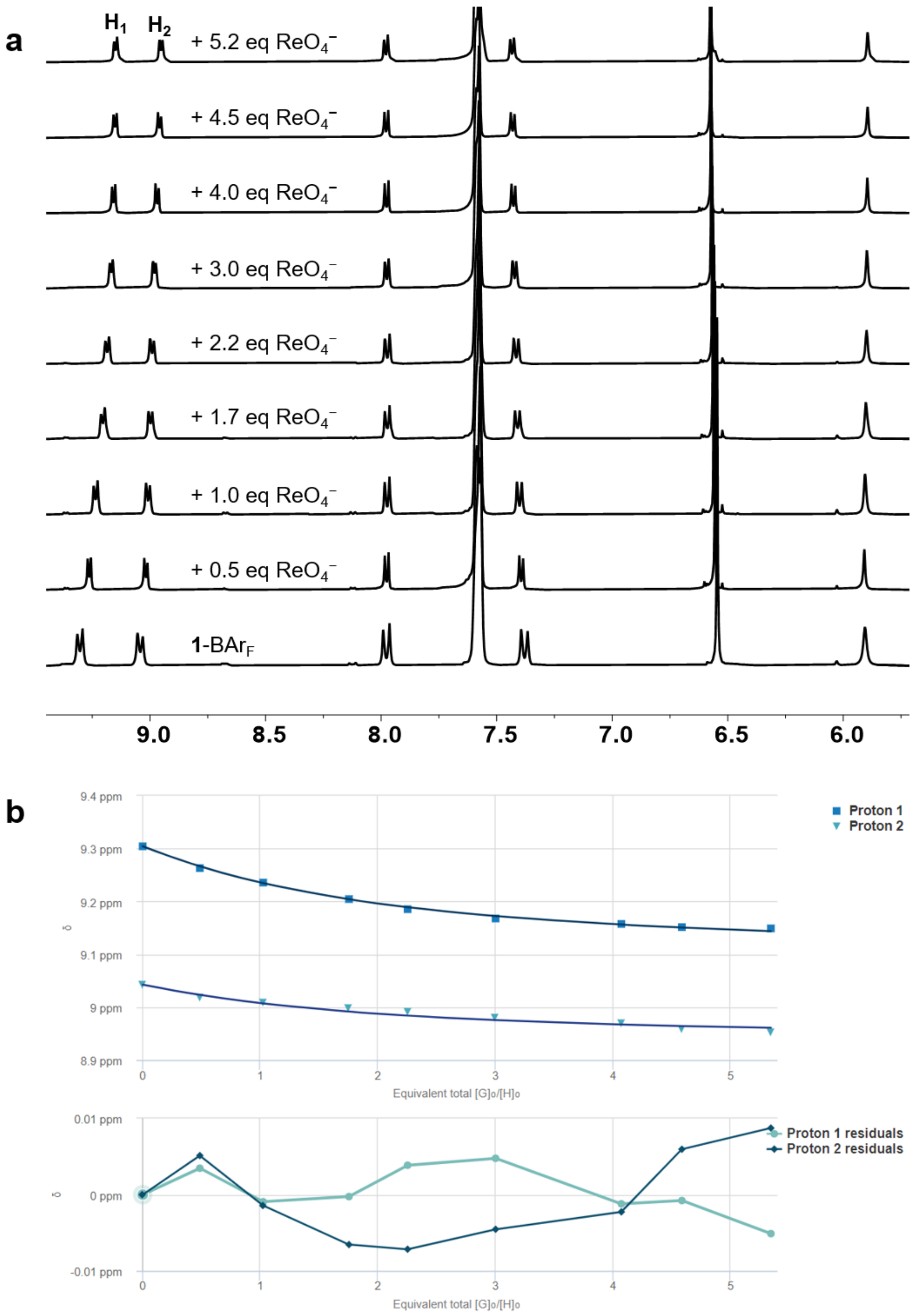

Supplementary Fig. 58 | ^{1}H NMR (CD_3OD, 400 MHz, 298 K) titrations of ReO_4^- into a methanol solution of **1**-BAr_F (1.0 mM) (a) and the corresponding binding isotherms (1:1 system) fitted by BINDFIT (b). A binding constant of $(7.2\pm0.7)\times10^2$ M^{-1} was obtained.

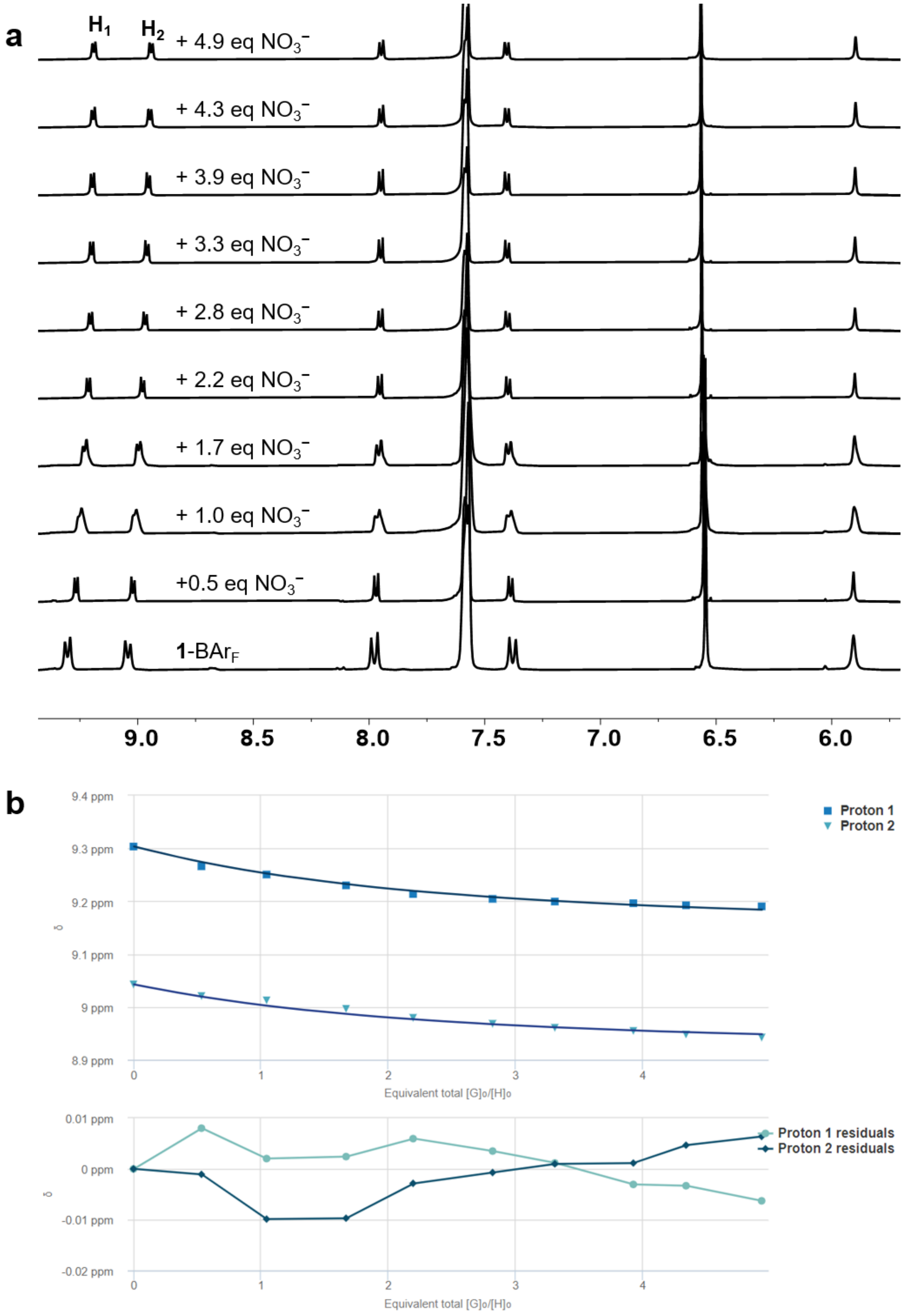

Supplementary Fig. 59 I ^{1}H NMR (CD_3OD, 400 MHz, 298 K) titrations of NO_3^- into a methanol solution of **1**-BAr_F (1.0 mM) (a) and the corresponding binding isotherms (1:1 system) fitted by BINDFIT (b). A binding constant of $(5.8\pm0.4)\times10^{2}$ M^{-1} was obtained.

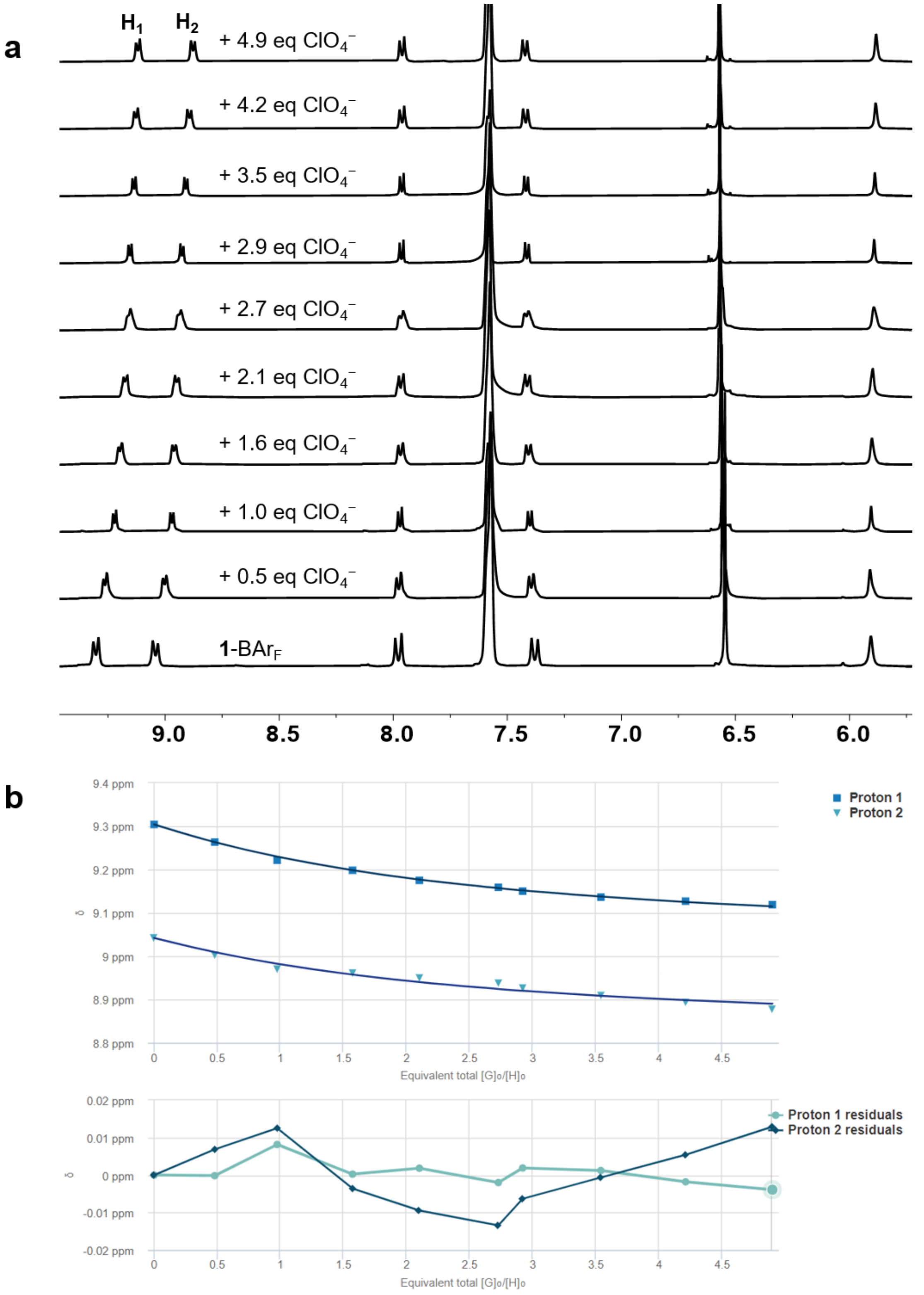

Supplementary Fig. 60 l ^{1}H NMR (CD_3OD, 400 MHz, 298 K) titrations of ClO_4^- into a methanol solution of **1**-BAr_F (1.0 mM) (a) and the corresponding binding isotherms (1:1 system) fitted by BINDFIT (b). A binding constant of $(5.2\pm0.4)\times10^2$ M^{-1} was obtained.

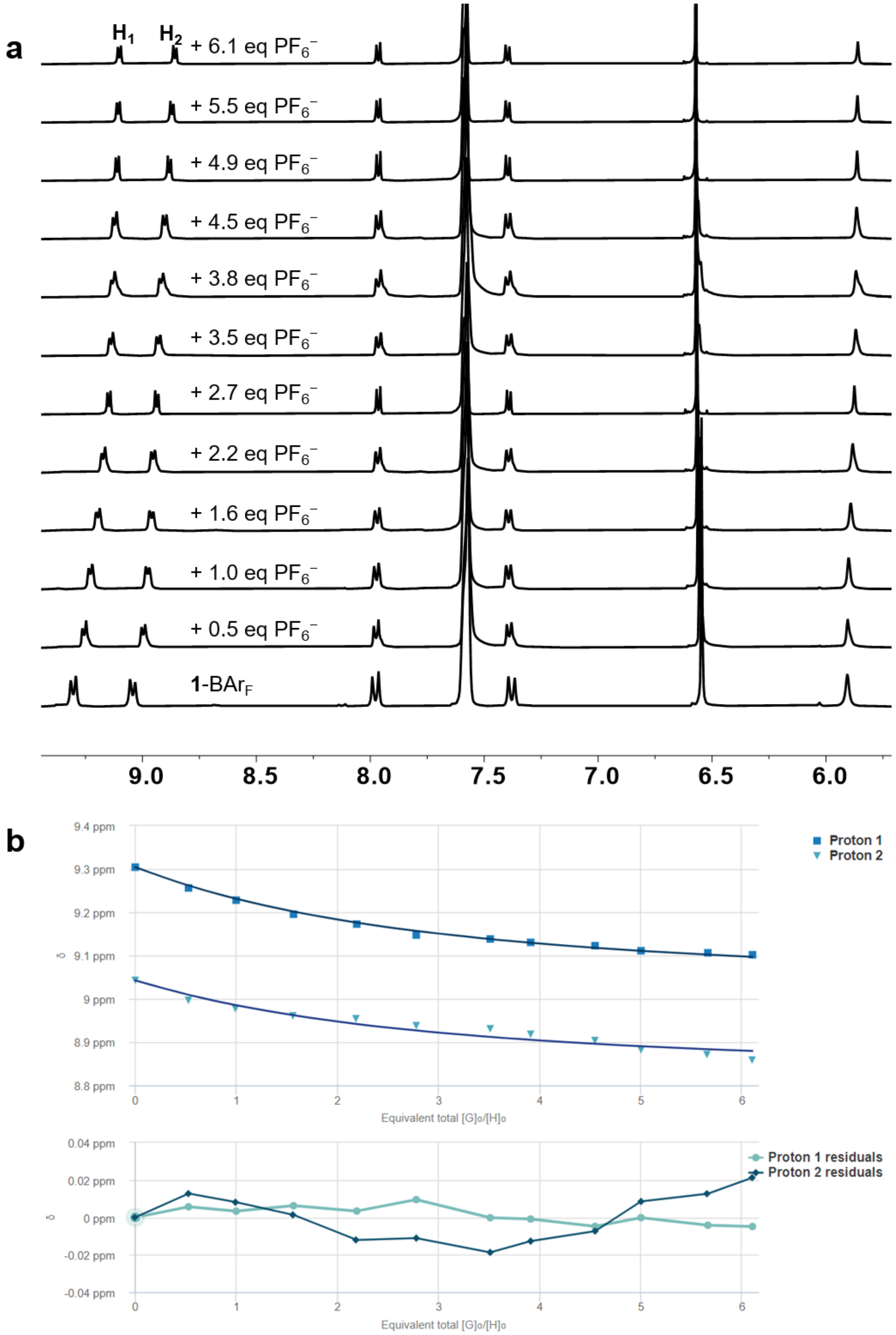

Supplementary Fig. 61 | ^{1}H NMR (CD_3OD, 400 MHz, 298 K) titrations of PF_6^- into a methanol solution of **1**-BAr$_F$ (1.0 mM) (a) and the corresponding binding isotherms (1:1 system) fitted by BINDFIT (b). A binding constant of $(4.5\pm0.5)\times10^2$ M^{-1} was obtained.

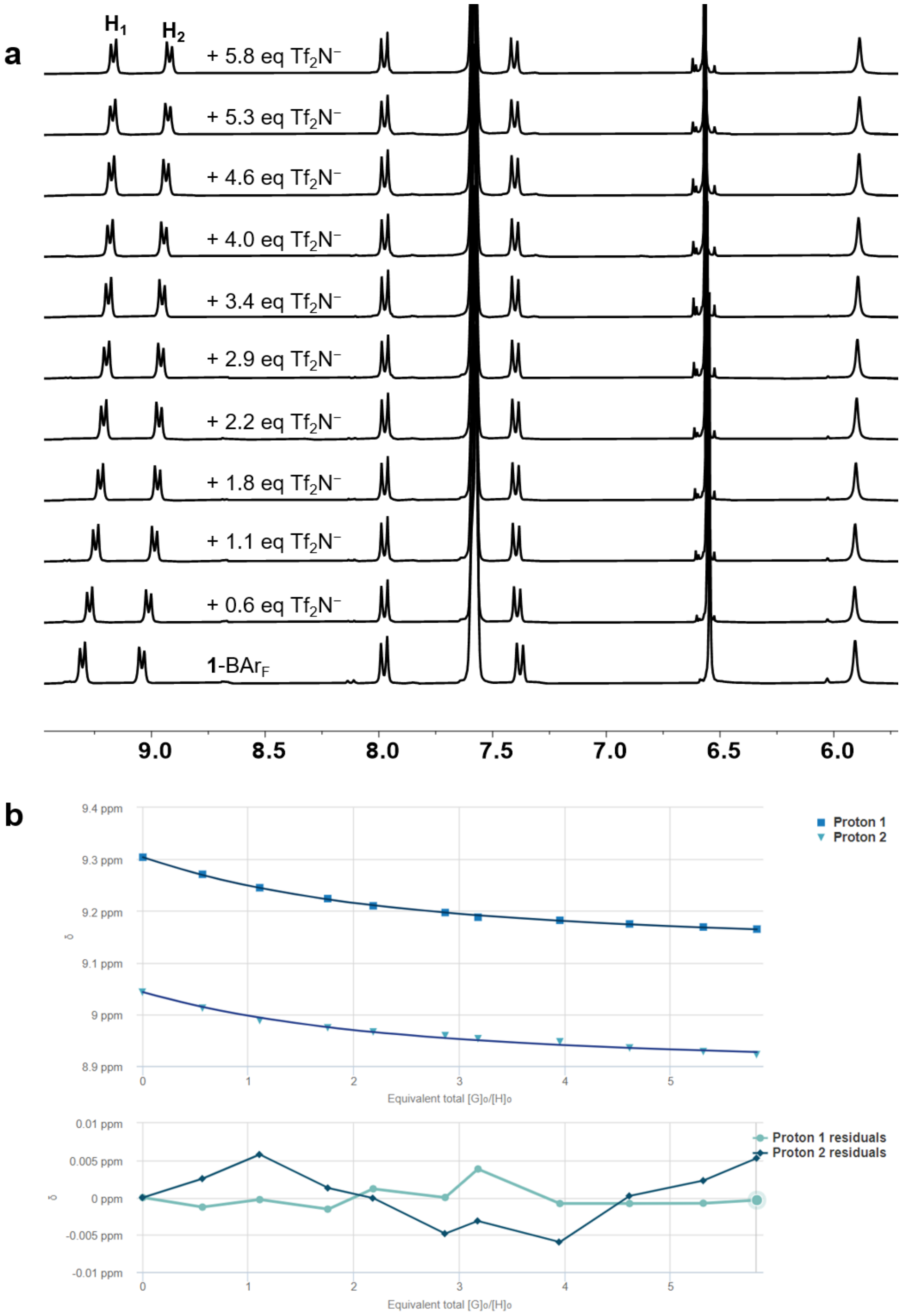

Supplementary Fig. 62 l ^{1}H NMR (CD_3OD, 400 MHz, 298 K) titrations of Tf_2N^- into a methanol solution of **1**-BAr$_F$ (1.0 mM) (a) and the corresponding binding isotherms (1:1 system) fitted by BINDFIT (b). A binding constant of $(4.1\pm0.4)\times10^2$ M^{-1} was obtained.

4.2 ^{1}H NMR titrations of tetraarylborate anions

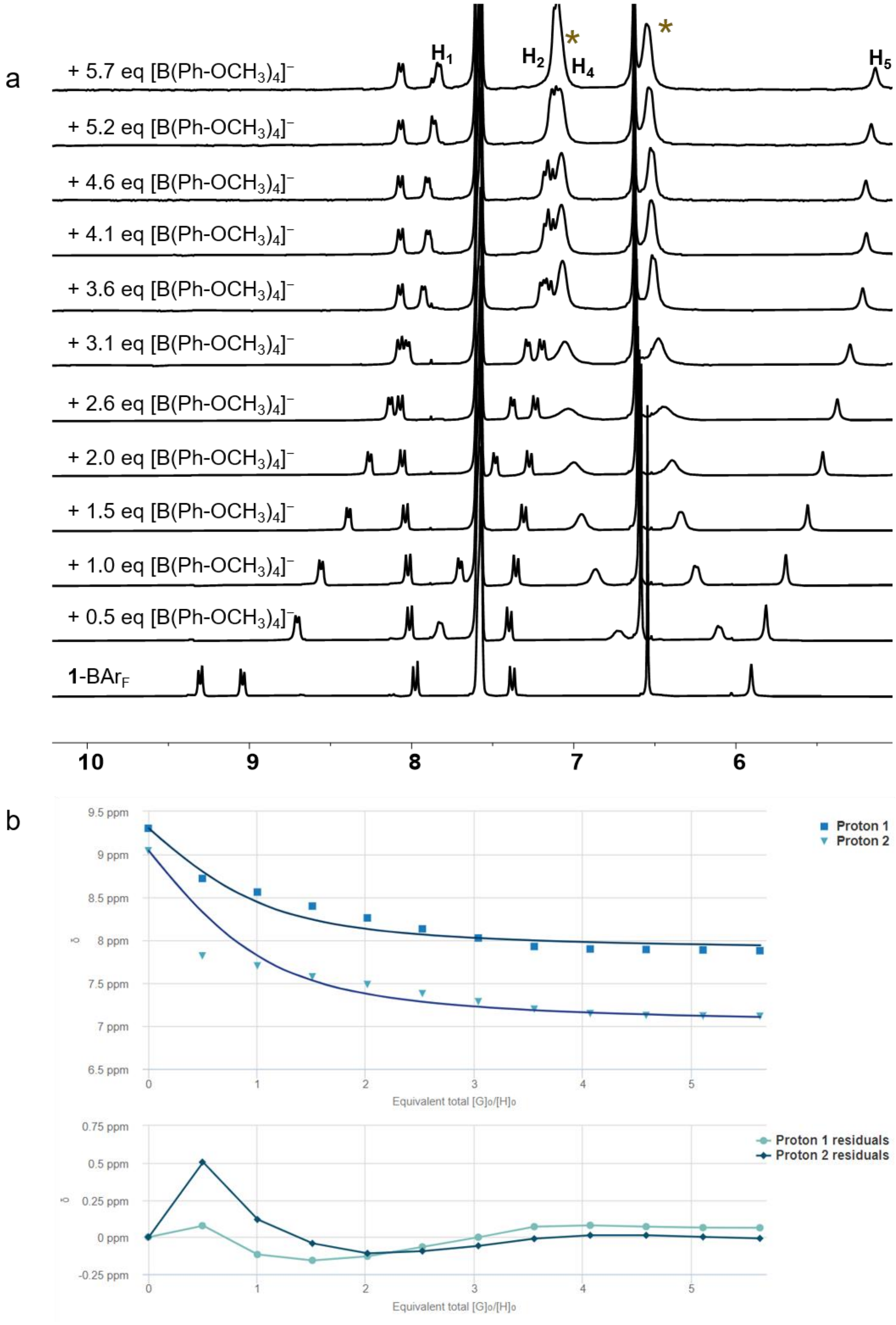

Supplementary Fig. 63 I ^{1}H NMR (CD_3OD, 400 MHz, 298 K) titrations of $[B(Ph\text{-}OCH_3)_4]^-$ into a methanol solution of **1**-BAr$_F$ (1.0 mM) (a) and the corresponding binding isotherms (1:1 system) fitted by BINDFIT, peaks from the guest are indicated by asterisks. (b) A binding constant of $(4.0\pm0.4)\times10^3$ M^{-1} was obtained

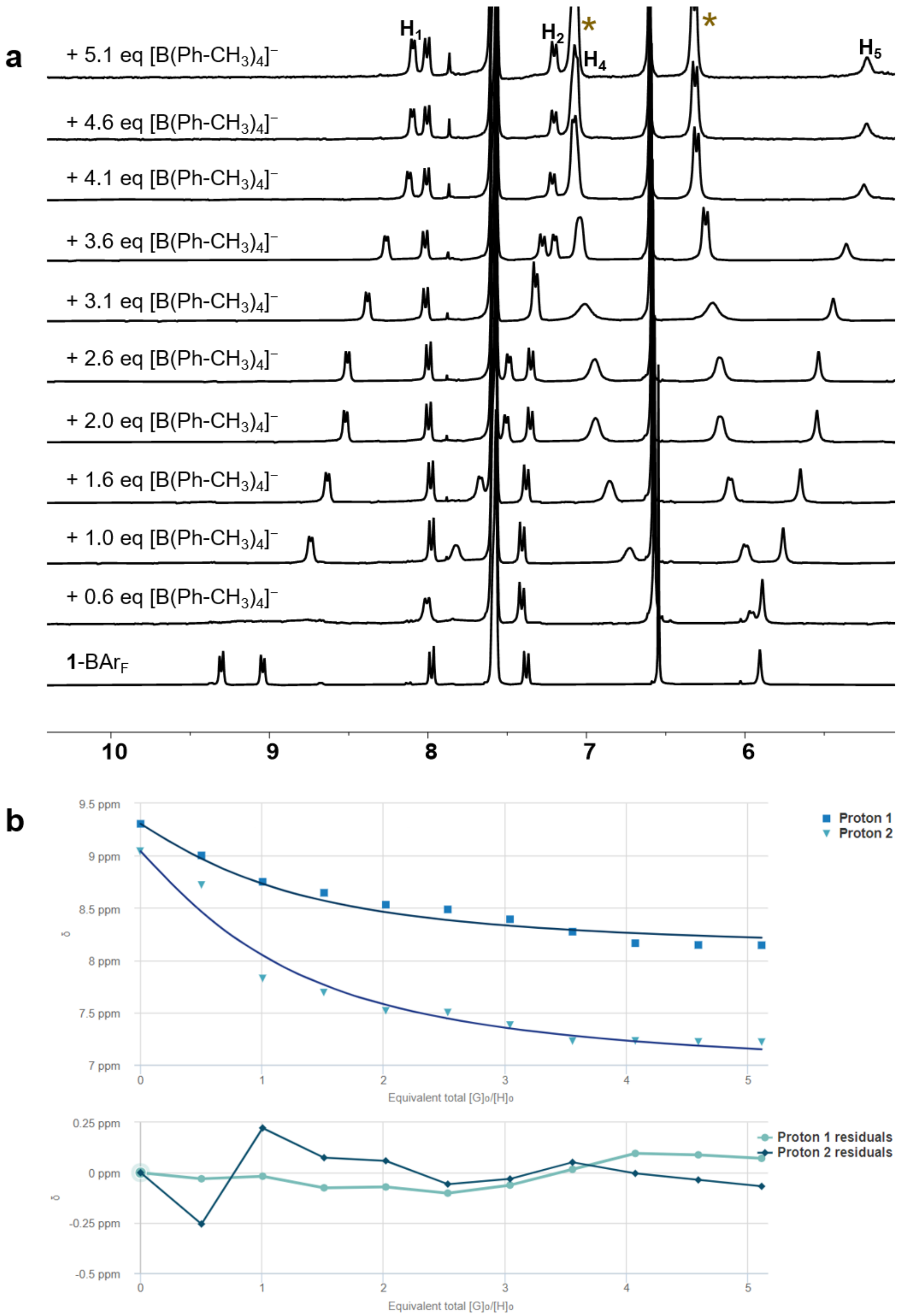

Supplementary Fig. 64 | ^{1}H NMR (CD_3OD, 400 MHz, 298 K) titrations of $[B(Ph\text{-}CH_3)_4]^-$ into a methanol solution of **1**-BAr_F (1.0 mM) (a) and the corresponding binding isotherms (1:1 system) fitted by BINDFIT, peaks from the guest are indicated by asterisks. (b) A binding constant of $(3.6\pm0.4)\times10^3$ M^{-1} was obtained.

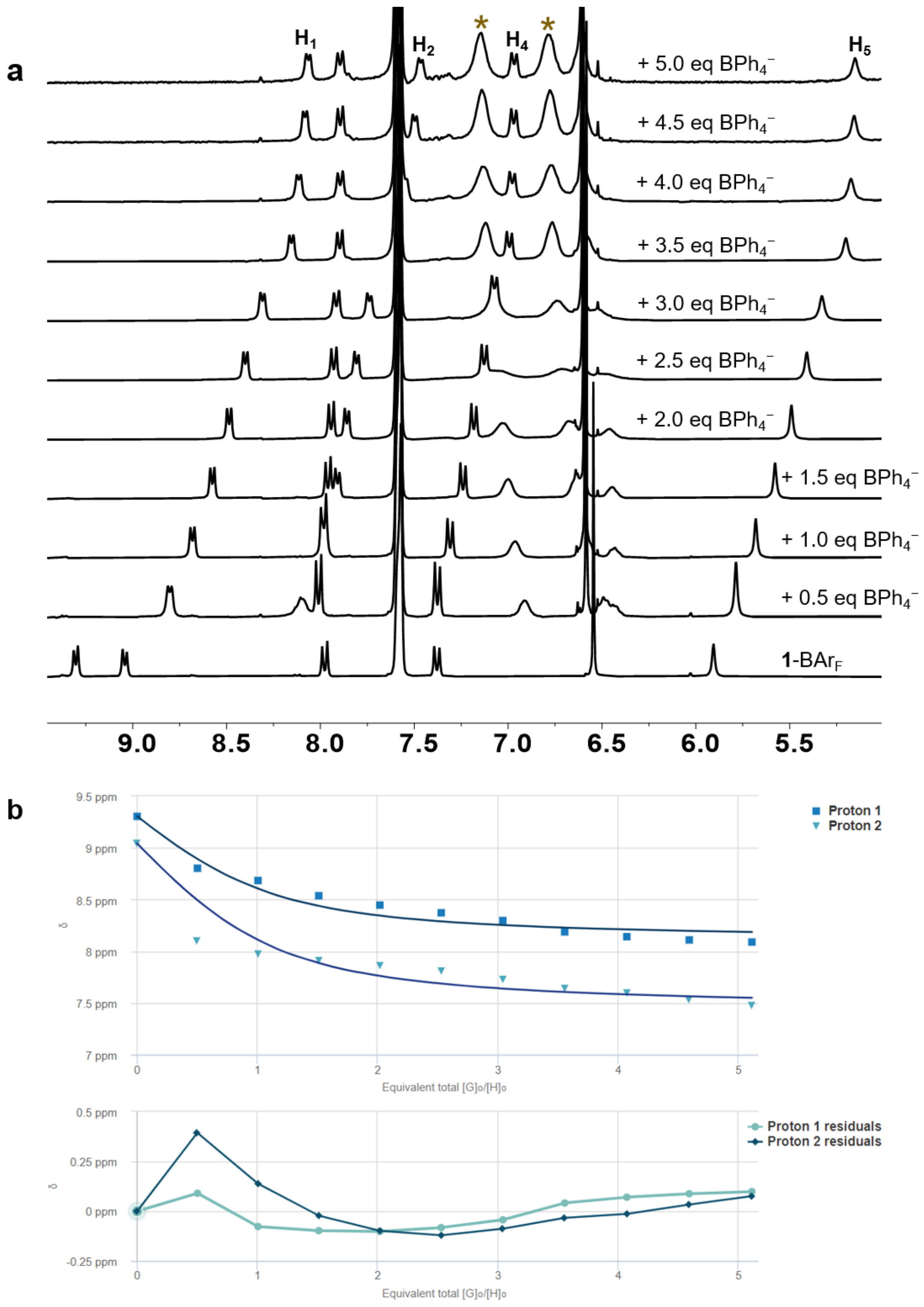

Supplementary Fig. 65 l ^{1}H NMR (CD_3OD, 400 MHz, 298 K) titrations of BPh_4^- into a methanol solution of **1**-BAr_F (1.0 mM) (a) and the corresponding binding isotherms (1:1 system) fitted by BINDFIT, peaks from the guest are indicated by asterisks. (b) A binding constant of $(3.4\pm0.3)\times10^3$ M^{-1} was obtained.

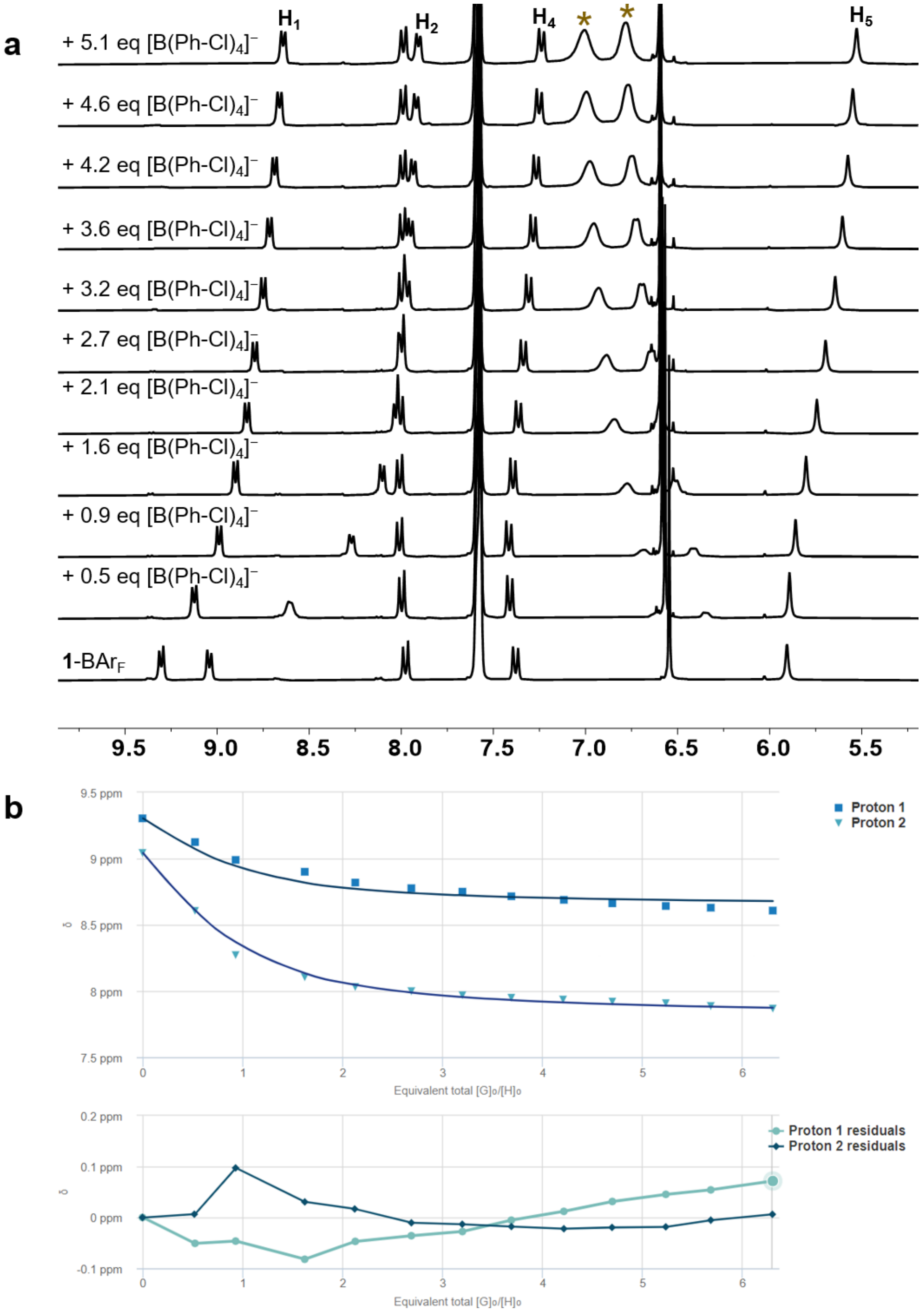

Supplementary Fig. 66 | **^{1}H NMR (CD_3OD, 400 MHz, 298 K) titrations of $[B(Ph\text{-}Cl)_4]^-$ into a methanol solution of **1-BAr_F (1.0 mM) (a) and the corresponding binding isotherms (1:1 system) fitted by BINDFIT, peaks from the guest are indicated by asterisks. (b) A binding constant of $(3.1\pm0.3)\times10^3$ M^{-1} was obtained.

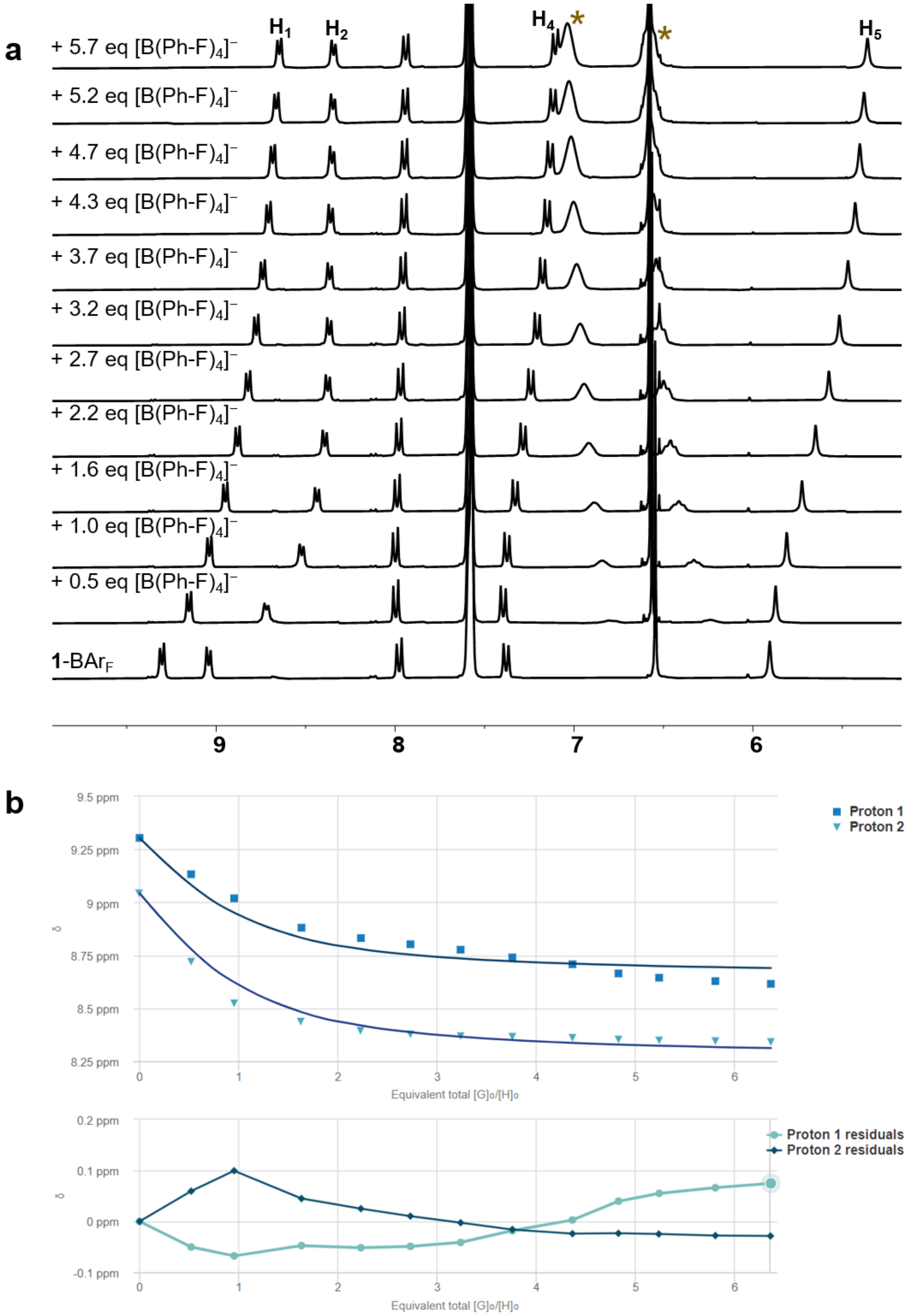

Supplementary Fig. 67 l ^{1}H NMR (CD_3OD, 400 MHz, 298 K) titrations of $[B(Ph\text{-}F)_4]^-$ into a methanol solution of **1**-BAr_F (1.0 mM) (a) and the corresponding binding isotherms (1:1 system) fitted by BINDFIT, peaks from the guest are indicated by asterisks. (b) A binding constant of $(2.8\pm0.3)\times10^3$ M^{-1} was obtained.

4.3 UV-vis spectrophotometric titrations

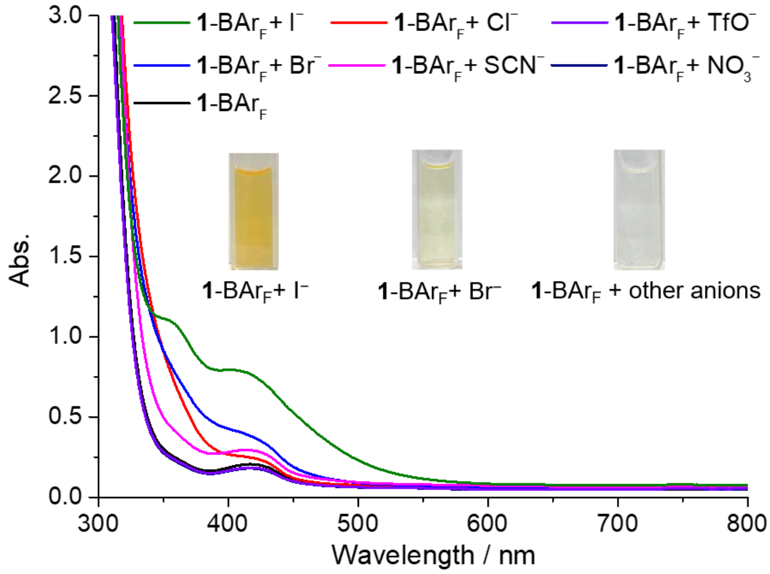

Supplementary Fig. 68 | UV-vis spectra of **1**-BAr$_F$ (0.2 mM in CH_3OH) in the absence or presence of 8 equiv. I^-, Br^-, Cl^-, SCN^-, NO_3^- or TfO^-. Insets: photographs showing the color of the solutions.

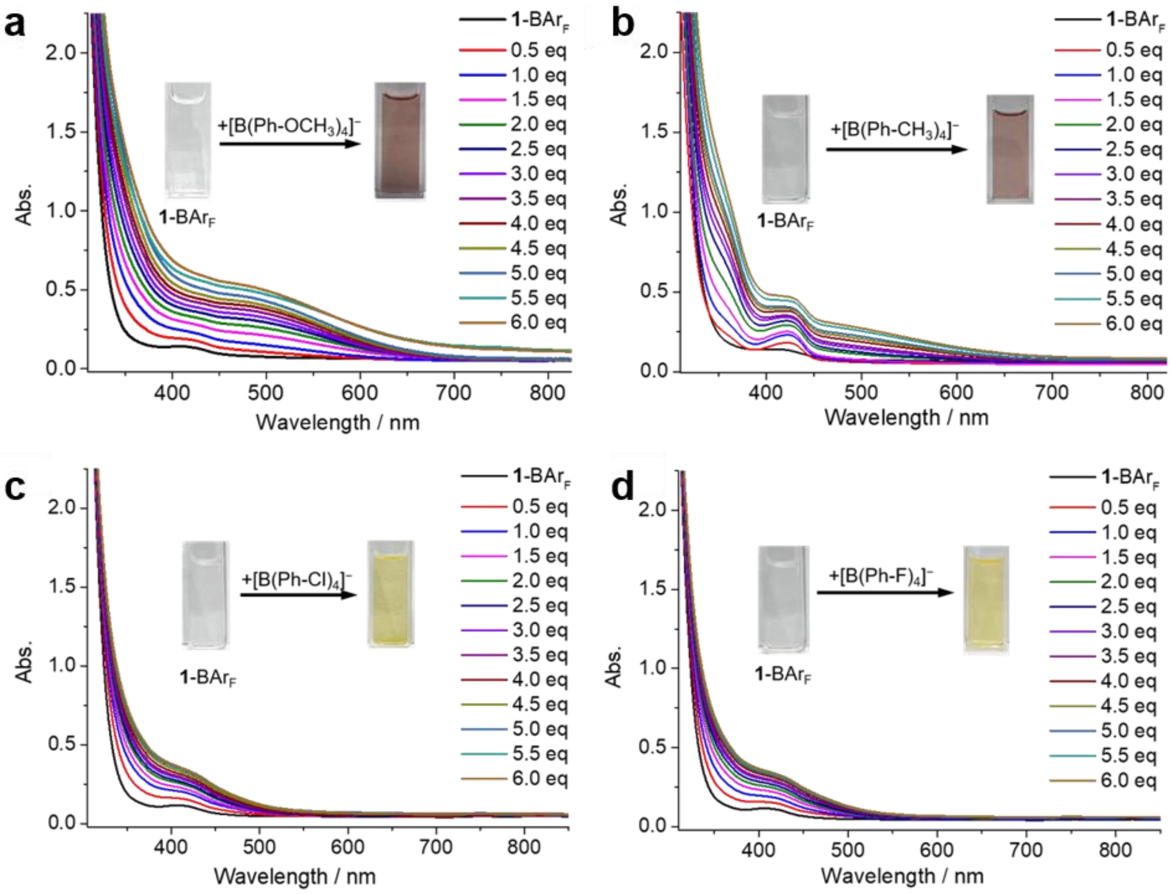

Supplementary Fig. 69 | UV-vis spectrophotometric titrations of $[B(Ph\text{-}OCH_3)_4]^-$ (a), $[B(Ph\text{-}CH_3)_4]^-$(b), $[B(Ph\text{-}Cl)_4]^-$ (c) and $[B(Ph\text{-}F)_4]^-$(d) into a methanol solution of **1**-BAr$_F$ (0.1 mM). Insets: photographs showing the color changes of the solution upon addition of tetraarylborate anions.

5. Supplementary Note 5: Photochromism studies

5.1 Photochromism of 1 in the presence of tetraarylborates

In all cases, solutions of **1** were degassed and filled with inert N_2 through the freeze-pump-thaw cycling method. After transferring the air-free solution into a cuvette in a glovebox, the cuvette was closed and sealed. Xe lamp was used to irradiate the sample solution and UV-vis spectra of the solution were measured at regular intervals.

Fading processes were monitored as follows: After opening the lid of the cuvette, the irradiated blue solution was stirred under an atmosphere of air. UV-vis spectra of the solution were then measured at an interval of 5 s.

6.1.1 Exploration of the electron donor for the photochromism

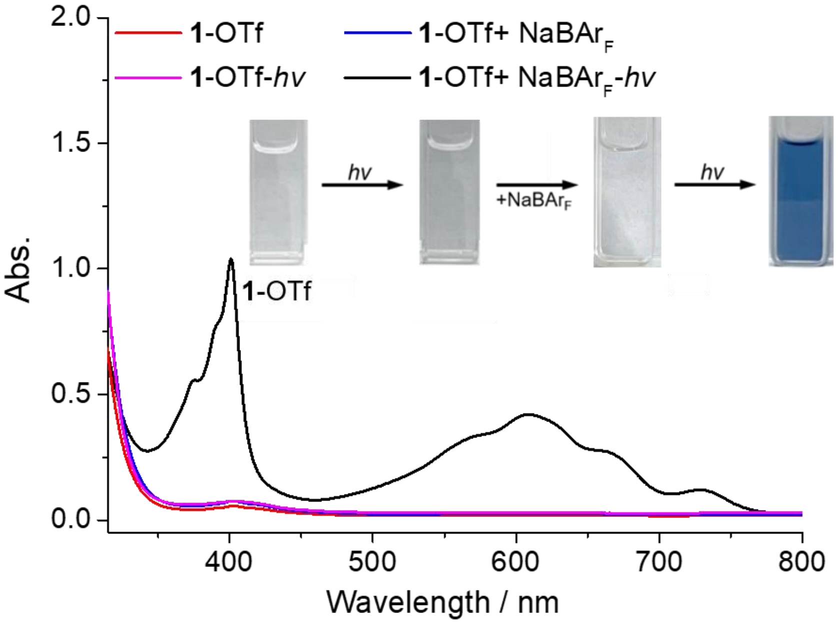

Supplementary Fig. 70 l UV-vis spectra showing the photochromic behavior of **1**-OTf (65 µM in CH_3OH) in the absence or presence of 6 equiv. BAr_F^- after irradiation for 120 s under an atmosphere of N_2. Insets: photographs showing the color changes of the solution.

6.1.2 Photochromism of 1-OTf in the presence of tetraarylborates

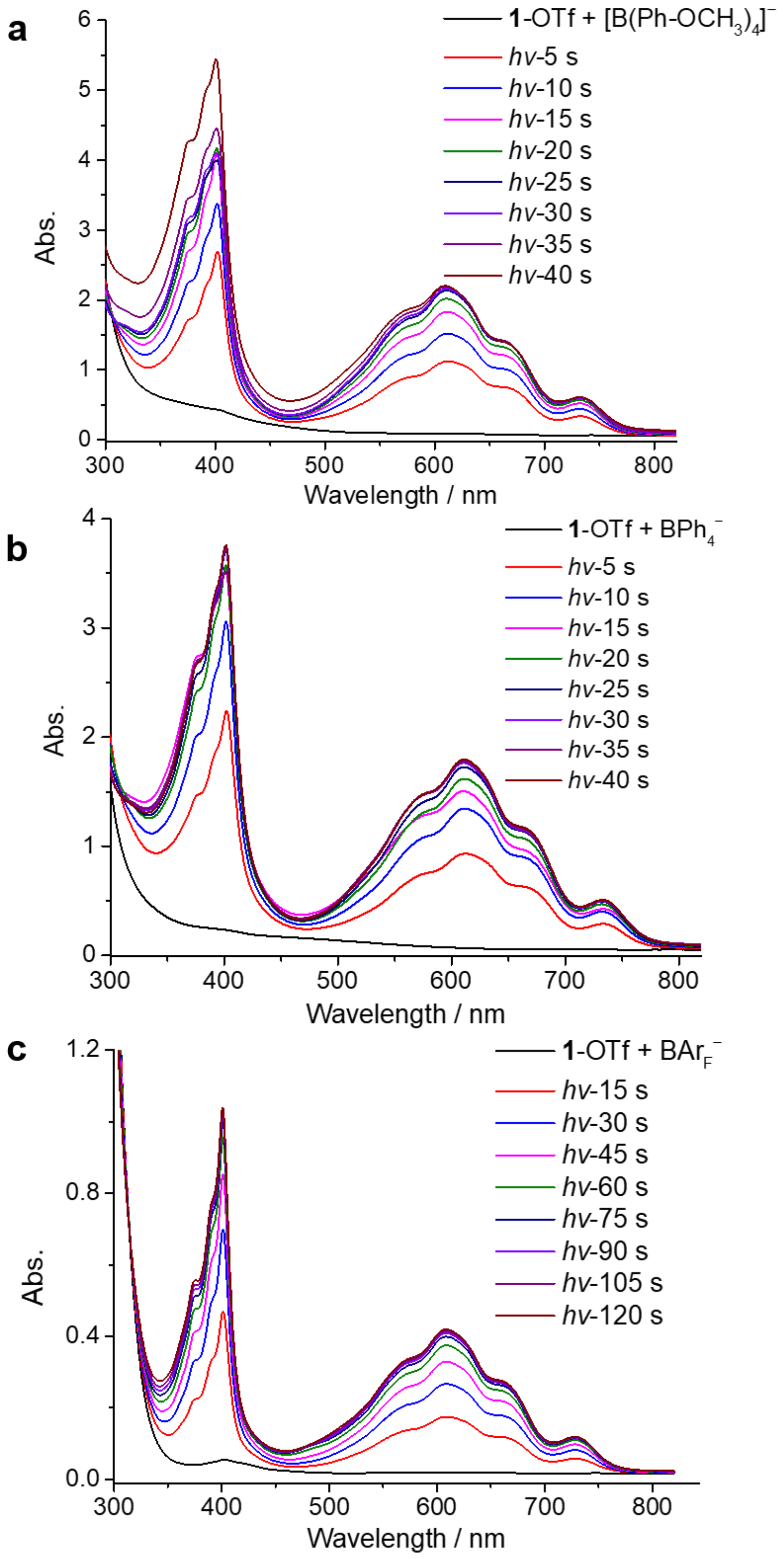

Supplementary Fig. 71 I UV-vis spectra showing the photochromic behavior of **1**-OTf (65 μM in CH_3OH) in the presence of 6 equiv. $[B(Ph\text{-}OCH_3)_4]^-$ (a), BPh_4^- (b), or BAr_F^- (c) under an atmosphere of N_2.

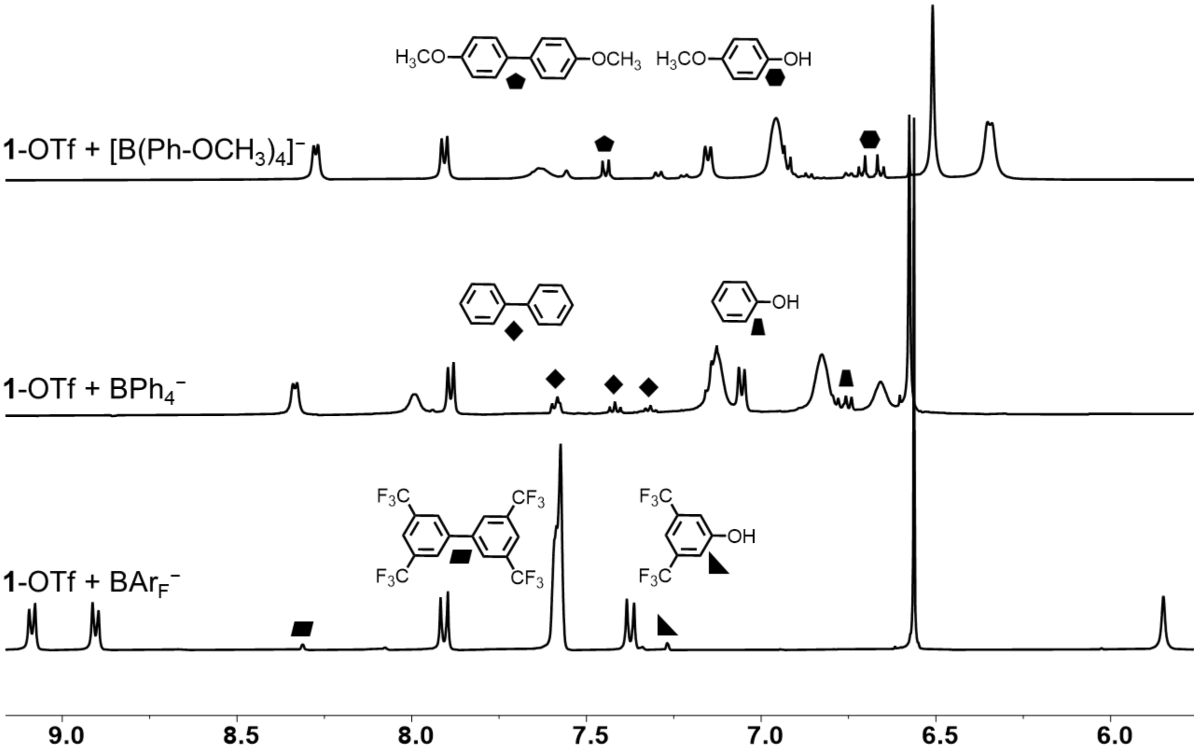

Supplementary Fig. 72 l ^{1}H NMR (CD_3OD, 400 MHz, 298 K) spectra of the samples recycled from the color-faded solutions in Supplementary Fig. 71 after exposing to the air, showing the presence of aryl-coupling products in solutions.

6.1.3 Effect of solvent on photochromism of 1-BArF

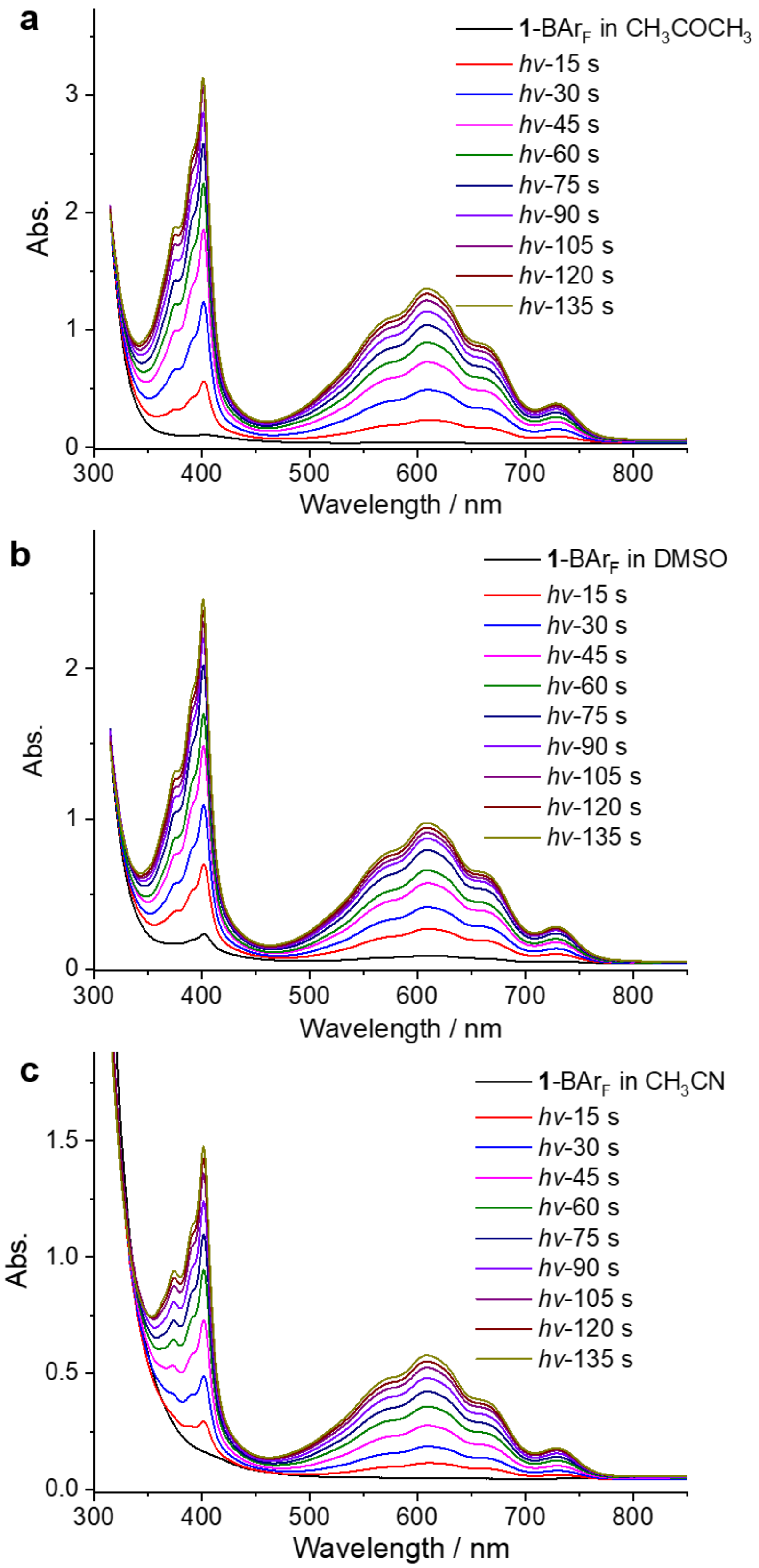

Supplementary Fig. 73 | UV-vis spectra showing the photochromic behavior of **1**-BAr$_F$ (190 µM) in CH_3COCH_3 (a), DMSO (b) or CH_3CN (c) under an atmosphere of N_2.

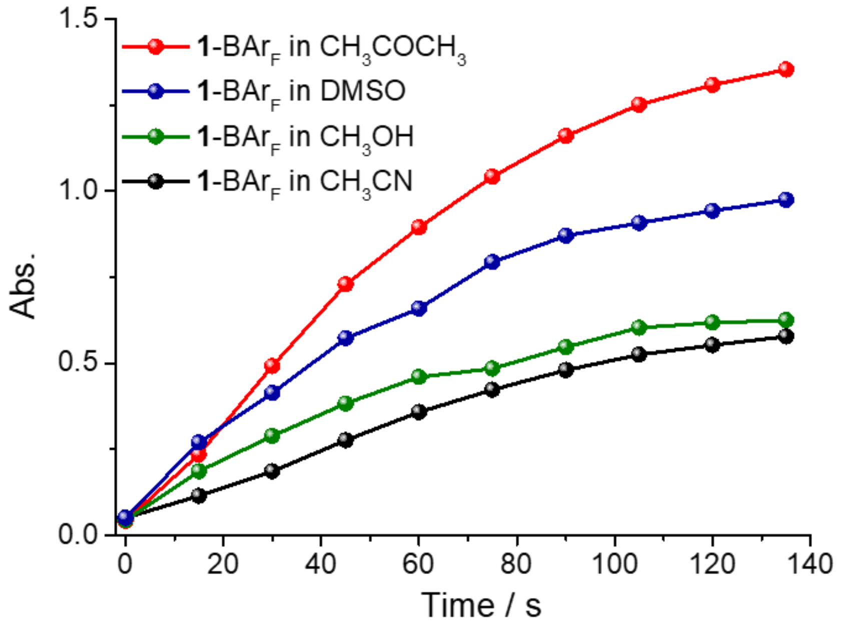

Supplementary Fig. 74 | Kinetics of photochromism of **1**-BAr_F (190 μM) in different solvents under an atmosphere of N_2. The absorbance at 612 nm was monitored.

5.2 Effect of bound anions on photochromism of 1-BAr$_F$

5.2.1 Effect of non-electron-donating anions

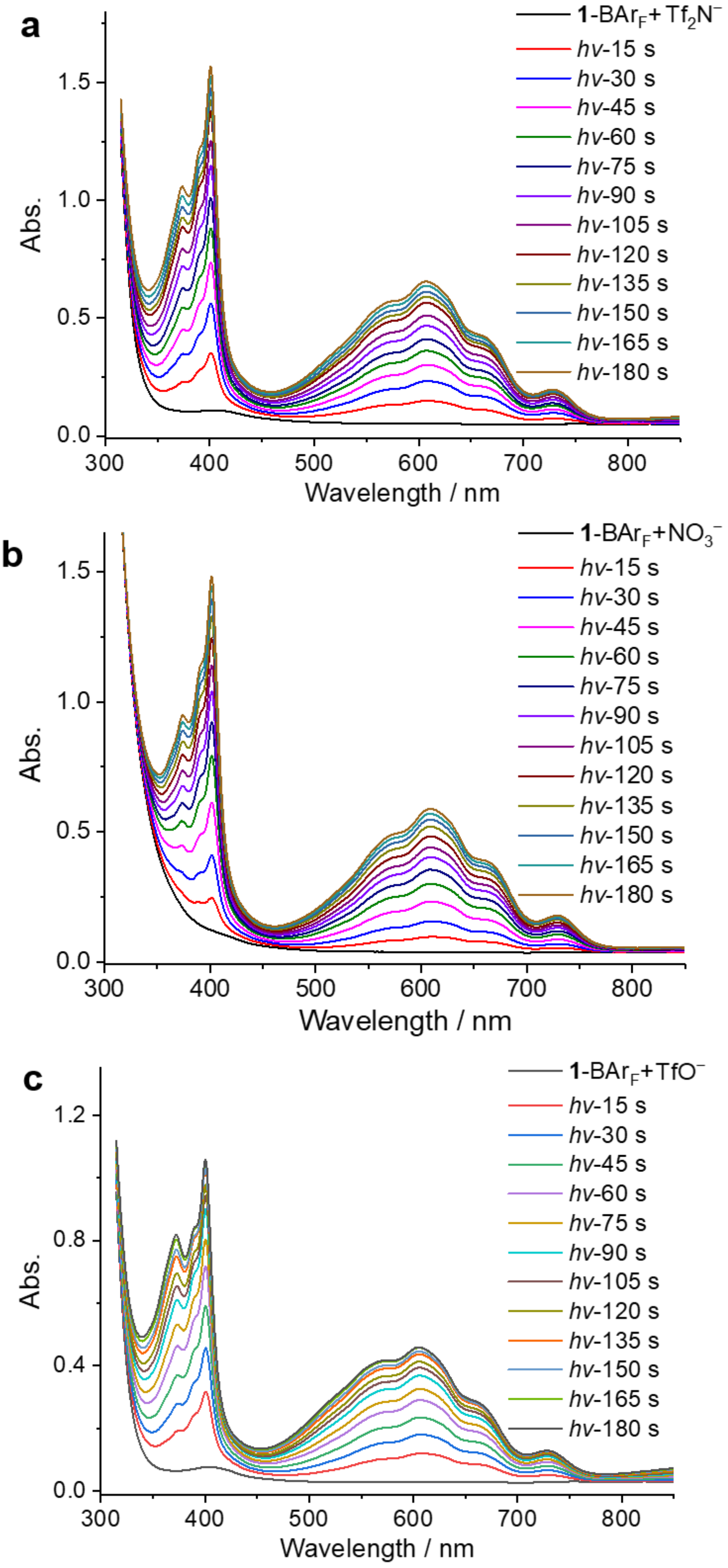

Supplementary Fig. 75 | UV-vis spectra showing the photochromic behavior of **1**-BAr$_F$ (0.19 mM in CH_3OH) in the presence of 8 equiv. Tf_2N^- (a), NO_3^- (b) or TfO^- (c) under an atmosphere of N_2.

5.2.2 Effect of electron-donating anions

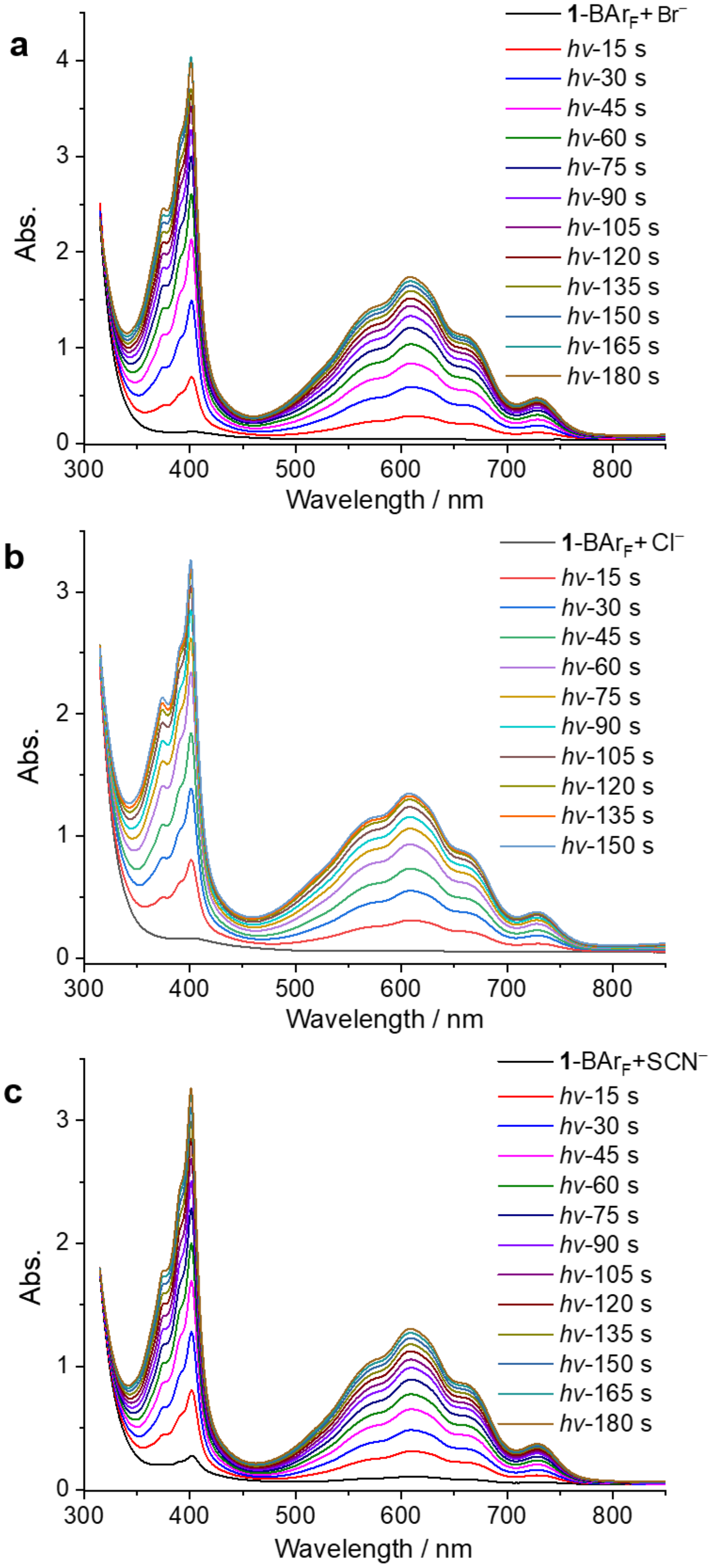

Supplementary Fig. 76 | UV-vis spectra showing the photochromic behavior of **1**-BAr_F (0.19 mM in CH_3OH) in the presence of 8 equiv. of Br^- (a), Cl^- (b) or SCN^- (c) under an atmosphere of N_2.

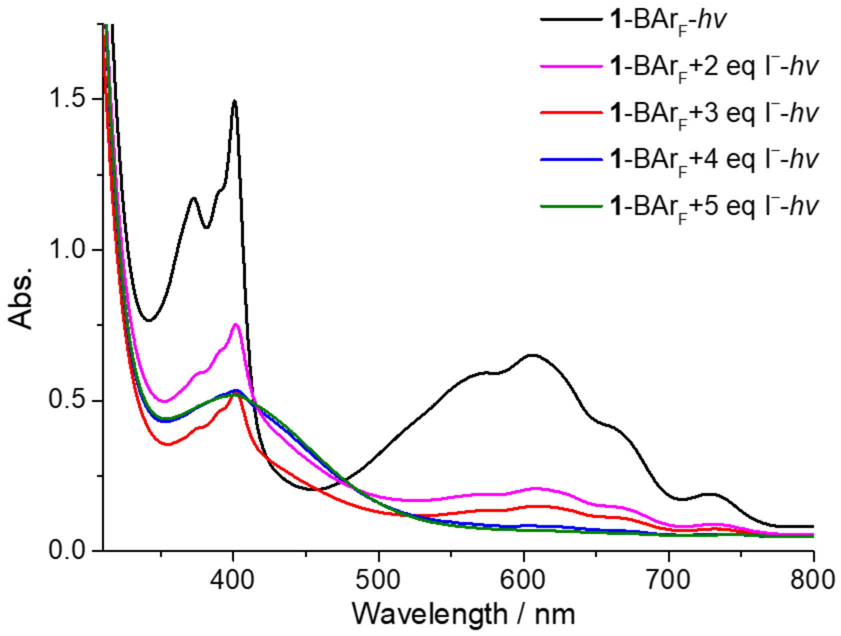

Supplementary Fig. 77 l UV-vis spectra showing the photochromic behavior of **1**-BAr$_F$ (0.19 mM in CH_3OH) for 180 s in the absence or presence of different equivalents of I^- under an atmosphere of N_2. The radical absorbance band of the cage centered around 612 nm does not appear upon irradiation when in the presence of 4 or 5 equiv. I^-.

6. Supplementary Note 6: Photocatalytic transformation of tetraarylborates

All reactions were performed in a quartz Schlenk tube and 400 nm LED light (6 w) was used for photocatalysis. For catalytic experiments, tetraarylborates (4.5 μmmol) and photocatalyst **1**-X (X = Tf_2N^-, TfO^-, Cl^- or NO_3^-, 0.45 μmol, 10 mol%) were dissolved in 1.5 mL solvent (CD_3OD, D_2O, CD_3CN, CD_3COCD_3 or DMSO-d_6) in a quartz Schlenk tube under 1.0 atm atmosphere of O_2, air, or N_2, and light irradiation within the photoreactor was given. Yields of products were determined by ^1H NMR spectroscopy using trimethoxybenzene as an internal standard.

6.1 Effect of anions and solvent on photocatalysis

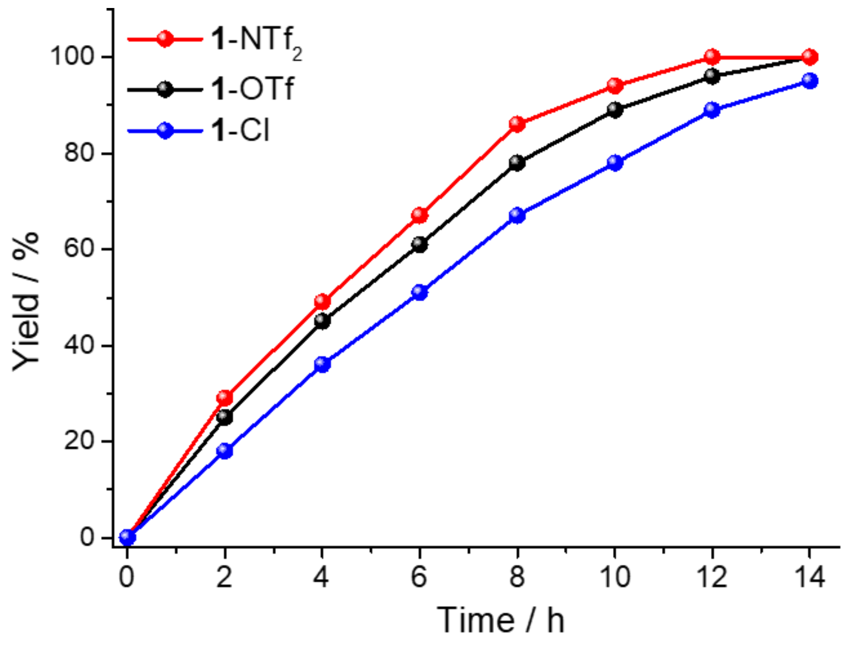

Supplementary Fig. 78 | Photocatalytic transformation of tetraphenylborate into biphenyl and phenol catalyzed by **1**-NTf_2, **1**-OTf, or **1**-Cl. Yield represents for both biphenyl and phenol, which are equal. Conditions: 10 mol% catalyst, 1.0 atm O_2 atmosphere, RT, 6 W LED light at 400 nm.

6.2 Investigation of catalytic mechanism

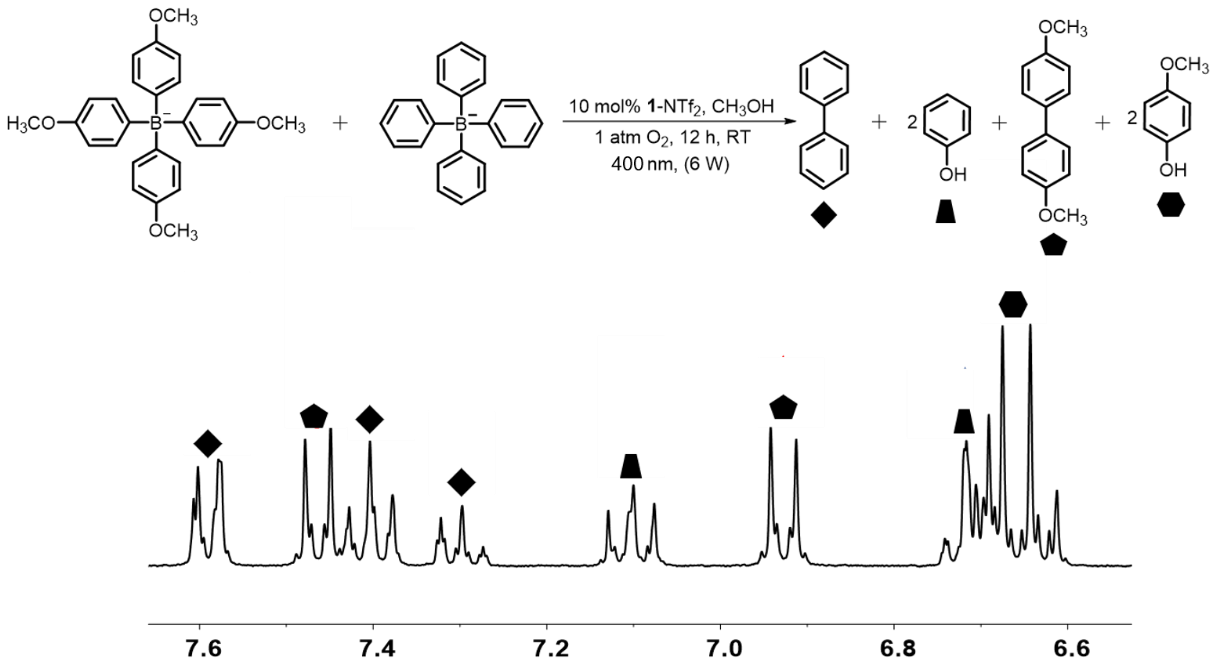

Supplementary Fig. 79 | Photocatalytic transfromation of an equimolar mixture of $[B(Ph\text{-}OCH_3)_4]^-$ and BPh_4^- by **1**-NTf_2, and the corresponding catalytic result monitored by ^1H NMR (400 MHz, 298 K, CD_3OD) spectrum. Only homo-coupling biaryl products were observed.

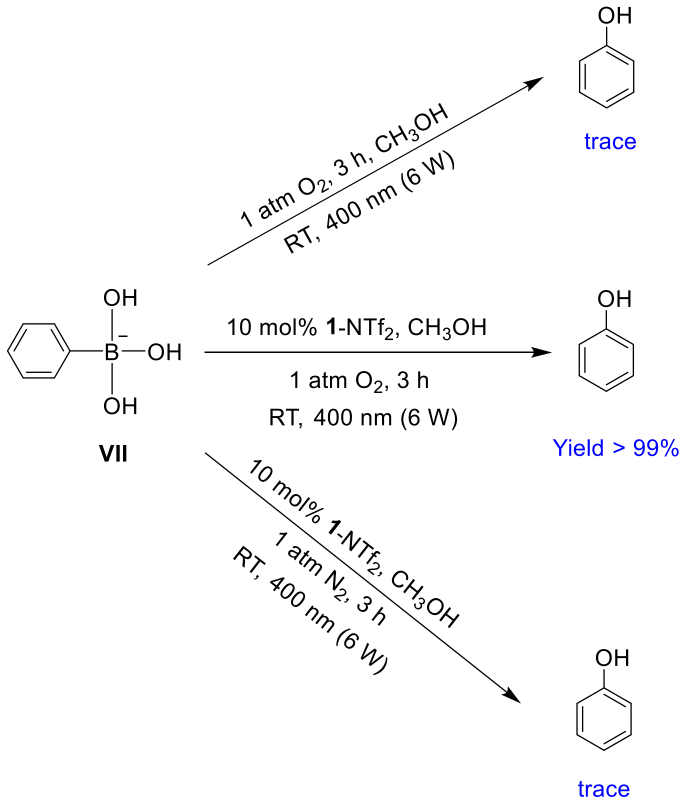

Supplementary Fig. 80 | Oxidative hydroxylation of $BPh(OH)_3^-$ by **1**-NTf_2. Conditions: 10 mol% catalyst, 1.0 atm O_2, RT, 6 W LED light at 400 nm, 3 h.

In order to verify the efficiency of oxidation of intermediate **VII** in Supplementary Fig. 80, which is commercially available, we have conducted catalytic experiments for **VII** using **1**-NTf_2 as the photocatalyst (Supplementary Fig. 80). For this catalytic experiment, $NaBPh(OH)_3$ (4.5 µmmol) and photocatalyst **1**-NTf_2 (0.45 µmol, 10 mol%) were dissolved in 1.5 mL CD_3OD in a quartz Schlenk tube under 1 atm atmosphere of O_2, and light irradiation (400 nm LED light, 6 w) within the photoreactor was given for 3 h. The yield of the product was determined by ^1H NMR spectroscopy using trimethoxybenzene as an internal standard. Results showed complete conversion of **VII** into the phenyl product within 3 h.

7. Supplementary Note 7: Performances of cage in comparison to ligand

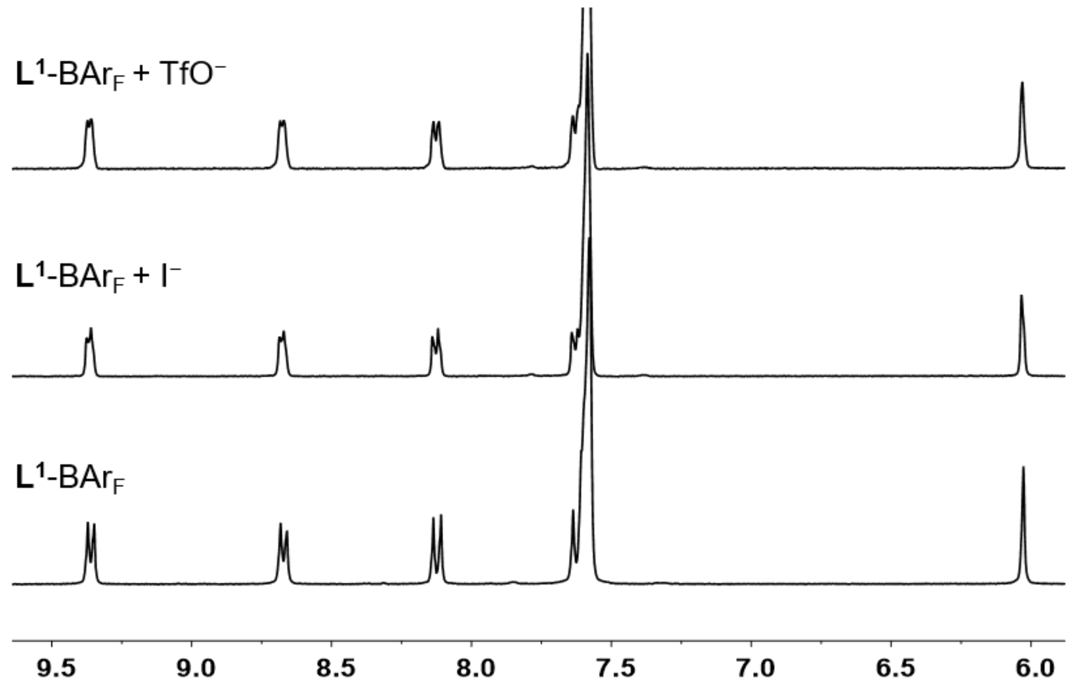

Supplementary Fig. 81 | ^{1}H NMR spectra (CD_3OD, 400 MHz, 298 K) of **L¹**-BAr_F (3 mM) in the absence or presence of 8 mM I^- or TfO^-.

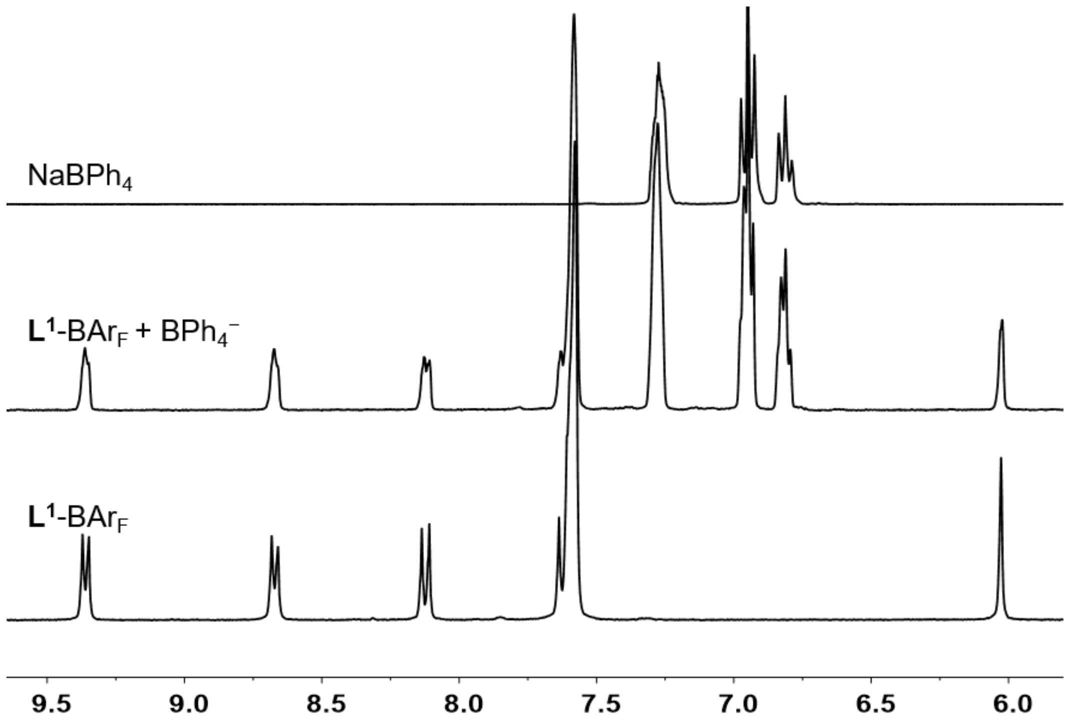

Supplementary Fig. 82 | ^{1}H NMR spectra (CD_3OD, 400 MHz, 298 K) of **L¹**-BAr_F (3 mM) in the absence or presence of 6.0 mM BPh_4^-, and ^{1}H NMR spectrum (CD_3OD, 400 MHz, 298 K) of BPh_4^-.

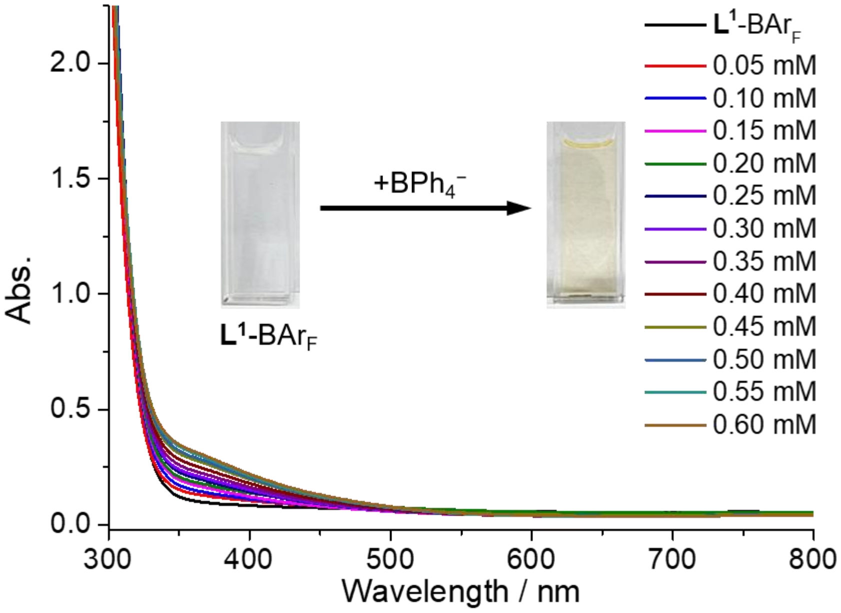

Supplementary Fig. 83 l UV-vis spectrophotometric titrations of BPh_4^- into a methanol solution of **L1**-BAr_F (0.3 mM). Insets: photographs showing the color changes of the solution upon addition of BPh_4^-.

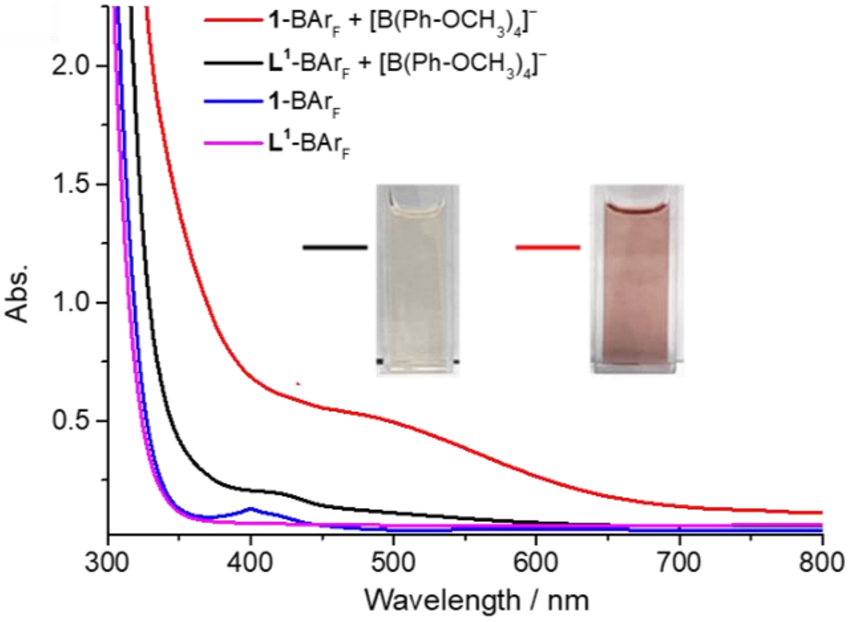

Supplementary Fig. 84 l UV-vis spectra of **1**-BAr_F (0.1 mM in CH_3OH) and **L**$_1$-BAr_F (0.3 mM in CH_3OH) in the absence or presence of 0.6 mM $[B(Ph\text{-}OCH_3)_4]^-$ and the corresponding photographs of the solution after adding $[B(Ph\text{-}OCH_3)_4]^-$.

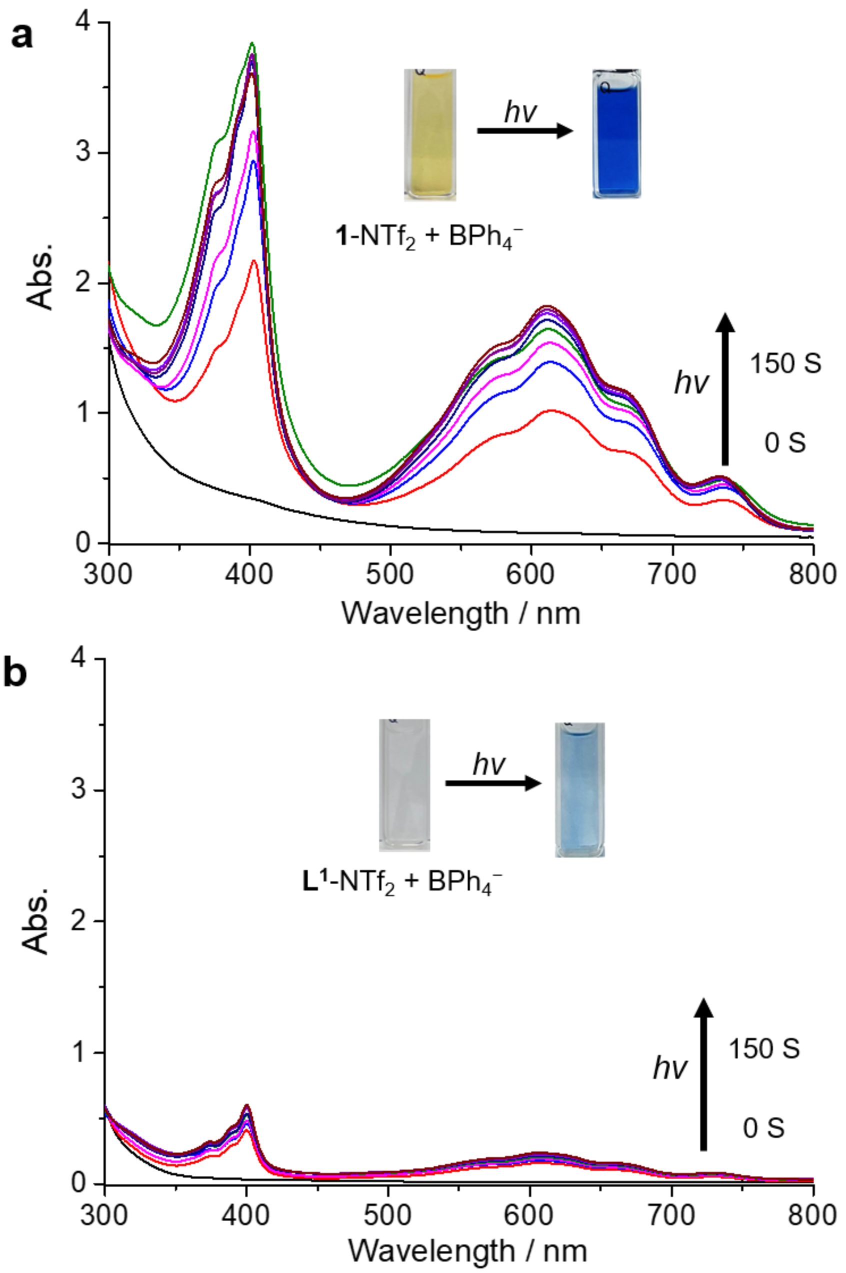

Supplementary Fig. 85 l UV-vis spectra showing the photochromic behavior of **1**-NTf_2 (a) (65 μM) and $\mathbf{L^1}$-NTf_2 (b) (195 μM) in the presence of BPh_4^- (390 μM) in CH_3OH under an atmosphere of N_2.

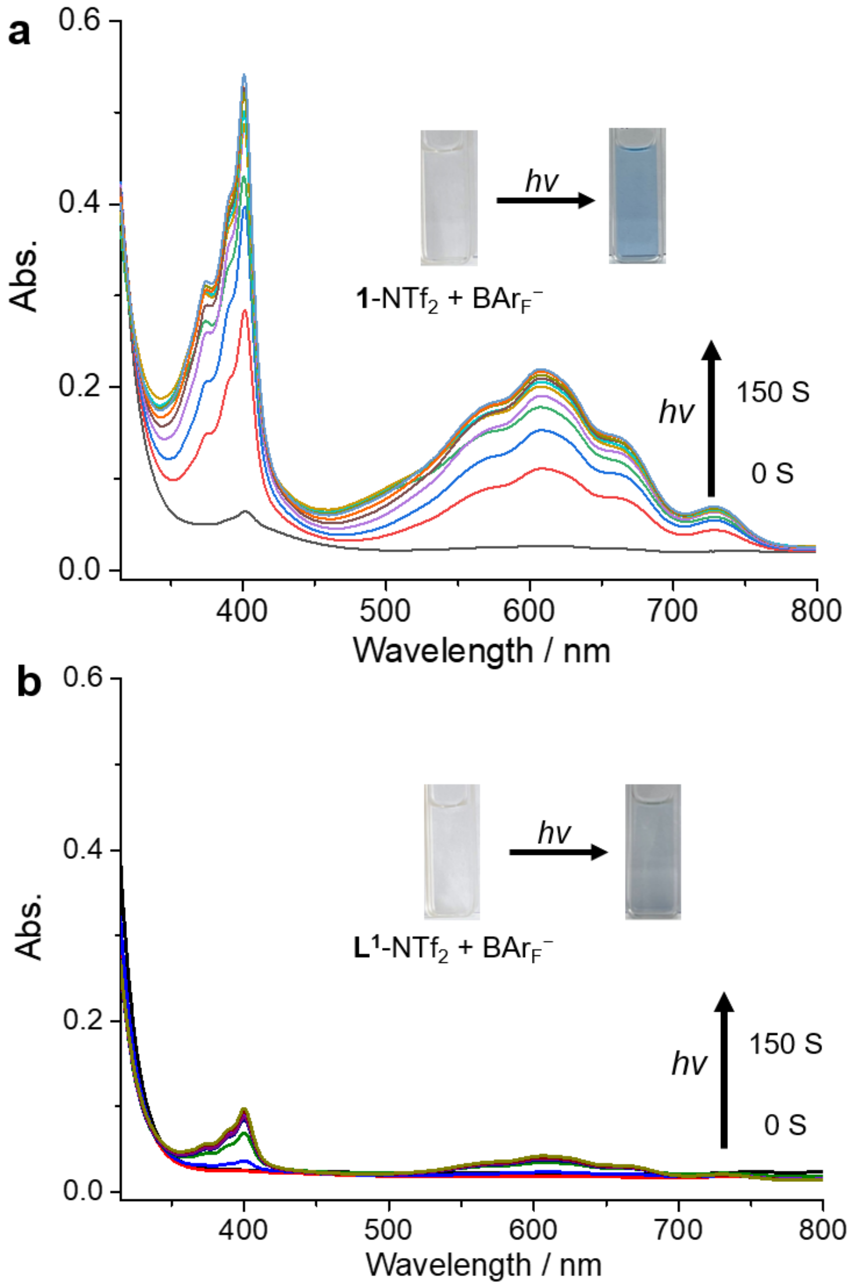

Supplementary Fig. 86 l UV-vis spectra showing the photochromic behavior of **1**-NTf$_2$ (a) (65 µM) and **L^1**-NTf$_2$ (b) (195 µM) in the presence of BAr$_F^-$ (390 µM) in CH_3OH under an atmosphere of N_2.

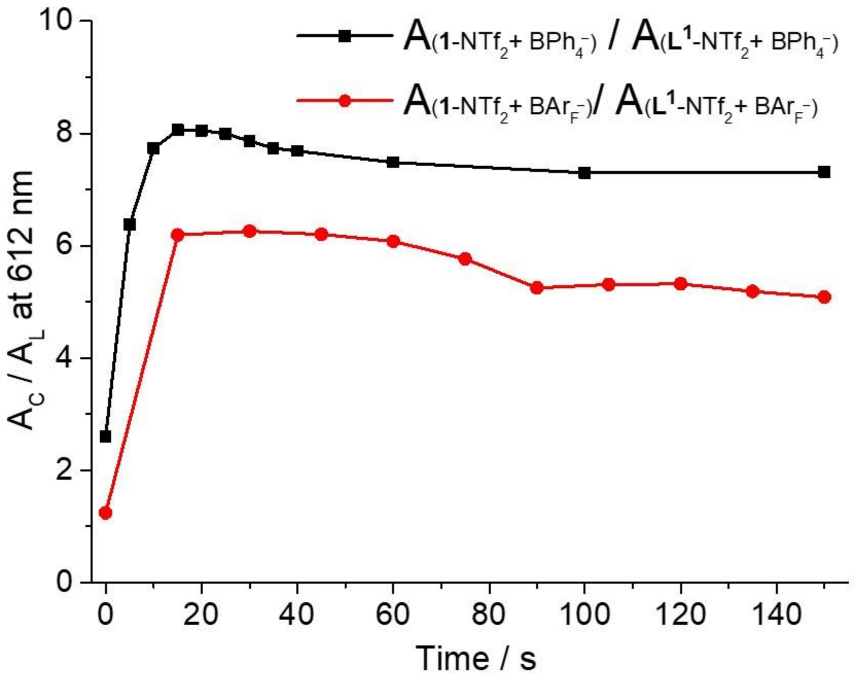

Supplementary Fig. 87 | Time evolution of the ratio of the absorbances at 612 nm between the irradiated cage and ligand (A_C/A_L). Red line: **1**-NTf_2 (65 µM), **L¹**-NTf_2 (195 µM), BAr_F^- (390 µM) in CH_3OH. Black line: **1**-NTf_2 (65 µM), **L¹**-NTf_2 (195 µM), BPh_4^- (390 µM) in CH_3OH.

8. Supplementary References

1. Gong, T. et al. A stable electron-deficient metal-organic framework for colorimetric and luminescence sensing of phenols and anilines. *J. Mater. Chem. A* **6**, 9236-9244 (2018).
2. Li, P. et al. Modulating Excitation Energy of Luminescent Metal-Organic Frameworks for Detection of Cr(VI) in Water. *ACS Appl. Nano Mater.* **2**, 4646-4654 (2019).
3. Li, G. et al. Photoinduced versus spontaneous host-guest electron transfer within a MOF and chromic/luminescent response. *Inorg. Chem. Front.* **8**, 4828-4837 (2021).
4. Sheldrick, G.M. Crystal structure refinement with SHELXL. *Acta Crystallogr., Sect. C: Struct. Chem.* **71**, 3-8 (2015).
5. Spek, A.L. PLATON SQUEEZE: a tool for the calculation of the disordered solvent contribution to the calculated structure factors. *Acta Crystallogr., Sect. C: Struct. Chem.* **71**, 9-18 (2015).
6. http://supramolecular.org/.
